# Chelating Properties of N_6_O-Donors Toward Cu(II) Ions: Speciation in Aqueous Environments and Catalytic Activity of the Dinuclear Complexes

**DOI:** 10.3390/molecules29235708

**Published:** 2024-12-03

**Authors:** Andrea Cendron, Martina Chianese, Kamil Zarzycki, Paolo Ruzza, Claudia Honisch, Justyna Brasuń, Mauro Carraro

**Affiliations:** 1 Department of Chemical Sciences, University of Padova, Via Marzolo 1, 35131 Padova, Italy; 2 Biomolecule Student Science Club, Faculty of Pharmacy, Wroclaw Medical University, 50-556 Wroclaw, Poland; 3 Institute of Biomolecular Chemistry of CNR (ICB-CNR), Padova, Via Marzolo 1, 35131 Padova, Italy; 4 Department of the Basic Chemical Sciences, Wroclaw Medical University, 50-556 Wroclaw, Poland; 5 Institute on Membrane Technology of CNR (ITM-CNR), Unit of Padova, Via Marzolo 1, 35131 Padova, Italy

**Keywords:** copper, SOD activity, catalase activity, peroxidase activity, ligands, chelators

## Abstract

This study focuses on the use of three isostructural N_6_O donor ligands, specifically known to form complexes with copper ions, to chelate Cu(II) from aqueous solutions. The corresponding Cu(II) complexes feature a dinuclear copper core mimicking the active site of natural superoxide dismutase (SOD) enzymes while also creating a coordination environment favorable for catalase (CAT) activity, being thus appealing as catalytic antioxidant systems. Given the critical role of copper dysregulation in the pathophysiology of Alzheimer’s disease (AD), these complexes may help mitigate the harmful effects of free Cu(II) ions: the goal is to transform copper’s reactive oxygen species (ROS)-generating properties into beneficial ROS-scavenging action. This study investigates the speciation, chelating efficiency, and metal selectivity of these ligands, as well as the antioxidant activity of the resulting complexes under aqueous and physiologically relevant conditions. Additionally, the ligands, equipped with functional groups for attaching targeting moieties, are conjugated with a small peptide that may act as an anti-aggregating agent of β-amyloid peptides, aiming to develop a multifunctional therapeutic strategy against Alzheimer’s disease.

## 1. Introduction

Alzheimer’s disease (AD) is the most common form of dementia, accounting for 50–70% of cases and affecting over 35 million people globally [1]. Clinically, AD manifests through memory loss, cognitive decline, and physical disability, eventually leading to death [2].

Several abnormal biochemical processes have been recognized in AD’s brain: (i) aggregation of hyperphosphorylated Tau protein: Tau is a microtubule-associated protein stabilizing the neuronal cytoskeleton. In AD, hyperphosphorylation causes Tau to lose its function, leading to the formation of neurofibrillary tangles (NFTs) and to neuronal death [3]; (ii) release and aggregation of amyloid beta (Aβ) peptides: Aβ peptides are produced from the cleavage of amyloid precursor protein (APP). Under normal conditions, α-secretase and γ-secretase produce non-toxic fragments. However, in AD, β-secretase and γ-secretase generate toxic Aβ fragments that aggregate into extracellular plaques, contributing to neuronal damage [4]; (iii) misplaced metal ions: excessive concentrations of metal ions such as Cu^2+^, Zn^2+^, and Fe^3+^ in the AD brain can promote Aβ aggregation and ROS production, further contributing to neurodegeneration [5]; (iv) oxidative stress: excess of reactive oxygen species (ROS) overwhelms the cellular antioxidant defenses, leading to cellular damage [6].

Within this scenario, ongoing research includes clinical trials for new monoclonal antibodies, β- and γ-secretase inhibitors, anti-Tau therapies, anti-amyloid aggregation agents, metal chelators, and antioxidants [7]. In 2024, the U.S. Food and Drug Administration (FDA) approved the Kisunla (donanemab-azbt) injection for the treatment of Alzheimer’s disease [8]. This drug targets amyloid beta (Aβ) plaques, aiding in their removal, and is intended for patients with early symptomatic AD. Aducanumab, an amyloid-directed monoclonal antibody, was approved in 2021 to target Aβ aggregation, too [9]. Other approved drugs include acetylcholinesterase inhibitors (rivastigmine, donepezil, and galantamine), a glutamate regulator (memantine), and a drug for behavioral symptoms (suvorexant) [10]. These drugs reflect the multi-faceted approach needed to address Alzheimer’s complex pathology.

Metal chelators and antioxidants were also investigated, although with lower success [11]. Antioxidant therapies include free radical scavengers like polyphenols, carotenes, and vitamins [12]. Within this context, mimicking natural antioxidant enzymes would offer a promising way to enhance therapeutic efficacy at lower doses [13]. Key components of the antioxidant defense system are (i) SOD (superoxide dismutase), an enzyme that uses copper and zinc ions to convert superoxide anions into hydrogen peroxide (H_2_O_2_), which is then neutralized by catalase (CAT) or glutathione (GSH) [14]; (ii) CAT, a metalloenzyme that breaks down H_2_O_2_ into water and oxygen, preventing harmful hydroxyl radicals and reducing oxidative damage [15]; (iii) GSH, a tripeptide that works with NADPH to detoxify reactive oxygen species [16].

Among enzyme mimetic compounds (or synzimes), M40403 is the first SOD mimetic to enter clinical trials, demonstrating a high catalytic rate [17]. Additionally, [Mn(bpia)]_2_ shows CAT-like activity with a notable rate constant [18], while other non-heme manganese or iron complexes exhibit dual activity, enhanced solubility, and self-protection against oxidative stress [19]. Moreover, copper complexes with both SOD and CAT-like activity were reported, highlighting their potential as versatile dual-functional antioxidants and broadening their applications in oxidative stress mitigation [20].

**Scheme 1:**
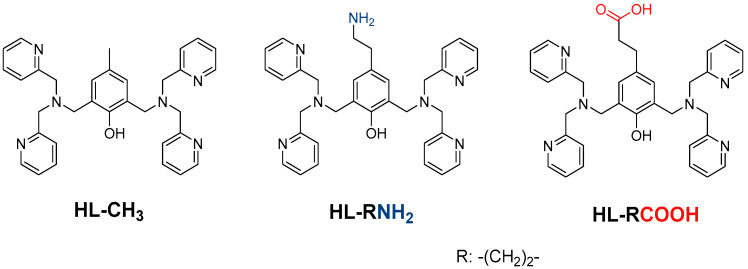
The three ligands—derived from 2,6-bis[(bis(2-pyridylmethyl)amino)methyl]-4-methylphenol (HL-CH_3_)—used in this study.

In this work, three functional N_6_O ligands (Scheme 1) were explored as multitarget drugs integrating metal chelation, anti-aggregation, and antioxidant properties. A major effort was dedicated to investigating the speciation of the simplest one, the 2,6-bis[(bis(2-pyridylmethyl)amino)methyl]-4-methylphenol, to assess the capability to form dinuclear complexes in a neutral aqueous environment.

## 2. Results

The three ligands in Scheme 1 were selected to replicate the active site of these natural enzymes. As described in the Supporting Information, the ligands syntheses utilize a common reagent, di-(2-picolyl)amine (DPA). Each ligand contains a central p-substituted phenol core, which is linked by methylene groups to two DPA units, resulting in two chelating N_3_O donor sets. Each donor set is composed of three nitrogen atoms from the DPA moieties and a single oxygen atom shared through the phenolic bridge.

The ligands were synthesized and characterized by ^1^H-NMR, ESI-MS(+), UV–Vis, and FT-IR spectroscopies (see Supporting Information, Figures S3–S20). The corresponding dinuclear copper complexes were then synthesized (see Supporting Information) according to the general procedure proposed by Torelli et al. [21]: a solution with two equivalents of Cu(ClO_4_)_2_ in water was added dropwise to a solution of the ligand in acetone and left under stirring at room temperature. The complexes were then isolated and characterized by ESI-MS(+) spectrometry, FT-IR and UV–Vis spectroscopies, and elemental analysis (Figures S21–S29). These complexes are all soluble in water (>1.5 mM), especially those bearing the amino (HL-RNH_2_) and the carboxylic-functional groups (HL-RCOOH). Based on the X-ray data reported in the literature for the complex [Cu_2_(L-CH_3_)(H_2_O)_2_](ClO_4_)_3_ [21], each copper atom is likely coordinated by three nitrogen atoms (one from the tertiary amine and two from pyridine groups) and two oxygen atoms—one from the phenoxo bridge and the other from a water molecule. This pentacoordinated geometry can be described as a slightly distorted square pyramid [21].

### 2.1. Speciation in Aqueous Environment

The ligands are designed to chelate copper(II) ions in an aqueous environment, specifically targeting Cu(II) excess resulting from dyshomeostasis in AD brains. To monitor their behavior in solution, the acid-base properties and pH-dependent coordination abilities of the ligand L-CH_3_ towards Cu(II) ions were thoroughly investigated. Additionally, the binding constants of the three ligands with Cu(II) ions were determined and compared. At the same time, since they should not unbalance the concentration of other metals, the preferential binding of these ligands for copper over other common divalent metals was also evaluated.

**Figure 1:**
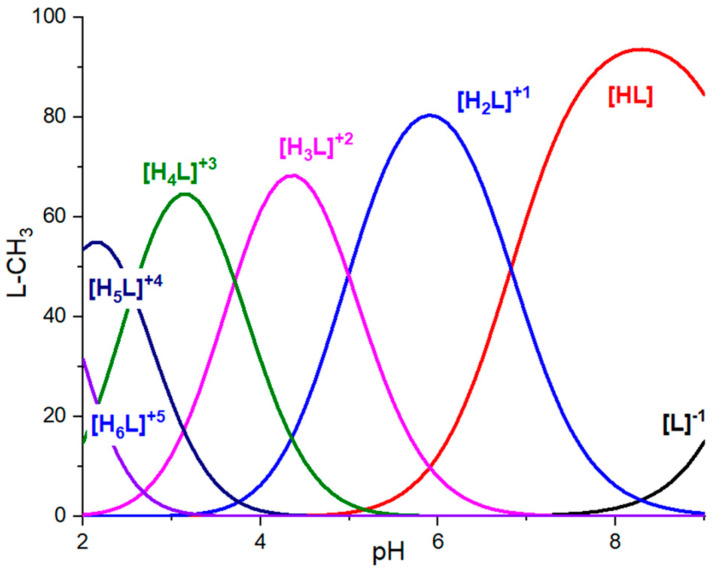
The speciation curves for the free ligand L-CH_3_. Conditions: [ligand] = 0.043 mM in DMSO (5% *v*/*v*)/H_2_O (HCl, KCl); starting pH = 2.7.

As far as the ligand behavior in aqueous solutions is concerned, the speciation curves were obtained from the titration of an L-CH_3_ solution (0.1 mM in DMSO 5% (*v*/*v*)/H_2_O (HCl/KCl); initial pH = 2.7) by stepwise addition of NaOH 0.1 M. The distribution curves and the acid association constants were then calculated (Figure 1 and Table 1).

At low pH, L-CH_3_ has seven protic groups that can be deprotonated upon pH increase: besides the phenolic OH, two tertiary amines and four pyridines. From the titration, six logK_a_ could be successfully obtained. The highest one, logK_a_ = 9.75, is related to the phenolic group [22]. The next constants are ascribed to the nitrogens of the tertiary amines and of the Pyr groups, although they are difficult to unambiguously correlate with a specific functional group. The speciation curve shows that the main species at pH 7.4 is the neutral HL-CH_3_, where the OH group is the only one retaining its proton. The potentiometric titration was carried out till pH 9.5 to avoid the precipitation of the ligand in the basic environment.

The next step was the analysis of the system with L-CH_3_ and Cu(II). For a better investigation of the binging abilities of the L-CH_3_ ligand, the titration was performed both for the Cu(II):L-CH_3_ molar ratio = 1:1 and for 2:1. The overall (logβ_i_) and stepwise (logK_i_) protonation constants of in situ-formed complexes are collected in Table 2. Depending on pH, L-CH_3_ forms mono- and di-nuclear complexes with Cu(II) ions, their relative abundance depending on the metal-to-ligand molar ratio.

**Figure 2:**
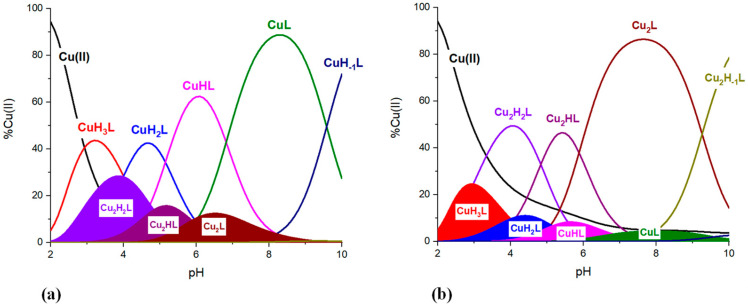
The formation of the in situ-formed Cu(II) complexes at different molar ratios: (**a**) nCu(II):n*L* = 1:1, (**b**) nCu(II):n*L* = 2:1. *L* = L-CH_3_, with concentration = 0.7 mM in DMSO (5% *v*/*v*)/H_2_O (HCl, KCl); starting pH = 2.7.

Figure 2 shows the formation of the subsequent complexes in the investigated systems in relation to the Cu(II):L-CH_3_ molar ratio.

**Scheme 2:**
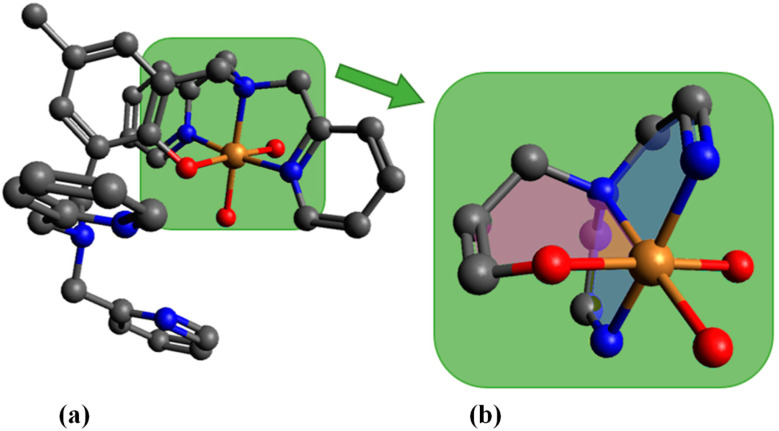
(**a**) The visualized structure of the Cu*L*, where *L* = L-CH_3_ complex with the {3N, O_Ph-O,_ 2H_2_O} binding mode, and (**b**) inset of the chelating rings present in the metal core. For clarity, the hydrogen atoms are removed from the structure. Cu: dark yellow, O: red, N: blue, C: gray.

In the system with equimolar amounts of Cu(II) and L-CH_3,_ the dominant species are mononuclear complexes (Figure 2a). Between pH = 2.5 and pH = 10, five mononuclear complexes appear in the system: CuH_3_*L*, CuH_2_*L*, CuH*L*, Cu*L*, and CuH_−1_*L* (where *L* = L-CH_3_). In these conditions, the dinuclear complexes such as Cu_2_H_2_*L*, Cu_2_H*L*, and Cu_2_*L* are also present. However, their concentration is lower than 25%. Moreover, based on the acid-base abilities of the L-CH_3_ it can be concluded that N-donors are involved in the metal ion binding mainly below pH 7. Between pH 7 and 8, the dominant is the complex Cu*L*. The stepwise constant for the reaction [CuH*L*]^2+^ ⇌ [Cu*L*]^+^ + H^+^ (logK = 6.82, Table 2) is significantly lower than the logK_a_ of the Ph-OH residue. Based on this fact, it can be assumed that the dissociation of the proton from the phenolic group is related to the involvement of the O_Ph-O_ in the coordination of the Cu(II) ion. Owing to this result and literature data [21], the {N_tetriary_, 2N_Pyr_, O_Ph-O,_ 2H_2_O} binding mode of the Cu*L* complex is the most likely one. Scheme 2a shows the proposed structure of this complex visualized using the Avogadro program [23].

The analysis of the structure shows that the formed complex is characterized by the presence of three chelating rings. Two 5-membered chelating rings with atoms Cu, N_tetriary_, C, C, N_Pyr_, and one 6-membered with Cu, N_tetriary_, C, C, C, O_Ph-O_ (Scheme 2b). Finally, the mononuclear CuH_−1_*L* complex appearing in the system is likely related to the deprotonation of one of the metal-bound water molecules.

**Scheme 3:**
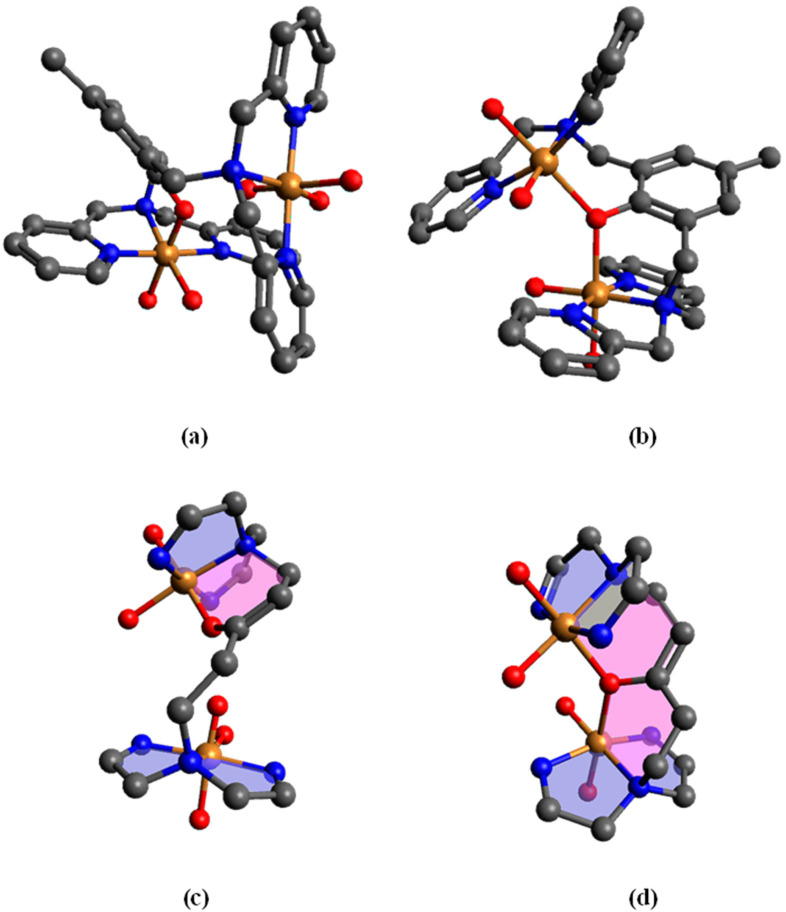
The visualized possible structures of the Cu_2_*L* (*L* = L-CH_3_) complex with: (**a**) the {N_tetriary_, 2N_Pyr_, O_Ph-O_, 2H_2_O}/{N_tetriary_, 2N_Pyr_, 3H_2_O}, (**b**) 2× {N_tetriary_, 2N_Pyr_, O_Ph-O_, 2H_2_O} and their corresponding chelating rings in (**c**) and (**d**), respectively. The protons were removed to clarify the structure. Cu: dark yellow, O: red, N: blue, C: gray.

Concerning the system with the nCu(II):n*L* = 2:1 molar ratio, the dominant species are the dinuclear complexes (Figure 2b), as obtained from the constants collected in Table 1, highlighting a different behavior. At pH 3.5, the prevalent species is the dinuclear one (Cu_2_H_2_*L*) with the protonated ligand. Upon increasing the pH, the formation of [Cu_2_H*L*] occurs, while at pH 7.8, the more stable form is Cu_2_*L*. Similarly to the system discussed above, the formation of this species can be related to the deprotonation of the phenolic group and binding of the O_Ph_ donor to the Cu(II) ion. There are two main possibilities for copper ions binding in the Cu_2_*L* species. The first features the {N_tetriary_, 2N_Pyr_, O_Ph-O,_ 2H_2_O}/{N_tetriary_, 2N_Pyr_, 3H_2_O} binding mode (Scheme 3a), whilst the second one is characterized by the 2× {N_tetriary_, 2N_Pyr_, O_Ph-O_, 2H_2_O} donor set (Scheme 3b). Based on the results obtained, it is still difficult to unambiguously define which coordination manner occurs in solution.

Both proposed complexes are characterized by different numbers of chelating rings. In the first proposed structure, it can be observed that one Cu(II) is involved in the formation of three chelating rings whilst the second Cu(II) is involved in two chelating rings (Scheme 3a,c). In the second complex (Scheme 3b,d), both copper ions form three chelating rings, which may support a higher probability of the existence of this species, being also in good agreement with known crystal structures. Above pH 9, the formation of the last complex likely involves the loss of a proton from the metal-bound water molecule. In these conditions the mononuclear species are also present, but their concentrations are relatively lower.

### 2.2. Determination of the Binding Constants

The global reaction for the complexation reaction, where one mole of ligand chelates two moles of metal ions, is shown in Equation (1).

(1) $$\begin{array}{cc} L^{-}+M^{2+}⇌{\left[M\left(L\right)\right]}^{+} & K_{1}\\ {\left[M\left(L\right)\right]}^{+}+M^{2+}⇌{\left[M_{2}\left(L\right)\right]}^{3+} & K_{2}\\ L^{-}+2M^{2+}⇌{\left[M_{2}\left(L\right)\right]}^{3+} & \beta_{1,2}=K_{1}K_{2} \end{array}$$

**Figure 3:**
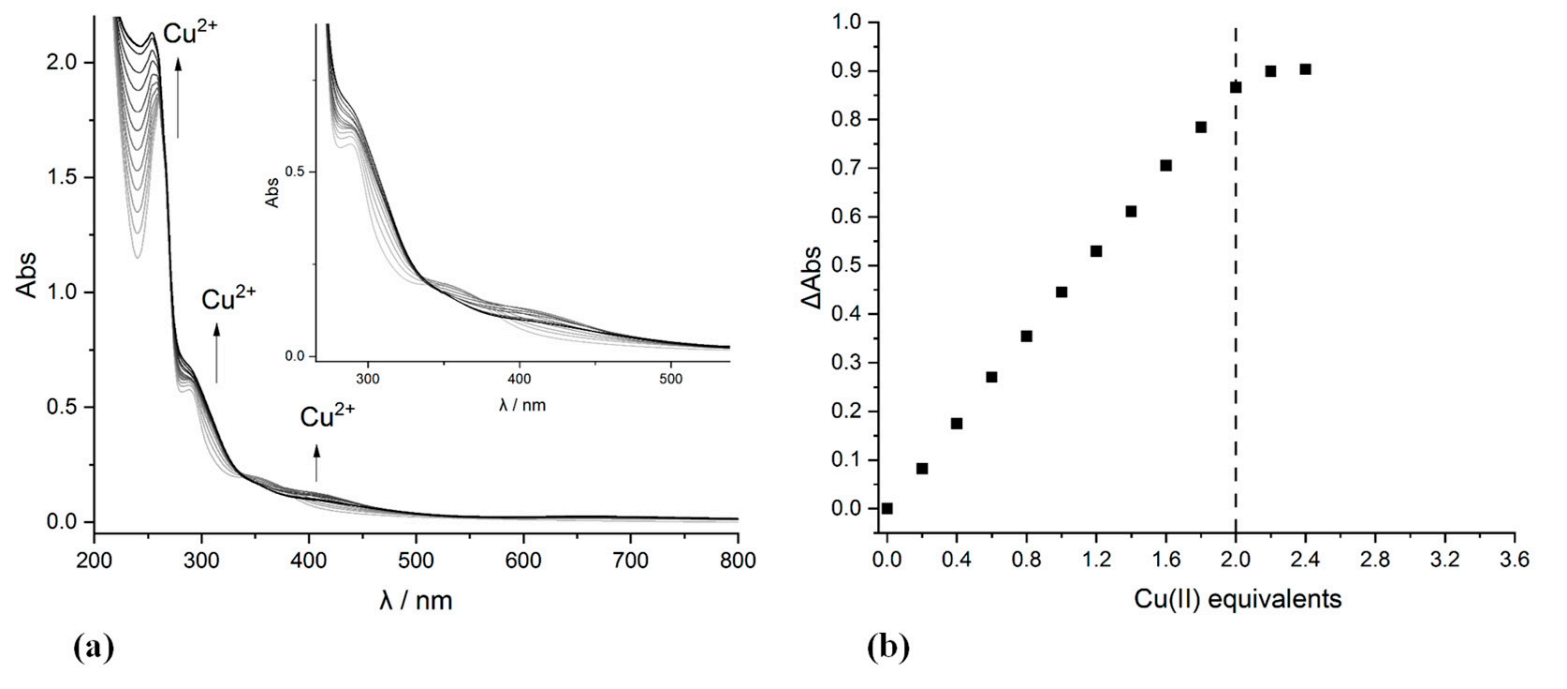
(**a**) UV–Vis spectra of HL-CH_3_ titration with Cu(ClO_4_)_2_. Ligand concentration is 0.1 mM; each addition corresponds to 0.2 equiv. of Cu^2+^. An isosbestic point at 340 is observable; (**b**) plot of ΔAbs at 240 nm vs. Cu(II) equivalents (square symbols) for the titration of HL-CH_3_. The dotted vertical line highlights the saturation behavior when ~2 equiv of Cu(II) are added.

To determine the overall equilibrium constant β_1,2_ (K_bind_), a solution of the ligand was titrated with aliquots of 0.2 equivalents of Cu(II). Monitoring the formation of the complex via UV–Vis spectroscopy, the absorbance around 260 nm, 300 nm, 450 nm, and 700 nm increased during the titration (Figure 3a) in all cases. The latter two bands are visible only at high concentrations (>0.1 mM). These changes are caused by the formation of the complex and the disappearance of the free ligand.

In Figure 3b, the linear increase in the absorbance change (ΔAbs) at 240 nm, upon an increase in Cu(II) amount, is evident, leading to a sharp plateau around 2 equivalents. Similarly, the maximum is at around 2 equivalents also for the propylic acid and the aminoethyl-substituted complexes (Figures S36–S38). The binding constants were determined by non-linear regression analysis of Equation (2),

(2) $$\frac{∆A}{b}=\frac{\left(K_{1}∆A_{1}\left[Cu\right]+{\;\beta }_{1,2}∆A_{2}{\left[Cu\right]}^{2}\right)}{1+K_{1}\left[Cu\right]+{\;\beta }_{1,2}{\left[Cu\right]}^{2}}$$

which traces the correlation “ΔAbs/b vs. Cu(II) concentration,” according to K. Connors [24]. In this equation, ΔA is the differences in absorbance at a certain wavelength, b is the optical path (1 cm), K_1_ is the constant for the formation of the complex 1:1, and β_1,2_ is the global formation constant (K_bind_) for the complex 1:2. ΔA_1_ and ΔA_2_ are the differences in absorbance due to the formation of the 1:1 complex and the 1:2 complex, respectively. These unknown parameters can be estimated graphically from the plot, where the equivalents of copper added are 1 and 2, respectively. Plotting the titration using 0.1 mM of ligand gave a sharp plateau, missing the non-linear portion and lacking useful information. This happens when the 1/K_bind_ is much bigger than the concentration of the ligand [25]. For this reason, a smaller concentration of ligand was used (1 µM). Table 3 summarizes the binding constants calculated for the three complexes obtained from plots reported in Figures S36–S38.

The complex [Cu_2_(L-RNH_2_)(H_2_O)_2_](ClO_4_)_3_ has a relatively higher formation constant. Since the binding constant between Aβ and Cu(II) at pH 7.4 is around 10^7^–10^8^ [26], these ligands may be able to strip away the copper from the amyloids to prevent further metal-mediated aggregation. On the other hand, Cu(II)-Aβ represents a labile pool of copper in vivo, and it should be noted that the presence of other metal proteins or competing ligands might limit the effectiveness of the metal chelators of the herein-prepared ligands [26].

### 2.3. Evaluation of Binding Selectivity

To evaluate the selectivity of these ligands towards copper, the same titrations were performed with different metal salts: Fe(ClO_4_)_2_, Mn(ClO_4_)_2_, and Zn(ClO_4_)_2_, monitoring the methyl-substituted ligand (Figures S39–S41). These metal ions were chosen because they are also divalent and common in biological matrixes. The binding constants between the ligand HL-CH_3_ and the different metal ions were calculated and are reported in Table 4.

Comparing these values with the K_bind_ obtained for the copper complex, they all correlate with the Irving–Williams Series for pentacoordinated complexes (Figure S42). Generally, the Irving–Williams Series states that for octahedral high-spin complexes, the stability increases across the first-period transition metals, with a maximum at copper: Mn(II) < Fe(II) < Co(II) < Ni(II) < Cu(II) > Zn(II). This is regardless of the nature of the ligand. However, in this case, the complexes are pentacoordinated in a distorted square pyramidal geometry, and the series may have a slightly different trend [27].

Interestingly, a selectivity for Cu(II) binding could be observed under ESI-MS(+) conditions. Equimolar solutions of Cu(ClO_4_)_2_, Fe(ClO_4_)_2_, Mn(ClO_4_)_2_, and Zn(ClO_4_)_2_ were prepared, and half an equivalent of HL-CH_3_ was added. The composition of the solution was then analyzed with ESI-MS to determine which species were formed. The peaks present in the obtained spectrum (Figure S43) are only ascribable to the copper complex. This is expected as almost three orders of magnitude are present between the stability constant for copper and the one for other metals. The most intense signal is the cluster at *m*/*z* = 855, referring to the species [Cu_2_(L-CH_3_)(ClO_4_)_2_]^+^. Other signals are at *m*/*z* = 801 for [Cu_2_(L-CH_3_)(COO)(ClO_4_)]^+^ and *m*/*z* = 351 for [Cu_2_(L-CH_3_)(ClO_4_)]^2+^. Although further experiments will be required to confirm the selectivity in physiological conditions, these results suggest the preferential binding of copper under electrospray conditions, with no mixed metal coordination occurring.

### 2.4. Catalytic Activity of the Complexes

The catalytic activity for the three complexes was then evaluated. Firstly, their redox properties were analyzed through cyclic voltammetry, and then catalytic tests were performed to evaluate their SOD- and CAT-like activity. Finally, the undesired pro-oxidants or peroxidase-like activity was also evaluated.

#### 2.4.1. Cyclic Voltammetry

Cyclic voltammetry can be a useful tool for making preliminary assumptions about the catalytic activity of the complexes, especially for SOD mimicking. Since the standard reduction potential for the couple O_2_/O_2_^●−^ is −0.33 V and for the couple O_2_^●−^/H_2_O_2_ is +0.89 V, if the metal in the complex has a reduction potential that lies in between these two values, it may catalyze the disproportion of the superoxide anion [28]. As a matter of fact, many natural SOD enzymes have a reduction potential between 0.2 and 0.4 V.

**Figure 4:**
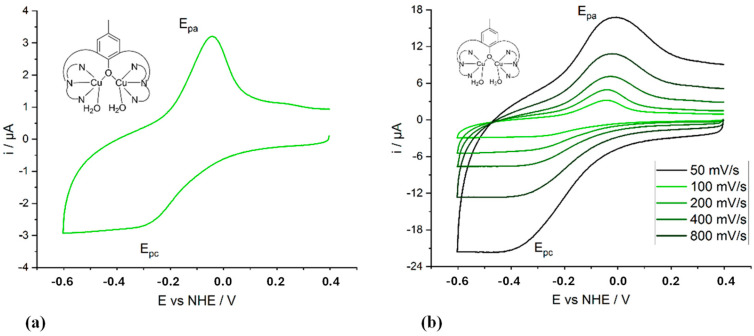
Cyclic voltammograms of the complex [Cu_2_(L-CH_3_)(H_2_O)_2_](ClO_4_)_3_ 0.25 mM in phosphate buffer saline (PBS, 10 mM sodium phosphate, 150 mM NaCl, pH 7.8). Scan rate 50 mV/s (**a**) and different scan rates (**b**).

In Figure 4 and Figure S44, the voltammograms of the complexes with potentials referred to the NHE (V_NHE_ = V_Ag/AgCl(3 M KCl)_ + 0.197 V) are shown.

In the voltammograms, two peaks can be distinguished, the anodic and the cathodic (in positive and negative potential, respectively), describing non-reversible electronic processes, since the peak-to-peak separation (ΔE = E_pc_ − E_pa_) is larger than 57 mV (or 28.5 mV for a two-electron transfer process). For every complex, the half-peak potential (E_1/2_), attributable to the redox couple Cu_2_^II,II/I,I^ in a dielectronic process, was calculated as

$$E_{1/2}=\frac{E_{pc}+E_{pa}}{2}$$

As reported in Table 5, every reduction potential is in the correct range to catalyze the dismutation of the superoxide radical.

#### 2.4.2. Superoxide Dismutation-like Activity (SOD)

The ligands have an N_3_O donor set for each copper ion, formed by two pyridines, the aminic bridge, and the phenol moiety; the coordination is completed by a water molecule. Similarly, the Cu/Zn-SOD enzyme has four histidine residues and the water molecule. The geometry is also the same, a distorted square pyramid geometry. To evaluate the SOD-like activity of the complexes, the method developed by McCord and Fridovich [29] was used. In this indirect competitive enzymatic assay, the superoxide radical anions are generated in situ by the xanthine/xanthine oxidase system, where the enzyme converts xanthine to urate and O_2_^●−^. Another enzyme, cytochrome c, oxidizes the superoxide anion to oxygen, with a parallel reduction to ferricytochrome c, which absorbs at 550 nm. This assay was performed in a cuvette of 3 mL of volume and 1 cm pathlength, monitoring the ferricytochrome c absorbance over time. The curves reach the plateau when all the cytochrome c is converted to ferricytochrome c. When the copper complex is active, it competes with the enzyme, and the conversion is not complete, leading to a lower absorbance (Figures S45–S47). Finally, the kinetic constants for the complex (k_SOD_) are obtained from Equation (3), where the constant for the cytochrome c k_CytC_ is known from the literature [29].

(3) $$k_{SOD}\left(O_{2}^{•-}\right)=\frac{k_{CytC}\;\left(O_{2}^{•-}\right)}{{IC}_{50}}$$

As anticipated by the CV data, all the complexes have SOD-like activity. The complex with the highest rate constant and the lowest IC_50_ for the dismutation of the superoxide radical anion is the one bearing the p-ethylamine substituent, with minor differences for the other two complexes (Table 6). If compared with values reported in the literature (Table S2), the complexes have moderate activity [13,18,20,30,31,32,33,34]. On the other hand, for many complexes, only the IC_50_ values were reported, and they can be very different depending on the method used for the measurement.

IC_50_ and log(k_SOD_) values for the three synthesized complexes were measured in PBS (50 mM sodium phosphate, 150 mM NaCl) at pH 7.8 and 25 °C.

#### 2.4.3. Catalase-like Activity (CAT)

Catalase enzymes disproportionate hydrogen peroxide into water and dioxygen very effectively (k_cat_ ~ 10^7^ M^−1^ s^−1^) [35]. Over the years, many artificial and biomimetic CAT complexes have been studied. The most efficient are manganese complexes [18,31,36], but also copper complexes are reported for high catalase-like activity [37,38,39].

To measure the CAT-like activity, the pressure resulting from O_2_ production was monitored. In this assay, a solution of the complex and hydrogen peroxide in (non-coordinating) borate buffer (pH 7.8) was connected to a pressure transducer for 22 h. The difference in pressure obtained was then related to the µmol of oxygen produced with the “Ideal gas law” (Equation (4)).

(4) $$n\left(O_{2}\right)=\frac{∆P\cdot V}{R\cdot T}$$

**Figure 5:**
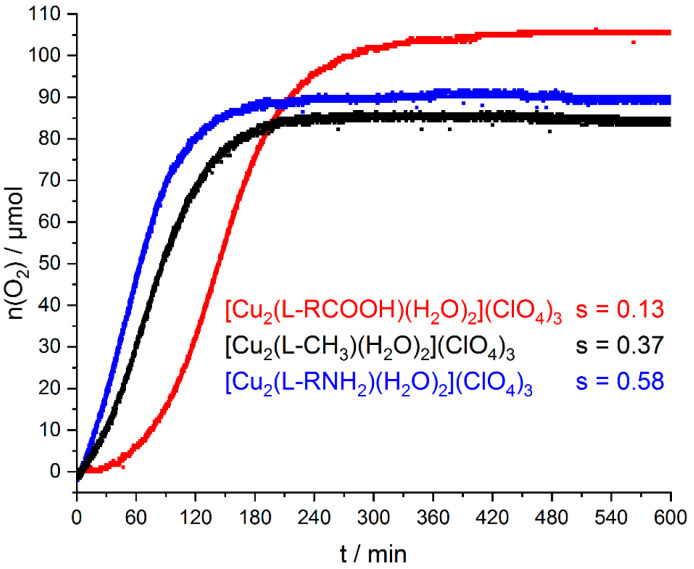
O_2_ evolution from hydrogen peroxide vs. time for the CAT-like activity assay. The values of slope (s) are calculated by linear regression before conversion of 10% of hydrogen peroxide and are equal to R_0_. [Complex] = 200 µM, [H_2_O_2_] = 22.5 mM in borate buffered saline (BBS, pH 8.2), T = 25 °C, and head-space of 12.98 mL. Note that slopes are in µmol min^−1^. Dividing by the volume and by 60 gives it in µM O_2_ s^−1^.

From these values, the graph “µmol of O_2_ vs. time” was plotted (Figure 5). For the complex bearing the acid moiety, the linear range started after a longer induction period.

The compound with the highest CAT-like activity is [Cu_2_(L-RNH_2_)(H_2_O)_2_](ClO_4_)_3_, removing most of the hydrogen peroxide in less than 3 h. Therefore, the same complex shows both the highest SOD- and CAT-like activity.

Meanwhile, [Cu_2_(L-RCOOH)(H_2_O)_2_](ClO_4_)_3_ reveals a relatively long induction time (30 min) before starting to evolve oxygen, suggesting a likely rearrangement in the complex geometry. For the other two complexes, this induction time is much less evident. Nevertheless, all the analyzed complexes completely removed hydrogen peroxide, as confirmed by starch-potassium iodide papers after each experiment.

The rate constant for the reaction of disproportion was calculated as reported for similar complexes, where k_CAT_ was obtained by linear regression of pseudo-first-order rate constants (Equation (5)).

(5) $$k_{obs}=k_{CAT}\left[H_{2}O_{2}\right]$$

Pseudo-first-order reactions can be considered when the concentration of complex is kept constant and [H_2_O_2_] >> [complex]. Measuring the same kinetics by changing only the concentrations of hydrogen peroxide gives different observable rate constants (k_obs_). From the plot “k_obs_ vs. [H_2_O_2_]”, k_CAT_ is obtainable as the slope of the curve (Figure S48).

The values for TON and TOF are, respectively, the ratio between the moles of oxygen produced, or R_0_, and the moles of catalyst, as visible in Equations (6) and (7).

(6) $$TON=\frac{n(O_{2})}{n(catalyst)}$$

(7) $$TOF=\frac{R_{max}}{n(catalyst)}$$

Table 7 summarizes the values for R_0_, Rmax, TOF, TON, k_CAT_, and moles of O_2_ produced in the CAT assay.

In Table S3, a comparison with other complexes present in the literature with CAT-like activity is shown. While Mn-based compounds exhibit incomparable performances, Cu(II)-based compounds have similar activity [13,20,40,41,42]. In particular, [Cu_2_(L-RNH_2_)(H_2_O)_2_](ClO_4_)_3_ has the highest k_CAT_ among the three tested complexes, with only a few complexes showing a much greater efficiency in the dismutation of hydrogen peroxide.

In one experiment, a second addition of hydrogen peroxide was made to the same solution of the most active [Cu_2_(L-RNH_2_)(H_2_O)_2_](ClO_4_)_3_ (Figure S49). Also, in this case, all the peroxide was removed completely, reaching a plateau, but it required a longer period of time (~25 h, R_0_ = 0.15 µmol min^−1^/0.21 µM^−1^ s^−1^). This different behavior is thought to be caused by the partial degradation of the complex, which is no longer as active as at the beginning of the first assay. The degradation is confirmed by UV–Vis spectra, which showed a decrease in the LMCT band, as well as shifts and fading of the bands (Figures S50–S52). Noteworthy, the complex with L-RNH_2_ appeared to be the most stable.

#### 2.4.4. Peroxidase-like Activity (Pro-Oxidant Activity)

**Scheme 4:**
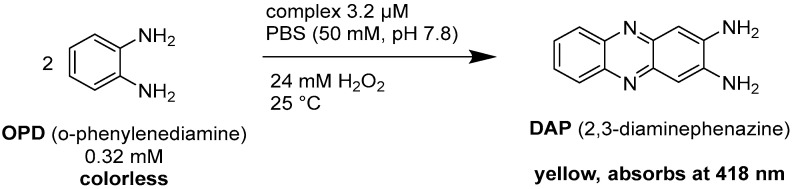
Reaction scheme for the oxidation of OPD to DAP.

**Figure 6:**
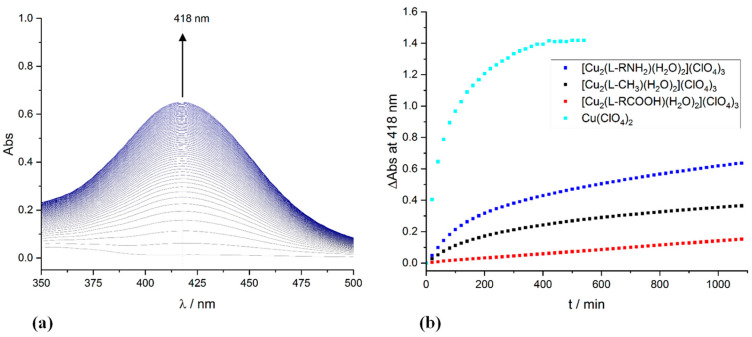
(**a**) Variation in absorbance at 418 nm vs. time (18 h, a spectrum every 20 min) for the complex [Cu_2_(L-RNH_2_)(H_2_O)_2_](ClO_4_)_3_ in the peroxidase-like assay. (**b**) Trend over time for absorption of DAP at 418 nm. Cu(ClO_4_)_2_ (light blue), [Cu_2_(L-RNH_2_)(H_2_O)_2_](ClO_4_)_3_ (blue), [Cu_2_(L-CH_3_)(H_2_O)_2_](ClO_4_)_3_ (black), and [Cu_2_(L-RCOOH)(H_2_O)_2_](ClO_4_)_3_ (red). The peroxidase-like assay was repeated twice for each complex and also with copper perchlorate as a control experiment.

The pro-oxidant activity is named after another enzyme, the peroxidase. It is important to underline that this is a drawback of the studied complexes. Peroxidase, such as the already described GSH, decomposes hydrogen peroxide, producing water, but at the cost of oxidizing another substrate. Similarly, the complexes described here could remove hydrogen peroxide, catalyzing the oxidation of an organic substrate and leading to an enhancement of oxidative stress. Since all the catalytic measurements made so far were in the absence of an oxidizable substrate (excluding the catalyst itself), it is important to also verify this phenomenon. For measuring the peroxidase-like activity, the substrate o-phenylenediamine (OPD) was oxidized to 2,3-diaminephenazine (DAP) by H_2_O_2_ in the presence of copper ions (Scheme 4) [43]. While OPD is colorless and non-fluorescent, DAP is a yellow molecule, absorbing at 418 nm. This variation in absorbance was monitored for 18 h via UV–Vis spectroscopy. The reaction is shown in Figure 6, and it may occur through different mechanisms, including the activation of Fenton-like radical reactions.

The peroxidase-like activity can be evaluated by plotting “Abs at 418 nm vs. time,” as reported in Figure 6b. The complex with the highest pro-oxidant activity is the complex bearing the ethylamine substituent, while the others display a lower activity. It is also evident, however, that the pro-oxidant activity of free copper ions is much higher than the one found for complexes, demonstrating the possible advantage of the chelation.

### 2.5. Anti-Amyloid Aggregation Ability

To evaluate the anti-aggregation ability of the complexes, the β-amyloid 1–40 peptide was selected due to its higher solubility and slower aggregation rate compared to the full-length Aβ 1–42. Aggregation experiments were conducted using the Thioflavin T (ThT) fluorescence assay [44], which is a standard method to monitor amyloid aggregation. ThT binds specifically to amyloid fibrils, causing an increase in fluorescence intensity. If a complex inhibits peptide aggregation, the observed fluorescence increase will be lower compared to the reference, indicating inhibition.

In this experiment, the amyloid peptide was first dissolved in 1,1,1,3,3,3-hexafluoro-2-propanol (HFIP) to facilitate its unfolding. A 0.2 mM solution of the peptide was then prepared in 10 mM PBS (containing 100 mM NaCl, pH 7.4). To this solution, equimolar amounts of ThT (prepared in the same buffer) and the complex (dissolved in methanol) were added.

**Figure 7:**
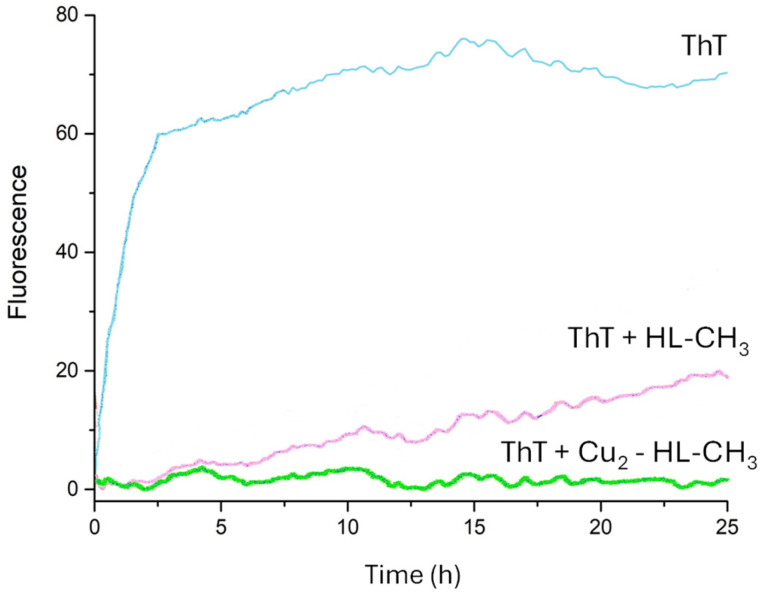
Monitoring of ThT fluorescence resulting from its interaction with β-amyloid aggregates. [ThT] = 10 μM; [β-amyloid 1–40] = 10 μM; [complex] = 10 μM.

As shown in Figure 7, ThT fluorescence varied significantly across the samples. Compared to the peptide alone, the free ligand showed moderate inhibition of the aggregation, likely due to hydrophobic interactions with the amyloid peptide. Remarkably, the dinuclear copper complex exhibited significant anti-aggregation activity across all time points tested.

### 2.6. Peptidic Functionalization of Ligands

Due to this intriguing behavior, introducing targeting functionality may be a promising strategy. Two of the three ligands synthesized in this work contain functionalizable groups, allowing for a wide range of potential modifications. Among these, modifications that enhance solubility or confer amyloid recognition properties are particularly valuable.

**Scheme 5:**
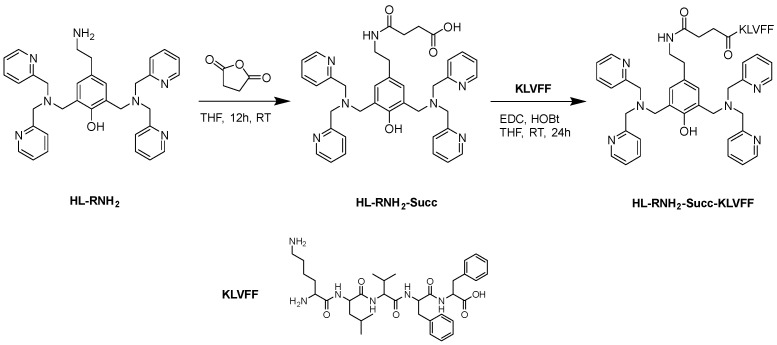
Coupling of the succinic bridged ligand and the peptide KLVFF.

For the latter, we conducted a preliminary functionalization experiment using the anti-aggregation peptide KLVFF [45]. This peptide was conjugated to the ligand HL-RNH_2_ after derivatization with succinic anhydride, which introduced a short spacer between the ligand and peptide (Scheme 5).

For the final step, the formation of the amide bond between the -COOH of the succinyl and the -NH_2_ of the peptide was achieved by using EDC (N-(3-dimethylaminopropyl)-N′-ethylcarbodiimide) and HOBt (1-hydroxybenzotriazole) as coupling agents. Their combination forms the active ester, which reacts with the amino group of the peptide to give the product.

This synthetic procedure proved to be successful, as evidenced by the ESI-MS(+) spectrum (Figure S35). Here, the peak for the product is visible at 1293.69 *m*/*z* (calc. 1293.72 *m*/*z*), while the peak at 652.39 *m*/*z* refers to the unreacted KLVFF (calc. 652.85 *m*/*z*).

Although a purification setup is required (such as HPLC, which is typically the preferred method for peptide purification) and despite selectivity challenges due to free -NH_2_ groups in the lysine side chains of the peptide sequence used here, this strategy illustrates the potential for further tailoring these complexes for advanced Aβ targeting. Selectivity can be improved by incorporating sidechain-protected lysine residues in the synthesis of KLVFF, ensuring stability against cleavage reactions during coupling.

## 3. Materials and Methods

### 3.1. Reagents

Commercially available reagents and solvents were used, provided by Merck (Darmstadt, Germany), with no further purifications. β-Amyloid (1–40) was purchased from GenScript (Leiden, the Netherlands). MilliQ-deionized water (Millipore, Molsheim, France) was used for buffer solutions and for spectroscopic measurements.

### 3.2. Instrumentation

UV–Vis spectra were recorded with spectrometers Varian Cary 50, Cary 100, and Cary 5000 (Varian, Palo Alto, CA, USA), using a standard (3 mL) or reduced (1 mL) volume quartz cuvette with 1 cm of optical path.

^1^H-NMR spectra were obtained with Bruker 200, Bruker AC300, or Bruker Advance DRX 400 MHz spectrometers (Rheinstetten, Germany). The chemical shifts were assigned in accordance with Si(CH_3_)_4_ (*δ* ^1^H-NMR = 0 ppm). Multiplicity is described as s: singlet, d: doublet, t: triplet, td: triplet of doublets, m: multiplet.

FT-IR spectra were measured, preparing KBr pellets, with a Nicolet 5700-Thermo Electron Corporation instrument (Hemel Hempstead, UK). Throughout the vibrational bands assignment, the following symbols were used: w: weak signal, m: medium signal, s: strong signal, b: broad signal.

ESI-MS spectra were collected using an Agilent Technologies MSD SL Trap spectrometer (Waldbronn, Germany) with an ESI source. Samples were dissolved in CH_3_CN, CH_3_CN/H^+^ (0.1% *v*/*v* HCOOH), MeOH, MeOH/H^+^ (0.1% *v*/*v* HCOOH), or H_2_O and injected into the ion source at a flow rate of 0.05 mL/min. For electrospray ionization, the drying gas (N_2_) was heated at 325 °C.

CHN elemental analyses were performed in duplicate by a Thermo Fisher Flash 2000 instrument (Milan, Italy).

### 3.3. Ligands and Complexes Spectra

UV–Vis spectra of ligands and complexes were recorded in a quartz cuvette with 1 cm of optical path. For the ligands, a solution of 0.1 mM in MeOH/H_2_O (5% *v*/*v*) was used. For the complexes, solutions from 0.1 mM to 1.5 mM (to highlight d-d bands) were made in deionized water.

### 3.4. Study of the Complexation Through UV–Vis Spectroscopy

For the determination of the binding constant between metals and ligands, a solution of the latter was titrated with 0.2 eq of metal for each addition. The titrating solutions were 10 mM and 100 μM for Cu(ClO_4_)_2_ versus 0.1 mM and 1 μM for the ligands in 5% *v*/*v* MeOH/BBS (50 mM, pH 7.4), respectively. For the other metals (Mn^2+^, Fe^2+^, and Zn^2+^), the solutions were 10 mM for the relative perchlorate salt and 0.1 mM for the ligand (HL-CH_3_), always in 5% *v*/*v* MeOH/BBS (50 mM, pH 7.4). The measurements were collected in a quartz cuvette, with 1 cm of optical path, filled with 2.5 mL of the ligand solution, and aliquots of 5 μL of the metal solution were added (0.2 eq of M^2+^ for each). The absorbance variation was monitored from 200 nm to 800 nm at 25 °C.

### 3.5. Study of the Complexation Through Potentiometry

Potentiometric measurements were carried out using the Metrohm pH-meter system (Herisau, Switzerland) with a Metrohm semi-micro combination electrode at 25 °C, calibrated in a hydrogen ion concentration using HCl [46]. The ligands were dissolved in DMSO (5% *v*/*v*/H_2_O (HCl/KCl)) in order to obtain a concentration of 1 mM. The titrant (KOH 0.1M) was added with a micrometric syringe at 298 K in the pH range of 2.5–11.5 in an argon atmosphere. The stability constants and stoichiometry of the complexes were calculated using the titration curves and the SUPERQUAD and HYPERQUAD [47,48].

Cyclic Voltammetry experiments were performed using a BAS Cell C3 EC-epsilon potentiostat (Cambridge, UK) with the three-electrode cell composed of a glassy carbon electrode (WE, 3 mm diameter, geometric surface 7 mm^2^), a platinum wire electrode (CE), and an Ag/AgCl electrode (RE, 3 M KCl). The cyclic voltammograms were collected in PBS (10 mM, 150 mM NaCl, pH 7.8) with 0.25 mM complex. The typical three-electrode cell was composed of a working glassy carbon electrode, a counter electrode (Pt), and a reference Ag/AgCl electrode (3 M KCl). The curves were measured between −600 and +400 mV with different scan rates (50 mV/s, 100 mV/s, 200 mV/s, 400 mV/s, and 800 mV/s).

Catalytic measurements were carried out in a Varian Cary 100 instrument, with a 3 mL quartz cuvette (1 cm of optical path) for SOD. For the peroxidase-like activity, Varian Cary 50 instrument and 3 mL quartz cuvettes (1 cm optical path) were used. To monitor the CAT-like activity, a 25 mL vial was used as a reactor, connected to a pressure transducer and a septum for the injection.

Models of complexes were obtained by Avogadro, an open-source molecular builder and visualization tool (Version 1.2.0. http://avogadro.cc/) [23].

## 4. Conclusions

This work focused on three previously known dinuclear metal complexes, which were resynthesized and investigated to uncover their innovative multitarget potential. These complexes exhibit a combination of metal-chelating and antioxidant properties, demonstrating dual SOD- and CAT-like activity in aqueous media. Such attributes position them as promising candidates for the treatment of Alzheimer’s disease (AD). To evaluate these complexes, the following experiments were performed:

- Chelation ability and stability: The chelation properties and stability of the complexes were evaluated by titrating the ligands with copper(II) solutions and tracking changes through UV–Vis spectroscopy, allowing for the measurement of binding constants between the ligands and Cu^2+^. The highest binding constant was observed for HL-RNH_2_ (>10^12^ M^−2^), highlighting its potential for effective metal sequestration in biological contexts.
- Metal selectivity: To evaluate selectivity, the binding constant of HL-CH_3_ with other metals (Mn^2+^, Fe^2+^, and Zn^2+^) was measured. Results showed a binding preference for copper ions approximately three orders of magnitude higher than for the other ions, suggesting that similar selectivity may apply to the other ligands with minor variations.
- Redox properties: Cyclic voltammetry was used to investigate the redox properties of the complexes, determining their suitability for catalysis within an optimal reduction potential range. Voltammograms confirmed a suitable reduction potential for SOD-like catalysis and indicated a dielectronic process in the Cu^2+^/Cu^+^ reduction, suggesting stability and readiness for redox reactions relevant to antioxidant function.
- SOD-like activity: The complexes’ capacity to dismutate the superoxide radical anion (O_2_^●−^) into oxygen and hydrogen peroxide was measured. Among them, [Cu_2_(L-RNH_2_)(H_2_O)_2_](ClO_4_)_3_ showed the most efficient superoxide dismutation activity, with log(k_SOD_) = 6.59.
- CAT-like activity: The complexes’ capability to remove hydrogen peroxide was evaluated by monitoring the production of molecular oxygen from H_2_O_2_ decomposition. The complex [Cu_2_(L-RNH_2_)(H_2_O)_2_](ClO_4_)_3_ exhibited the highest initial activity; however, this decreased over time, likely due to oxidative degradation of the ligand.
- Peroxidase-like activity: To assess residual pro-oxidant behavior, peroxidase-like activity assays were conducted using OPD as an oxidizable substrate. [Cu_2_(L-RNH_2_)(H_2_O)_2_](ClO_4_)_3_ showed the highest activity, though significantly lower than free copper(II) ions.
- Anti-aggregation effect: The anti-amyloid aggregation capability of [Cu_2_(L-CH_3_)(H_2_O)_2_](ClO_4_)_3_ was evaluated by the ThT test and showed complete inhibition of Aβ 1–40 aggregation.

In summary, the dinuclear copper complexes with N_6_O donor ligands exhibit multi-faceted therapeutic potential against Alzheimer’s disease. Their selective binding to copper, strong SOD- and CAT-like activities, moderated peroxidase activity, and potent anti-aggregation effects make them promising candidates for further exploration in AD-related applications. These initial findings lay the groundwork for future optimization, including structural modifications to enhance stability and functionalization to improve targeting efficiency, providing a suitable platform for multifunctional therapeutic agents in AD management.

## Figures and Tables

**Table 1:** Overall (logβ_i_) and stepwise (logK_i_) protonation constants of the ligand (*L* = L-CH_3_) were obtained from the potentiometric titration.

Equilibrium Reaction	logβ_i_	Equilibrium Reaction	logK_a_
*L*^−^ + H^+^ ⇌ H*L*	9.75 ± 0.01	*L* + H^+^ ⇌ H*L*	9.75
*L*^−^ + 2H^+^ ⇌ [H_2_*L*]^+^	16.57 ± 0.01	H*L* + H^+^ ⇌ [H_2_*L*]^+^	6.82
*L*^−^ + 3H^+^ ⇌ [H_3_*L*]^2+^	21.56 ± 0.01	[H_2_*L*]^+^ + H^+^ ⇌ [H_3_*L*]^2+^	4.99
*L*^−^ + 4H^+^ ⇌ [H_4_*L*]^3+^	25.27 ± 0.01	[H_3_*L*]^2+^ + H^+^ ⇌ [H_4_*L*]^3+^	3.71
*L*^−^ + 5H^+^ ⇌ [H_5_*L*]^4+^	27.83 ± 0.02	[H_4_*L*]^3+^ + H^+^ ⇌ [H_5_*L*]^4+^	2.56
*L*^−^ + 6H^+^ ⇌ [H_6_*L*]^5+^	29.60 ± 0.11	[H_5_*L*]^4+^ + H^+^ ⇌ [H_6_*L*]^5+^	1.77

**Table 2:** The overall (logβi) and stepwise (logK_i_) protonation constants of the in situ-formed complex of Cu(II) with *L* = L-CH_3_, as obtained from potentiometric titration.

Species	logβ_i_	logK_i_
[CuH_3_*L*]^4+^	26.17 ± 0.03	4.00
[CuH_2_*L*]^3+^	22.17 ± 0.03	5.15
[CuH*L*]^2+^	17.02 ± 0.03	6.82
[Cu*L*]^+^	10.20 ± 0.03	9.58
[CuH_−1_*L*]	0.62 ± 0.04	-
[Cu_2_H_2_*L*]^5+^	26.21 ± 0.03	4.83
[Cu_2_H*L*]^4+^	21.38 ± 0.03	5.88
[Cu_2_*L*]^3+^	15.50 ± 0.03	9.26
[Cu_2_H_−1_*L*]^2+^	6.24 ± 0.04	-

**Table 3:** Summary of the binding constants for the three ligands and copper, calculated with the non-linear regression method.

Complexes	K_bind_ (M^−2^)	log(K_bind_)
[Cu_2_(L-CH_3_)(H_2_O)_2_](ClO_4_)_3_	1.96 × 10^11^	11.3
[Cu_2_(L-RNH_2_)(H_2_O)_2_](ClO_4_)_3_	1.37 × 10^12^	12.1
[Cu_2_(L-RCOOH)(H_2_O)_2_](ClO_4_)_3_	5.16 × 10^11^	11.7

**Table 4:** Summary of the binding constants for Mn(II), Fe(II), Zn(II), and HL-CH_3_ calculated with the non-linear regression method.

Metal	K_bind_ (M^−2^)	log(K_bind_)
Mn(II)	6.12 × 10^7^	7.78
Fe(II)	1.09 × 10^8^	8.03
Zn(II)	2.49 × 10^8^	8.39

**Table 5:** Summary of values of E_pc_, E_pa_, E_1/2_, |ΔE|, and |i_a_/i_c_| obtained from the voltammograms.

Complexes	E_pc_ (V)	E_pa_ (V)	E_1/2_ (V)	|ΔE| (V)	|i_a_/i_c_|
[Cu_2_(L-CH_3_)(H_2_O)_2_](ClO_4_)_3_	−0.305	−0.052	−0.179	0.253	0.78
[Cu_2_(L-RNH_2_)(H_2_O)_2_](ClO_4_)_3_	−0.297	−0.123	−0.210	0.174	0.63
[Cu_2_(L-RCOOH)(H_2_O)_2_](ClO_4_)_3_	−0.360	−0.135	−0.248	0.225	0.99

**Table 6:** IC_50_ and log(k_SOD_) values for the three synthesized complexes.

Complexes	IC_50_ (μM)	log(k_SOD_)
[Cu_2_(L-CH_3_)(H_2_O)_2_](ClO_4_)_3_	0.75	6.54
[Cu_2_(L-RNH_2_)(H_2_O)_2_](ClO_4_)_3_	0.66	6.59
[Cu_2_(L-RCOOH)(H_2_O)_2_](ClO_4_)_3_	0.72	6.56

**Table 7:** List of R_0_, Rmax, TOF, TON, k_CAT_, and n(O_2_).

Complexes	R_0_ (µM O_2_ s^−1^)	Rmax (µM O_2_ s^−1^)	TOF (s^−1^)	TON	k_CAT_ (M^−1^ s^−1^)	n(O_2_) (µmol)
[Cu_2_(L-CH_3_)(H_2_O)_2_](ClO_4_)_3_	0.51	0.75	0.0026	35	0.28	85
[Cu_2_(L-RNH_2_)(H_2_O)_2_](ClO_4_)_3_	0.81	0.92	0.0040	38	0.88	90
[Cu_2_(L-RCOOH)(H_2_O)_2_](ClO_4_)_3_	0.18	0.74	0.0009	43	0.30	103

Conditions: [complex] = 200 µM, [H_2_O_2_] = 22.5 mM in BBS (pH 7.8) and T = 25 °C. R_0_ = calculated at conversion <10%. Rmax = calculated at the maximum slope of the kinetic curves.

## Data Availability

The data are available on request from the corresponding authors.

## Supplementary Materials

The following supporting information can be downloaded at: https://www.mdpi.com/article/10.3390/molecules29235708/s1, synthesis and spectroscopic measurements. References [49,50,51,52,53] are cited in the Supplementary Materials.
