# Supplementary material for: Chelating Properties of N6O-Donors Toward Cu(II) Ions: Speciation in Aqueous Environments and Catalytic Activity of the Dinuclear Complexes
Source: Molecules. 2024 Dec 3;29(23):5708. doi: 10.3390/molecules29235708 (PMC11643690; doi:10.3390/molecules29235708)
Supplement: Supplementary file 1 [file molecules-29-05708-s001.zip › molecules-3315350-supplementary.pdf]

Chelating Properties of N_6O-Donors Toward Cu(II) Ions: Speciation in Aqueous Environments and Catalytic Activity of the Dinuclear Complexes

Andrea Cendron, Martina Chianese, Kamil Zarzycki, Paolo Ruzza, Claudia Honisch, Justyna Brasuń and Mauro Carraro

1. Materials

Commercially available reagents and solvents were used, provided by Merck and were used with no further purifications. MilliQ-deionized water (Millipore) was used for buffer solutions and for spectroscopic measurements.

2. Syntheses

Synthesis of DPA (di-(2-picolyl) amine)

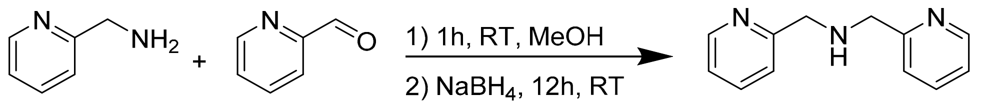

DPA

DPA synthesis was carried out following the procedure described in literature by *Aðalsteinsson et al.*[1]. Pyridine-2-carboxaldehyde (1.07 g, 10 mmol) dissolved in 5 mL of MeOH, was added dropwise to 2-(aminomethyl)pyridine (1.08 g, 10 mmol) in 5 mL of methanol. The solution was stirred for 1h at room temperature, then 1.05 g (28 mmol) of $NaBH_4$ were added at 0 °C. The solution turned yellow immediately and a temporary formation of foam was observed. The reaction mixture was stirred for 12h at RT, changing the color to dark orange. The solvent was then removed under reduced pressure and the brown residue was dissolved in 30 mL of H_2O. The product was extracted with CH_2Cl_2 (4 x 30 mL) and the organic phase was dried with Na_2SO_4. The mixture was filtered, and the solvent was removed under vacuum to obtain a dark brown oil (1.54 g, 7.72 mmol, yield 77%).

[1] Aðalsteinsson, H. M.; Lima, F. A.; Galuppo, C.; Abbehausen, C.; *Dalton Trans.,* **2020**, 49, 16143–16153.

¹H-NMR (300 MHz, $CDCl_3$) δ (ppm): 8.60 (d, J=4.5 Hz, 2H), 7.68 (td, J=7.7, 1.8 Hz, 2H), 7.39 (d, J=7.7 Hz, 2H), 7.16-7.23 (m, 2H), 4.02 (s, 4H), 1.37 (s, 1H, -NH)

ESI-MS(+) (*m/z*) in CH_3CN/H^+: 200.06 $[DPA+H]^+$ (calc. 200.12).

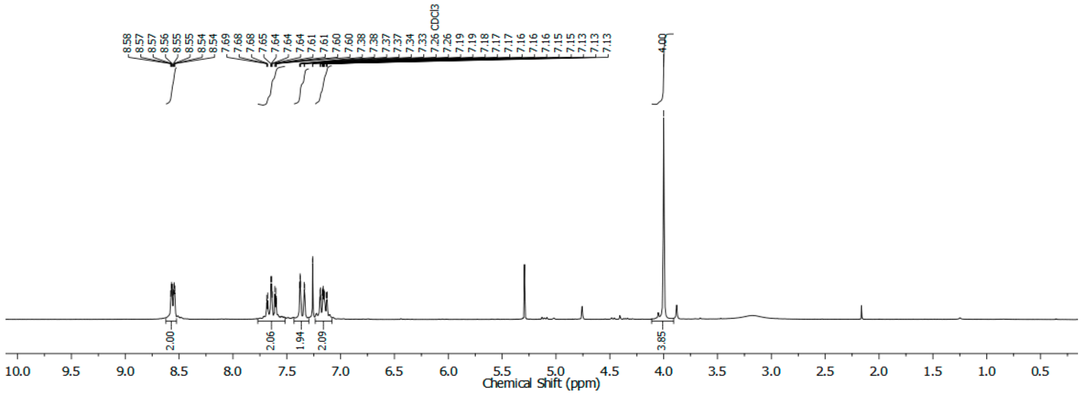

Figure S1: *¹H-NMR spectrum (300 MHz, $CDCl_3$) of DPA.*

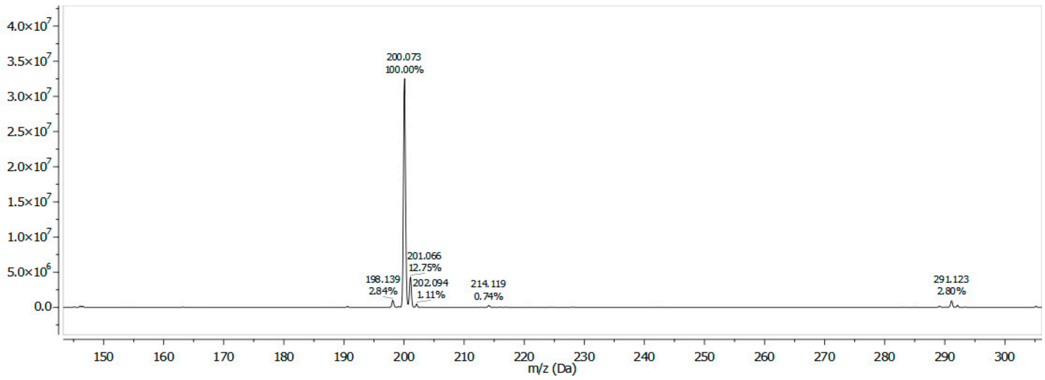

Figure S2: *ESI-MS(+) (m/z) in CH_3CN/H^+ of DPA.*

Synthesis of HL-CH_3

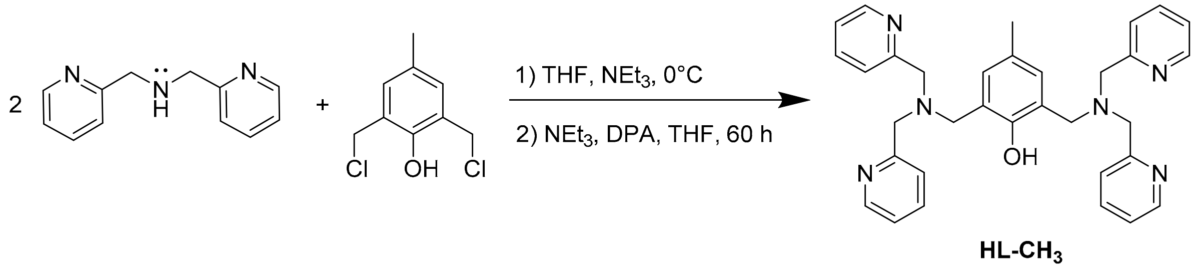

The synthesis of HL-CH_3 (2,6-bis[(bis(2-pyridylmethyl)amino)methyl]-4-methylphenol) was performed following the procedure described by *Torelli et al.*[2]. Under nitrogen atmosphere a solution of DPA (0.99 g, 4.97 mmol) and NEt_3 (1.50 mL, 10.8 mmol) in 3 mL of anhydrous THF was added dropwise to a solution of 2,6-bis(chloromethyl)-4-methylphenol (0.53 g, 2.60 mmol) in 5 mL of anhydrous THF at 0 °C. A brown mixture with a yellow precipitate formed, which was left at room temperature for 60 h. After that, the solution was filtered (to remove the precipitate containing the triethylammonium salt) and the filtrate was reduced under in vacuum, obtaining a brown oil. Subsequently, 30 mL of DCM were added to the oil. This solution was washed with brine (2 x 10 mL), dehydrated with Na_2SO_4, and then the solvent was removed under vacuum. A brown sticky oil was obtained (0.77 g, 1.45 mmol, yield 60%).

^{1}H-NMR (300 MHz, $CDCl_3$) δ (ppm): 8.52 (d, J=3.59, 4H), 7.60 (dt, J=7.40, 1.73 Hz, 4H), 7.49 (d, J=6.72, 4H), 7.13 (m, 4H), 6.99 (s, 2H) 3.86 (s, 8H), 3.77 (s, 4H), 1.19 (s, 3H).

ESI-MS(+) (*m/z*) in CH_3CN/H^+: 531.28 $[HL\text{-}CH_3+H]^+$ (calc. 531.29).

FT-IR (KBr): ν (cm^{-1}) = 3448 (b), 3209 (b, w), 3049 (w), 3000 (w), 2919 (m), 2807 (m), 1681 (w), 1634 (w), 1591 (s), 1569 (s), 1472 (s), 1430 (s), 1470 (s), 1370 (m), 1294 (m), 1263 (s), 1219 (m), 1149 (m), 1145 (m), 1049 (m), 997 (m), 859 (m), 757 (s), 611 (w), 502 (w), 469 (w).

---

2 Torelli, S.; Belle, C.; Gautier-Luneau, I.; Pierre, J. L.; Saint-Aman, E.; Latour, J. M.; Le Pape, L.; Luneau, D.; *Inorg. Chem.*, **2000**, 39, 3526–3536.

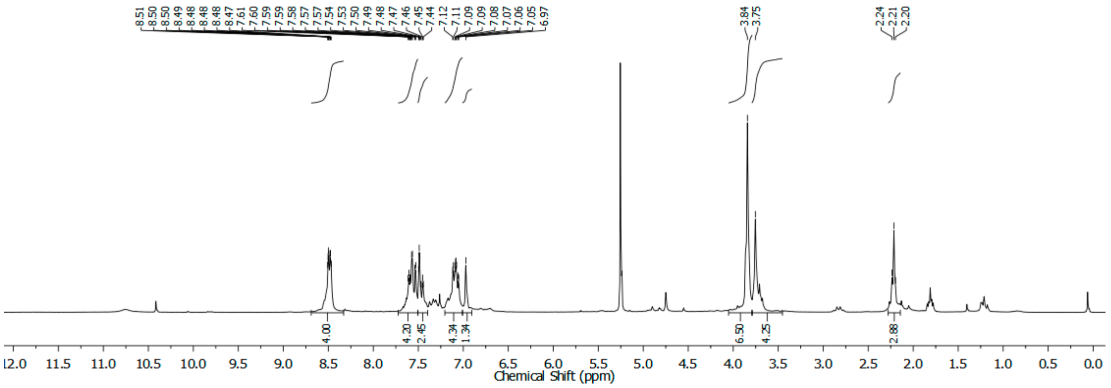

Figure S3: *^{1}H-NMR spectrum (300 MHz, $CDCl_3$) of HL-CH_3.*

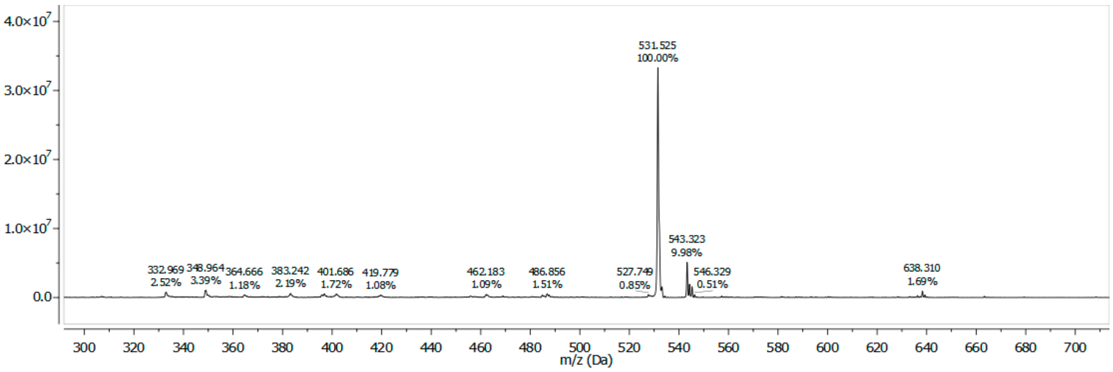

Figure S4: *ESI-MS(+) (m/z) in CH_3CN/H^+ of HL-CH_3.*

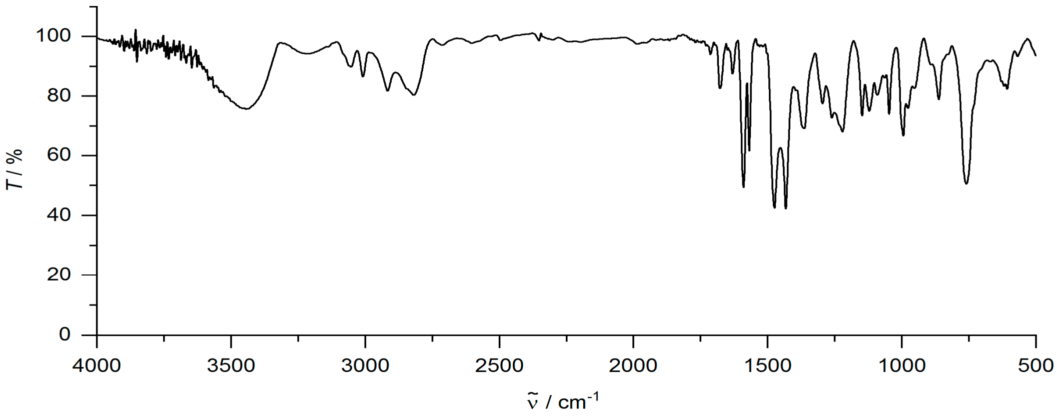

Figure S1: *FT-IR (KBr) spectrum of HL-CH_3. Characteristic bands: 3400 cm^{-1} phenolic O-H stretching; 3050-3100 cm^{-1} aromatic C-H stretching; 2700-2950 cm^{-1}, methylene and methyl C-H stretching; 1434-1592 cm^{-1} (aromatic C=C and C=N stretching); 1365 cm^{-1} (C-OH bending); 1261 cm^{-1} (phenolic C-OH stretching); 802 cm^{-1} (aromatic C-H bending).*

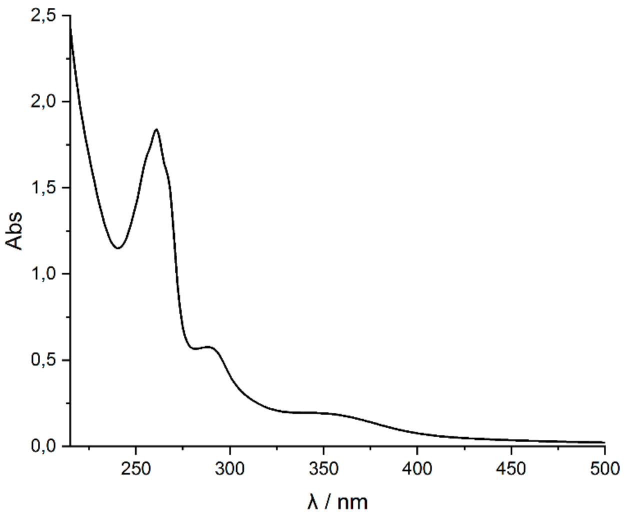

Figure S6: *UV-vis spectrum of the ligand HL-CH$_3$ in MeOH (5% v/v)/H$_2$O, 0.1 mM. π → π* transition of the aromatic groups can be observed at 261 nm, together two weaker bands at 290 nm and 351 nm.*

Synthesis of HL-RNH$_2$

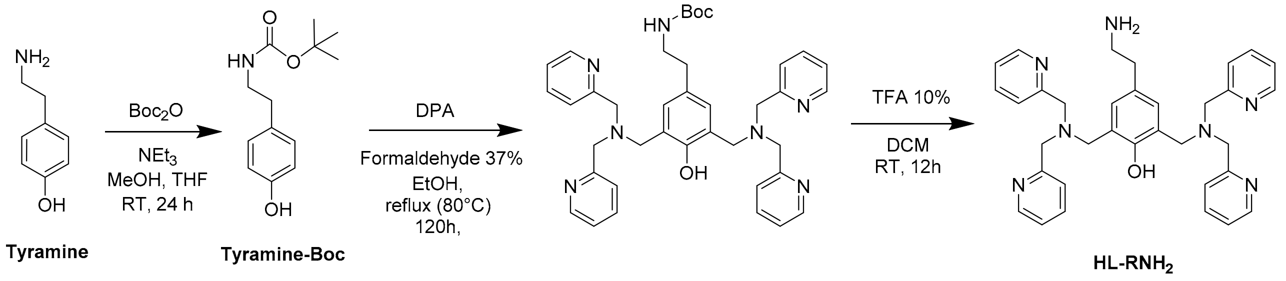

This synthesis was carried out according to *Moffat et al.*[3]. The first step is the formation of the Boc-protected tyramine derivative. Tyramine (1.11 g, 8.11 mmol) was stirred in 50 mL of THF and 10 mL of MeOH at 0 °C. Triethylamine (1.2 mL, 8.44 mmol) was then added. Boc$_2$O (1.79 g, 8.20 mmol) was dissolved in a small amount of MeOH and added dropwise to the previous solution under stirring. The mixture was then allowed to rise slowly to room temperature and then left under stirring for 24h. The solvent was then removed under reduced pressure. The resulting pale oil was taken up with ethyl acetate (30 mL) and washed with water (4 x 10 mL). The organic layer was then dried with Na$_2$SO$_4$ overnight, filtered and the solvent removed. The resulting oil was purified by silica column chromatography with 2:1 hexane/ethyl acetate as eluent (Rf. 0.3) to yield the desired product as a light orange solid after high vacuum treatment (1.31 g, 5.50 mmol, yield 68%).

[3] Moffat, C. D.; Weiss, D. J.; Shivalingam, A.; White, A. J. P.; Salaun, P.; Vilar, R.; *Chem. - A Eur. J.*, **2014**, 20, 17168–17177

^{1}**H-NMR** (200 MHz, $CDCl_3$) δ (ppm): 7.95 (s, 1H, N*H*), 6.93 (d, *J* = 8.4 Hz, 2H, Ar*H*), 6.75 (d, *J* = 8.4 Hz, 2H), 5.01 (s, 1H, O*H*), 3.30 (m, 2H, CH_2), 2.62 (t, *J* = 7.0 Hz, 2H, CH_2), 1.38 (s, 9H, Boc).

ESI-MS(+) (*m/z*) in MeOH/H^+: 260.02 [Tyramine-Boc+Na]$^+$ (calc. 260.13).

The second step is a *Mannich* reaction, where 37% formaldehyde (0.25 mL, 3.4 mmol) in 3 mL of EtOH was stirred at room temperature. DPA (0.50 g, 2.5 mmol) was dissolved in 1 mL of EtOH and then added dropwise to the previous solution, and the mixture was stirred under reflux (80 °C) overnight. Tyramine-Boc (0.29 g, 1.24 mmol) solubilized in 1.5 mL of EtOH was then added and the mixture was left under reflux for 6 more days. The reaction was then allowed to cool down and the solvent was removed. The residue was dissolved in 6 mL of ethyl acetate, washed with 3 x 3 mL of water, dried over Na_2SO_4, filtered and the solvent was removed under reduced pressure. A brown oil was recovered (0.69 g, 1.04 mmol, 85%). This oil was crystallized with 2:1 pentane/ethyl acetate, with the slow interface crystallization technique: the product was dissolved in the least amount of hot ethyl acetate and then pentane was added slowly along the walls of the flask, being careful that two layers formed. The mixture was then left in the fridge at 4 °C for 2 days, finally recovering white crystals (0.21 g, 0.32 mmol, yield 26%).

^{1}**H-NMR** (300 MHz, $CDCl_3$) δ 8.52 (d, *J* = 4.9 Hz, 4H), 7.59 (td, *J* = 7.6, 1.8 Hz, 4H), 7.47 (d, *J* = 7.8 Hz, 4H), 7.11 (t, *J* = 7.3, 5.2 Hz, 4H), 7.01 (s, 2H), 3.86 (s, 8H), 3.78 (s, 4H), 3.31 (t, *J* = 7.0 Hz, 2H), 2.68 (t, *J* = 7.0 Hz, 2H), 1.40 (s, 9H Boc-group).

ESI-MS(+) (*m/z*) in CH_3CN/H^+: 660.28 [HL-RNH-Boc+H]$^+$ (calc. 660.36).

The last step is the deprotection of HL-RNH-Boc (1.03, 1.55 mmol) in DCM (25 mL) and was carried out adding an excess of TFA (4 mL). The mixture was stirred at room temperature overnight. The solvent was then removed, the residue dissolved again in DCM (20 mL) and neutralized with a saturated aqueous solution of $NaHCO_3$. The organic part was then dried with Na_2SO_4 and the solvent removed under reduced pressure, obtaining HL-RNH_2 as a sticky brown oil (0.79 g, 1.41 mmol, yield

90%). The oil was then dissolved in DCM and triturated with hexane, obtaining a light brown solid after removing the solvent. The solid was very hygroscopic, tending to reform the oil.

^{1}H-NMR (300 MHz, $CDCl_3$) δ (ppm): 8.49 (d, 4H), 7.57 (td, J = 7.7, 1.8 Hz, 4H), 7.42 (d, J = 7.8 Hz, 4H), 7.15 – 7.04 (m, 4H), 6.95 (s, 2H), 3.83 (s, 8H), 3.72 (s, 4H), 3.04 (t, J = 6.9 Hz, 2H), 2.76 (t, J = 7.2 Hz, 2H).

ESI-MS(+) (*m/z*) in CH_3CN/H^+: 560.23 $[HL\text{-}RNH_2+H]^+$ (calc. 560.31), 582.19 $[HL\text{-}RNH_2+Na]^+$ (calc. 582.30).

FT-IR (KBr): ν (cm^{-1}) = 3454 (b), 3066 (w), 3014 (w), 2925 (m), 2827 (m), 1694 (s), 1595 (s), 1571 (s), 1480 (s), 1435 (s), 1373 (m), 1296 (w), 1253 (m), 1202 (s), 1131 (s), 1047 (m), 1003 (s), 876 (m), 834 (m), 767 (s), 722 (m), 624 (w), 506 (w), 405 (m).

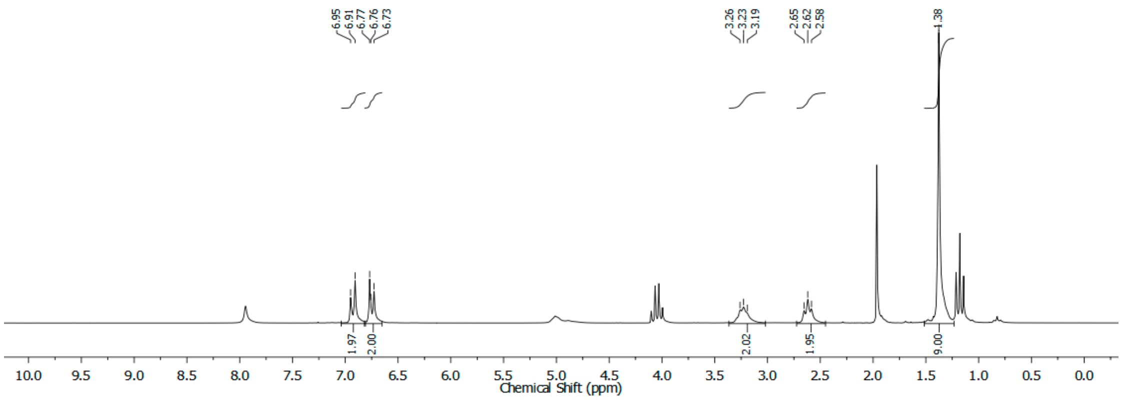

Figure S7: *^{1}H-NMR spectrum (200 MHz, CD_3OD) of Tyramine-Boc.*

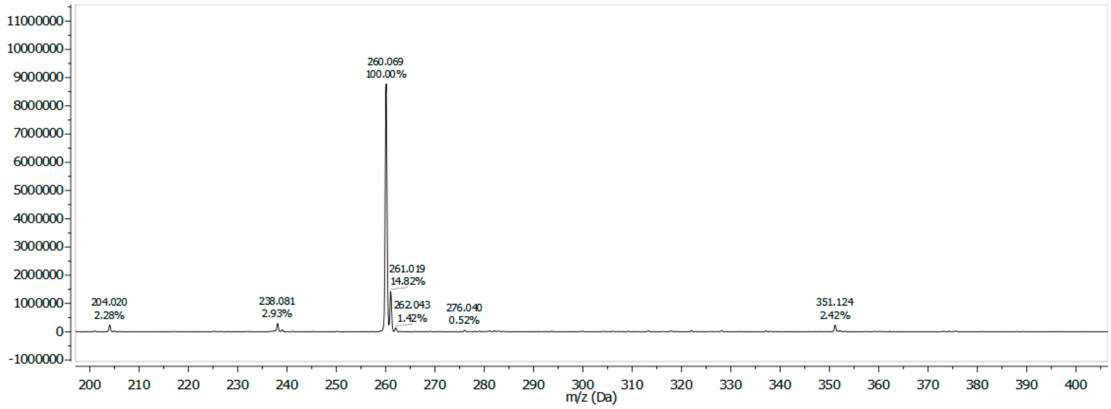

Figure S8: *ESI-MS(+) (m/z) in MeOH/H^+ of Tyramine-Boc.*

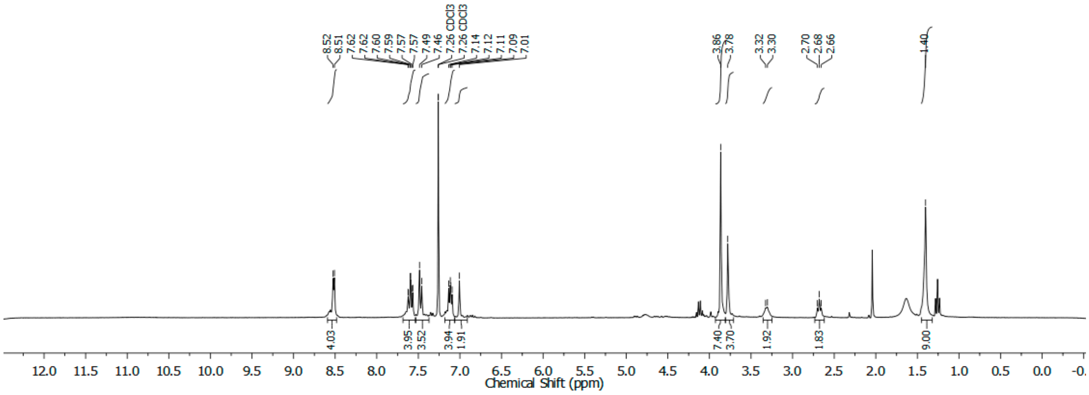

Figure S9: *^{1}H-NMR spectrum (300 MHz, $CDCl_3$) of HL-RNH-Boc.*

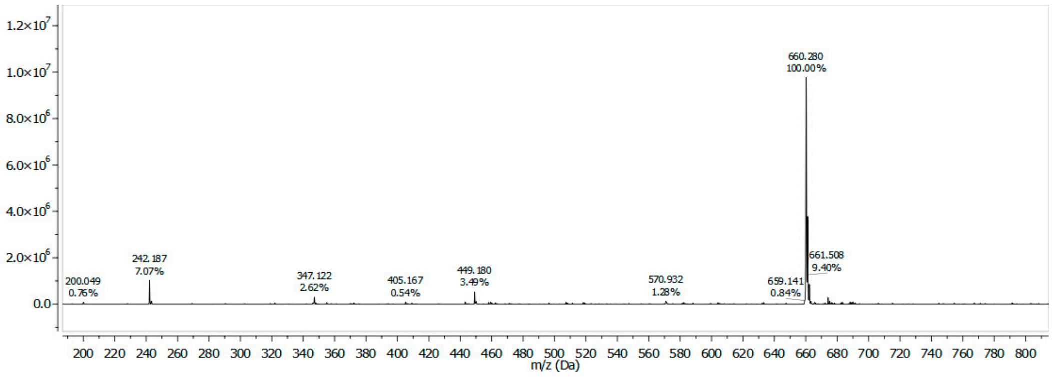

Figure S10: *ESI-MS(+) (m/z) in CH_3CN/H^+ of HL-RNH-BOC.*

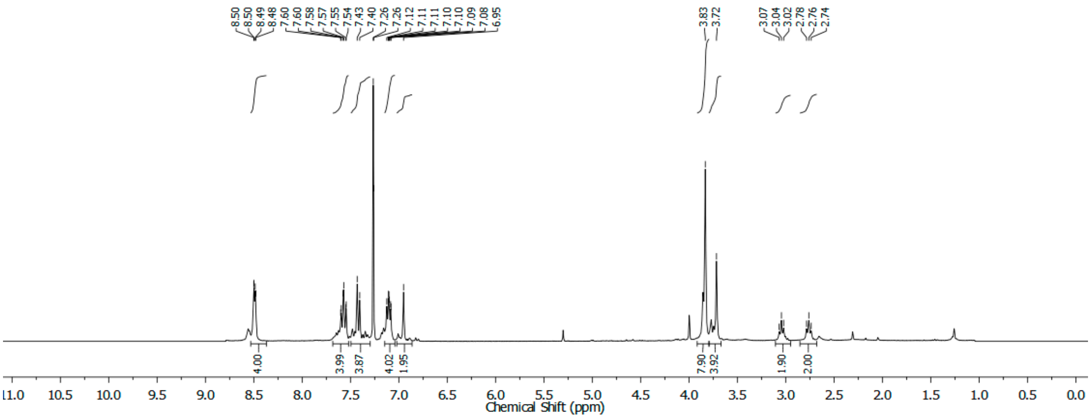

Figure S11: *^{1}H-NMR spectrum (300 MHz, $CDCl_3$) of HL-RNH_2.*

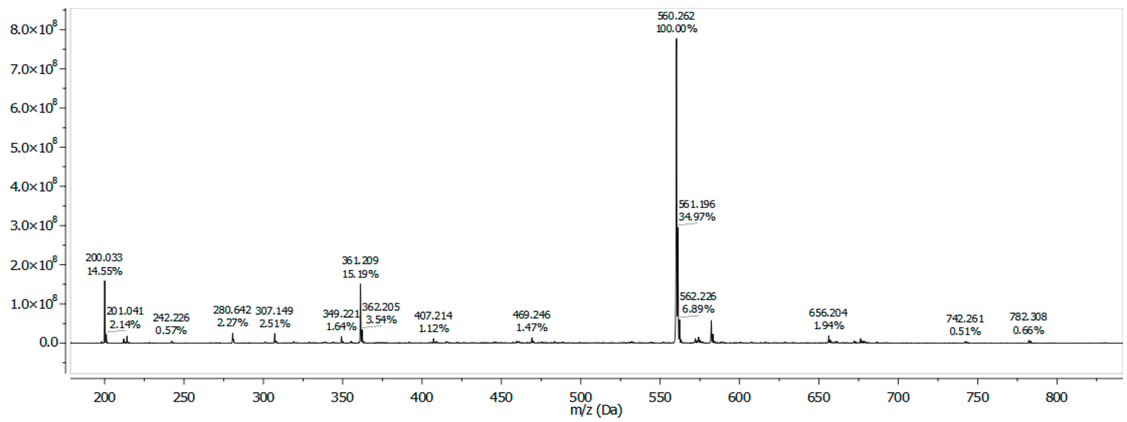

Figure S12: *ESI-MS(+) (m/z) in CH_3CN/H^+ of HL-RNH_2.*

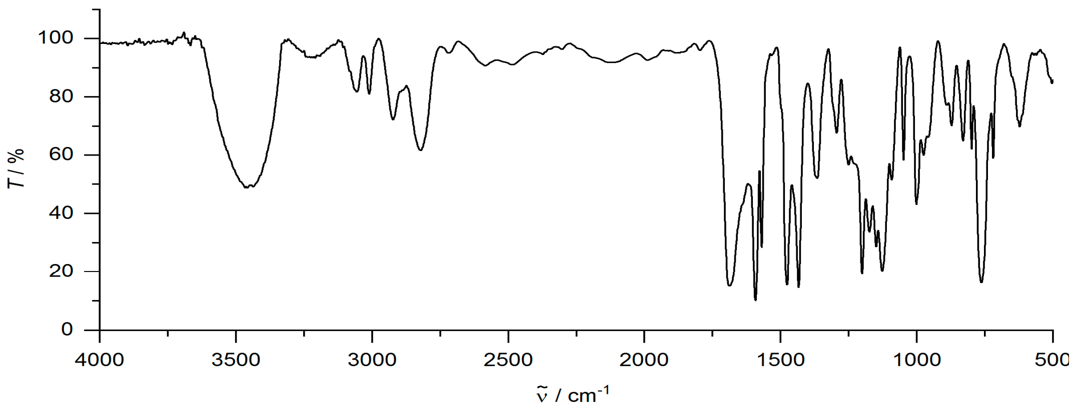

Figure S13: *FT-IR (KBr) spectrum of HL-RNH_2. Characteristic bands: in addition to the bands described in Fig. S5, N-H and O-H stretching give the broad band at 3450 cm^{-1}; stronger bands are also observed for the methylene C-H stretching at 3000 cm^{-1}.*

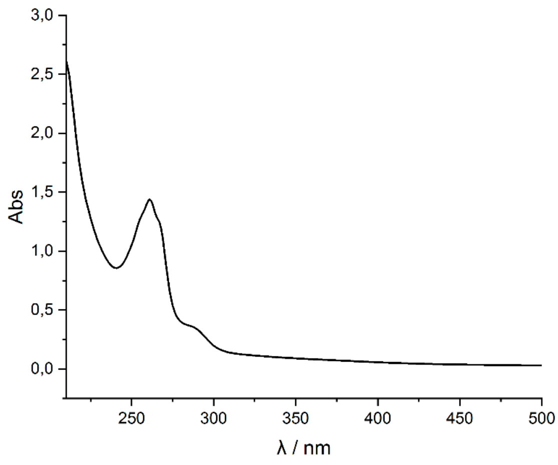

Figure S14: *UV-vis spectrum of the ligand HL-RNH_2 in MeOH (5% v/v)/H_2O, 0.1 mM. The transition $\pi \rightarrow \pi^*$ of the aromatic groups observed at 261 nm, together with a smaller band (shouder) at 288 nm.*

Synthesis of HL-RCOOH

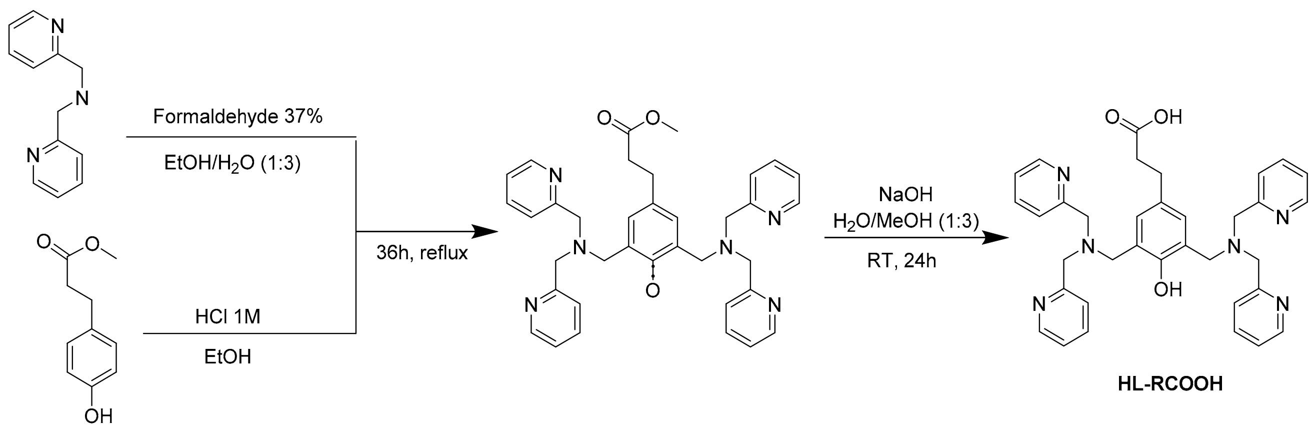

The whole synthesis of this ligand was described by *Oh et al*[4], using the same *Mannich* reaction of the previous one. DPA (2.20 g, 11.0 mmol) and formaldehyde 37% (0.45 mL, 16.1 mmol) were stirred in a solution of EtOH (15 mL) and H_2O (45 mL). (methyl 3-(4-hydroxyphenyl)propionate, 0.93 g, 5.14 mmol) was dissolved in EtOH (20 mL) and acidified with HCl 1N (1 mL) was then added to the former solution. The clear yellow mixture was refluxed for 3 days, with the color changing to red. After neutralization with Na_2CO_3, the intermediate HL-RCOOMe was extracted with DCM (20 mL), and the solvent was removed giving a brown oil (2.58 g, 4.29 mmol, yield 83%).

^{1}**H-NMR** (300 MHz, $CDCl_3$) δ (ppm): 8.50 (d, J=4.05 Hz, 4H), 7.59 (dt, J=7.72, 1.84 Hz, 4H), 7.43 (d, J=7.80, 4H), 7.13 (m, 4H), 7.02 (s, 2H), 3.86 (s, 8H), 3.78 (s, 4H), 3.62 (s, 3H), 2.87 – 2.81 (m, 2H), 2.58 – 2.53 (m, 2H).

ESI-MS(+) (*m/z*) in CH_3CN/H^+: 603.24 [**11**+H]$^+$ (calc. 603.31).

The hydrolysis of ester HL-RCOOMe was achieved dissolving it in MeOH (30 mL) and H_2O (10 mL) and adding an excess of NaOH (0.17 g, 4.35 mmol). The solution was stirred for 24h at RT, then most of the MeOH was removed under reduced pressure. The aqueous phase was washed with DCM (3 x 40 mL) and neutralized with 1 N HCl. The final product was extracted from the aqueous phase with DCM (4 x 20 mL), dried over Na_2SO_4 and the solvent removed in vacuum (1.87 g, 3.18 mmol, yield 74%).

^{1}**H-NMR** (300 MHz, $CDCl_3$) δ (ppm): 8.67 – 8.40 (m, 4H), 7.72 – 7.55 (m, 4H), 7.43 (t, J=7.2, 4H), 7.19 – 7.09 (m, 4H), 7.07 (s, 2H), 3.85 (s, 8H), 3.79 (s, 4H), 2.96 – 2.77 (m, 2H), 2.67 – 2.50 (m, 2H).

ESI-MS(+) (*m/z*) in CH_3CN/H^+: 589.20 [HL-RCOOH+H]$^+$ (calc. 589.29).

4 Oh, D. J.; Kim, K. M.; Ahn, K. H.; *Chem. - An Asian J.*, **2011**, 6, 2034–2039.

FT-IR (KBr): ν (cm^{-1}) = 3451 (b), 3199 (b, w), 3066 (w), 3013 (w), 2926 (m), 2808 (w), 2720 (w), 2588 (w), 1975 (b, w), 1717 (s), 1595 (s), 1571 (s), 1482(s), 1436 (s), 1375 (s), 1295 (s), 1235 (s), 1150 (s), 1122 (s), 1090 (s), 1050 (m), 1004 (s), 977 (s), 874 (m), 767 (s), 637 (m), 474 (w).

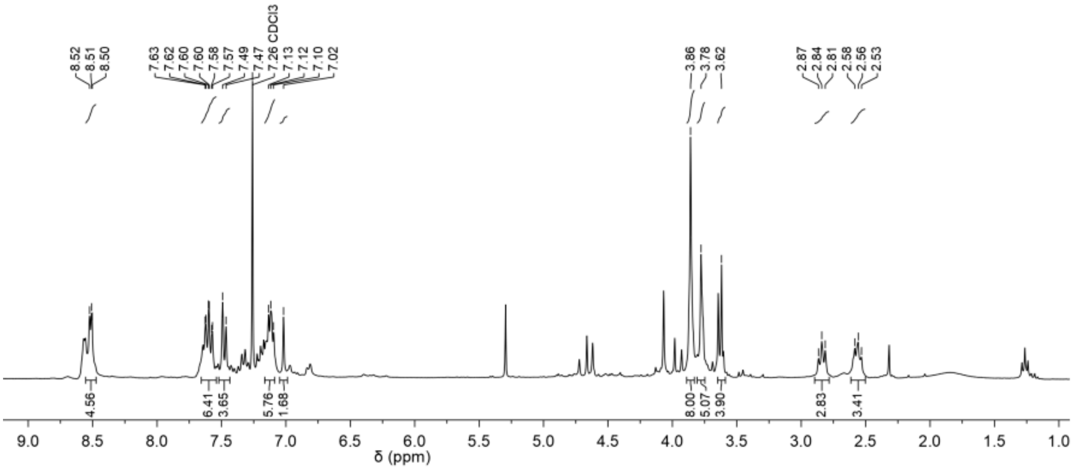

Figure S15*: ¹H-NMR spectrum (300 MHz, CDCl₃) of HL-RCOOCH₃.*

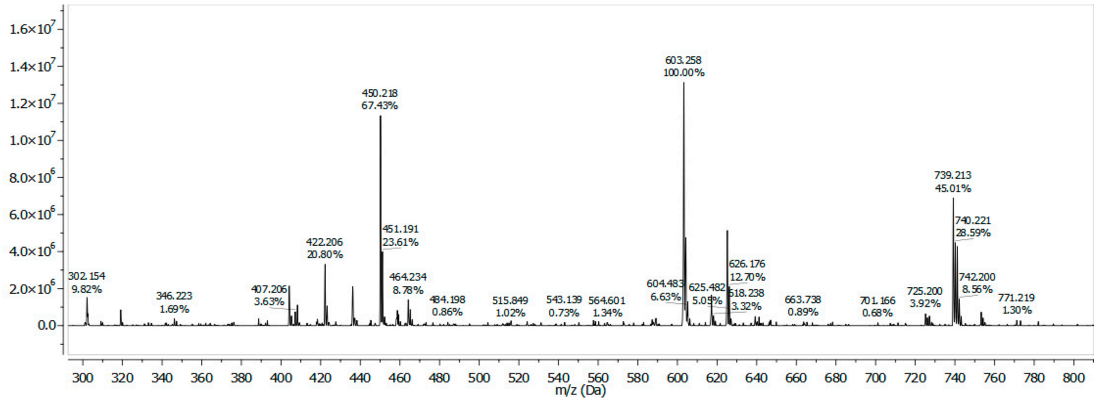

Figure S16*: ESI-MS(+) (m/z) in CH₃CN/H⁺ of HL-RCOOCH₃.*

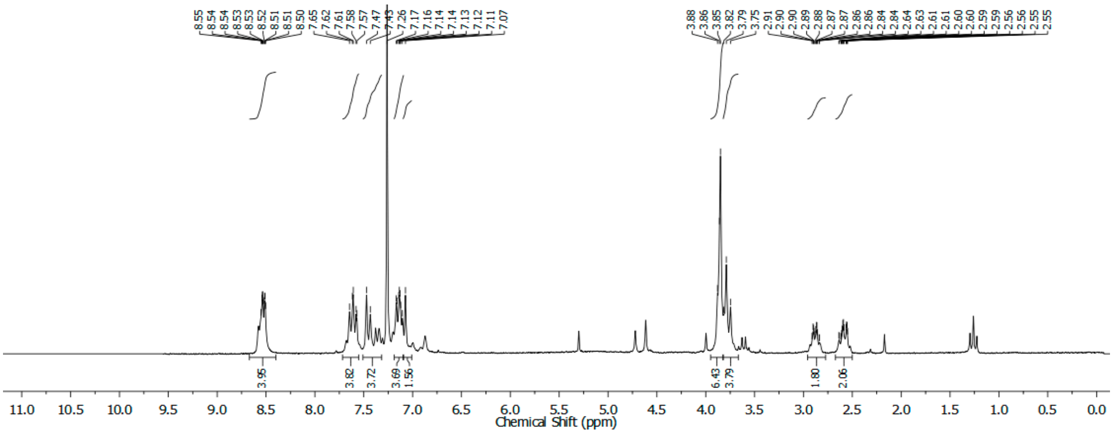

Figure S17: *^{1}H-NMR spectrum (300 MHz, $CDCl_3$) of HL-RCOOH.*

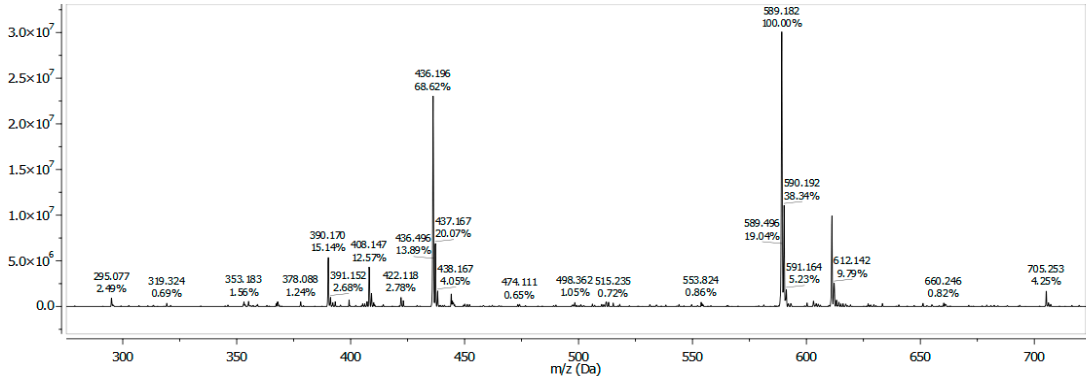

Figure S18: *ESI-MS(+) (m/z) in CH_3CN/H^+ of HL-RCOOH.*

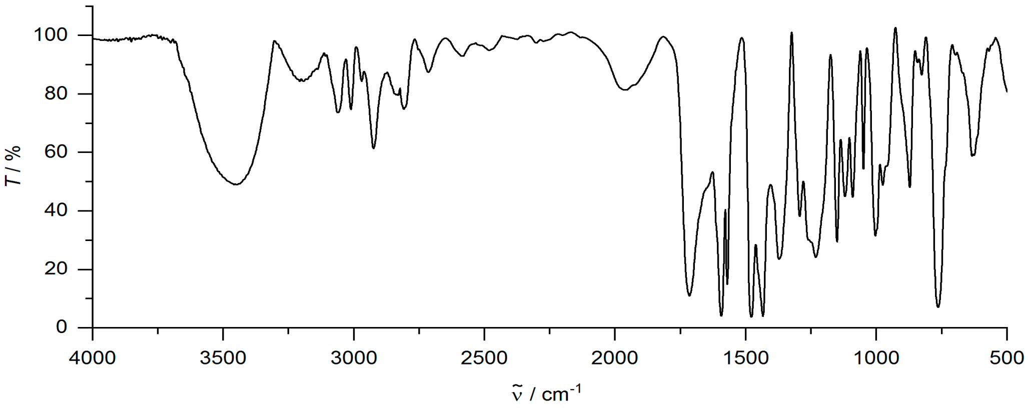

Figure S19: *FT-IR (KBr) spectrum of HL-RCOOH. In addition to the bands described in Fig. S13, the C=O stretching at 1715 cm^{-1} and the C-O stretching at 1232 cm^{-1} of the carboxylic acid moiety can be detected.*

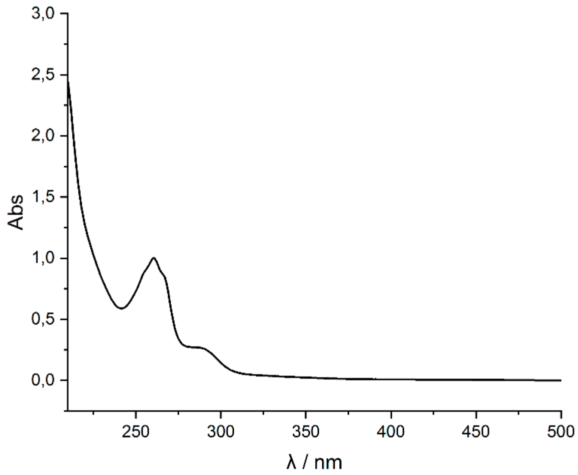

***Figure S20**: UV-vis spectrum of the ligand HL-RCOOH in MeOH (5% v/v)/H_2O, 0.1 mM. A band for the π→π* transition at 262 nm with a shoulder at 430 nm can be observed.*

Synthesis of the complexes

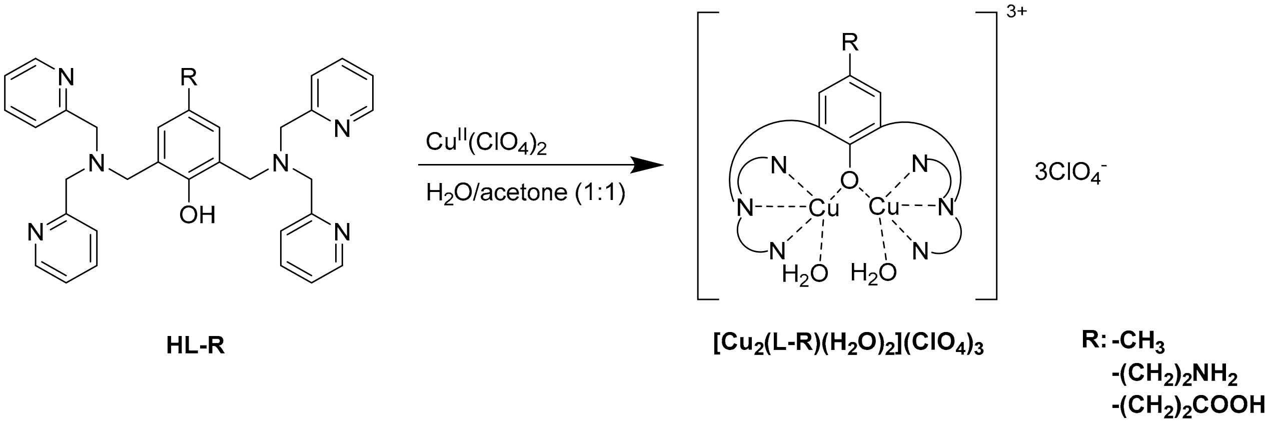

Each complex was synthesized according to *Torelli et al.*[2], although with some modifications in the final step, i.e. the crystallization, which was obtained with the interface crystallization technique: after removing the acetone from the reaction mixture, the solution was left in a vial and closed in a jar containing Et_2O for 12h or more at 4 °C. The crystalline precipitate was then filtered (or centrifuged when more convenient), washed with cold Et_2O and dried in high vacuum overnight, giving the complex in a desirable purity.

Synthesis of $[Cu_2(L\text{-}CH_3)(H_2O)_2](ClO_4)_3$

Ligand HL-CH_3 (58.5 mg, 0.11 mmol) was dissolved in 3 mL of acetone giving a yellow solution. $Cu(ClO_4)_2 \cdot 6H_2O$ (78.5 mg, 0.22 mmol) was dissolved in 3 mL of water and added dropwise to the previous solution under stirring. The mixture turned immediately to dark green. After stirring for 3h

it was isolated with the interface crystallization technique, under Et_2O atmosphere and leaving the solution at 4 °C for 12h. A green powder, after filtration and vacuum drying, was obtained (64.3 mg, 0.06 mmol, yield 55%).

ESI-MS(+) (*m/z*) in CH_3CN: 853.06 $[Cu_2(L\text{-}CH_3)(ClO_4)_2]^+$ (calc. 853.03), 745.08 $[Cu_2(L\text{-}CH_3)(COO)_2]^+$ (calc. 745.13).

FT-IR (KBr) ν (cm^{-1}): 3477 (b), 3083 (w), 2918 (w), 2315 (w), 2287 (w). 2025 (w, b), 1611 (s), 1483 (m), 1445 (m), 1289 (w), 1148 (s), 1120 (s), 1090 (s, b), 859 (w), 772 (m), 623 (s).

Elemental analysis (%): Found: C, 38.49; H, 4.09; N, 7.34. Calc. for $C_{33}H_{41}Cl_3Cu_2N_6O_{17}$ (with two additional water molecules): C, 38.59; H, 4.02; N, 8.18.

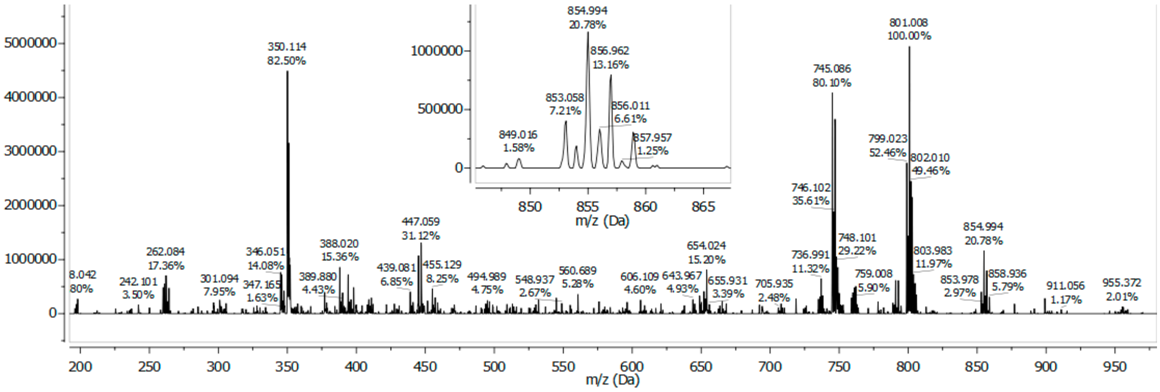

Figure S21: *ESI-MS(+) for* $[Cu_2(L\text{-}CH_3)(H_2O)_2](ClO_4)_3$.

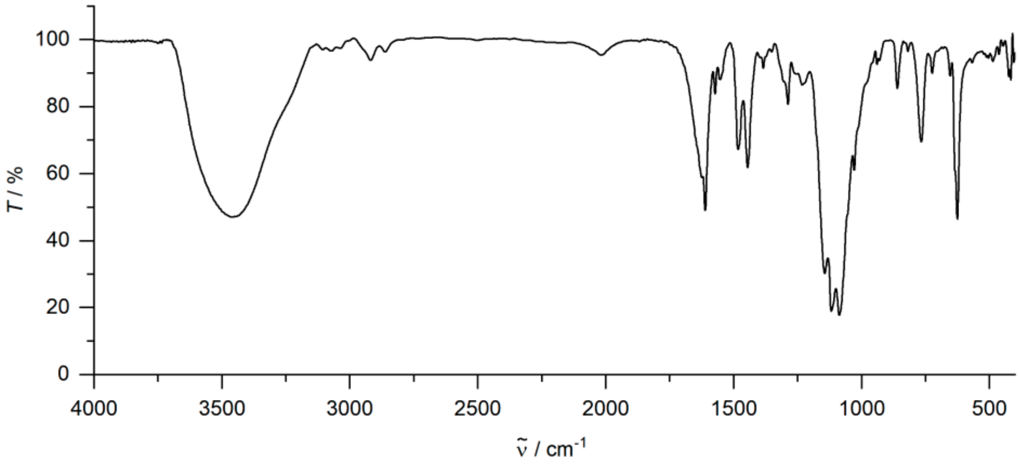

Figure S22: *FT-IR (KBr) spectrum of* $[Cu_2(L\text{-}CH_3)(H_2O)_2](ClO_4)_3$. *The bands at 1118* cm^{-1} *and the sharper band at 625* cm^{-1} *are due to Cu-ligand bonds.*

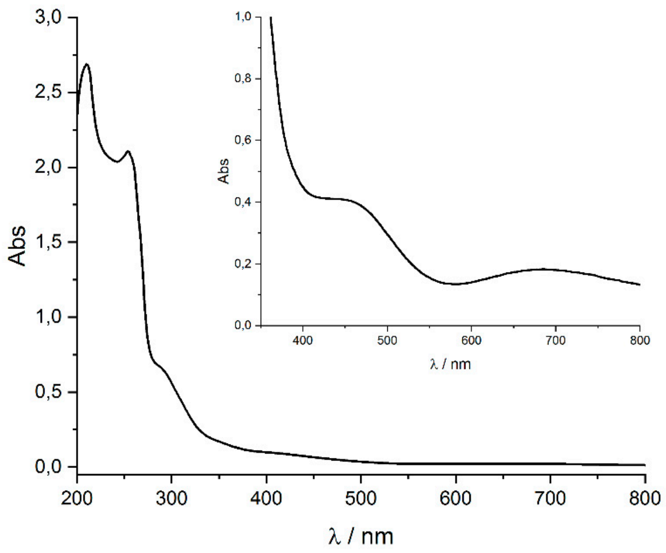

Figure S23: *UV-vis spectrum of the ligand [Cu_2(L-CH_3)(H_2O)$_2$](ClO_4)$_3$ in H_2O, 0.1 mM. Inset: the spectrum collected at 1.5 mM concentration showing the 470 nm (charge transfers from the ligand to the metal) and 700 nm (d → d) bands.*

Synthesis of [Cu_2(L-RNH_2)(H_2O)$_2$](ClO_4)$_3$

HL-RNH_2 (38 mg, 0.07 mmol) was dissolved in 1 mL of acetone. $Cu(ClO_4)_2 \cdot 6H_2O$ (51 mg, 0.14 mmol) dissolved in water (2 mL) was added dropwise under stirring, forming a dark green solution. After 3h of stirring, the complex was obtained with the interface crystallization technique, providing a dark green powder (62 mg, 0.06 mmol, yield 90%).

ESI-MS(+) (*m/z*) in CH_3CN: 774.09 [Cu_2(L-RNH_2)(COO)$_2$]$^+$ (calc. 774.15), 363.06 [Cu_2(L-RNH_2)(COO)]$^{2+}$ (calc. 363.07).

FT-IR (KBr) ν (cm^{-1}): 3449 (b), 2019 (w), 1610 (s), 1572 (w), 1476 (m), 1443 (m), 1384 (w), 1306 (w), 1289 (w), 1263 (w), 1143 (s), 1119 (s), 1087 (s), 1027 (w), 940 (w), 864 (w), 767 (w), 725 (w), 626 (m).

Elemental analysis (%): Found: C, 38.50; H, 4.15; N, 8.79. Calc. for $C_{34}H_{44}Cl_3Cu_2N_7O_{17}$ (with two additional water molecules): C, 38.66; H, 4.20; N, 9.28.

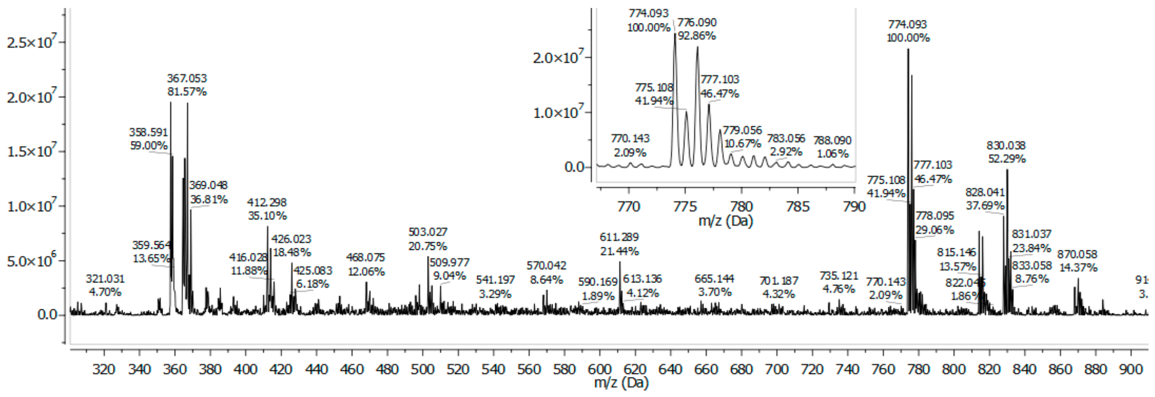

***Figure S24**: ESI-MS(+) for [Cu_2(L-RNH_2)(H_2O)$_2$](ClO_4)$_3$.*

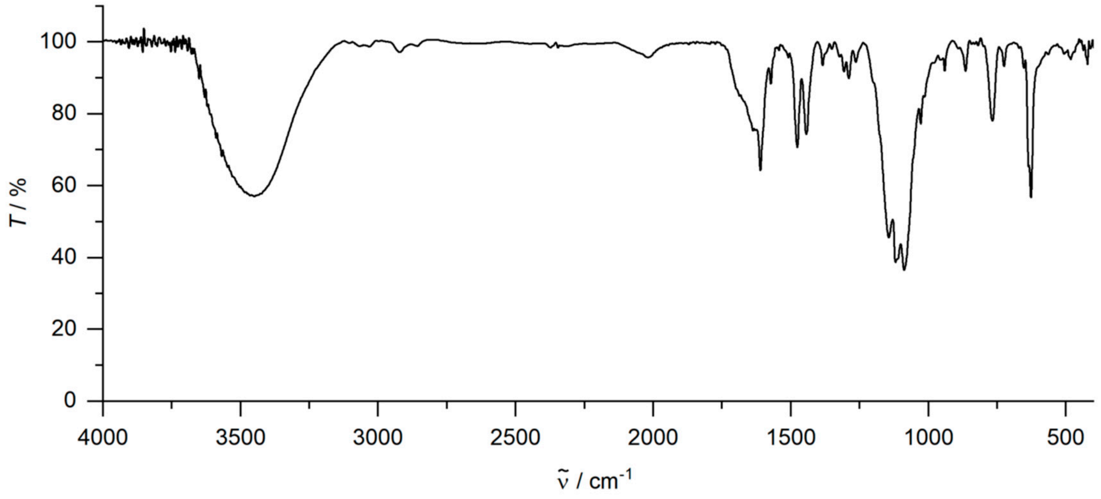

***Figure S25**: FT-IR (KBr) spectrum of [Cu_2(L-RNH_2)(H_2O)$_2$](ClO_4)$_3$. Beside the bands due to the absorption of the ligand, at 1119 cm^{-1} and 626 cm^{-1} the vibrational bands of the bonds including copper can be observed.*

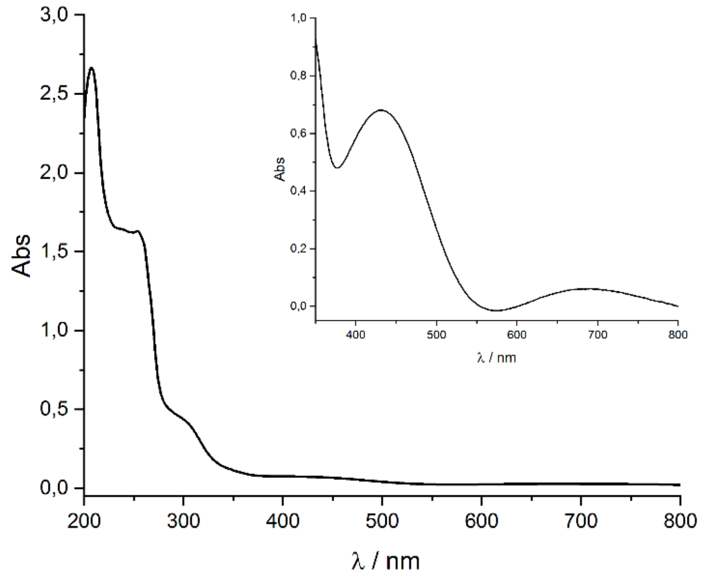

Figure S26: *UV-vis spectrum of the ligand [Cu_2(L-RNH_2)(H_2O)$_2$](ClO_4)$_3$ in H_2O, 0.1 mM. Inset: the spectrum collected at 1.5 mM concentration. The following bands can be observed: π→π* transition of the ligand at 258 nm, charge-transfer-band at 430 nm and a band for d→d transition at 695 nm.*

Synthesis of [Cu_2(L-RCOOH)(H_2O)$_2$(ClO_4)$_3$

HL-RCOOH (101 mg, 0.17 mmol) was solubilized in acetone (1 mL) and $Cu(ClO_4)_2 \cdot 6H_2O$ (125 mg, 0.34 mmol) dissolved in water (3 mL) was added dropwise. After 3h under stirring, the complex was obtained with the interface crystallization technique, leaving the mixture at 4 °C for 12h. A green powder was isolated (45 mg, 0.04 mmol, yield 24%).

ESI-MS(+) (*m/z*) in CH_3CN: 803.09 [Cu_2(L-RCOOH)(COO)$_2$]$^+$ (calc. 803.13), 379.09 [Cu_2(L-RCOOH)(COO)]$^{2+}$ (calc. 379.06).

FT-IR (KBr) ν (cm^{-1}): 3423 (b), 2918 (w), 1719 (w), 1610 (m), 1571 (w), 1560 (w), 1476 (m), 1444 (m), 1384 (w), 1305 (w), 1288 (w), 1145 (s), 1119 (s), 1086 (s), 1028 (w), 940 (w), 864 (w), 767 (w), 724 (w), 635 (m), 626 (m).

Elemental analysis (%): Found: C, 40.86; H, 3.72; N, 7.87. Calc for $C_{35}H_{47}Cl_3Cu_2N_6O_{21}$: C, 40.07; H, 3.75; N, 8.01.

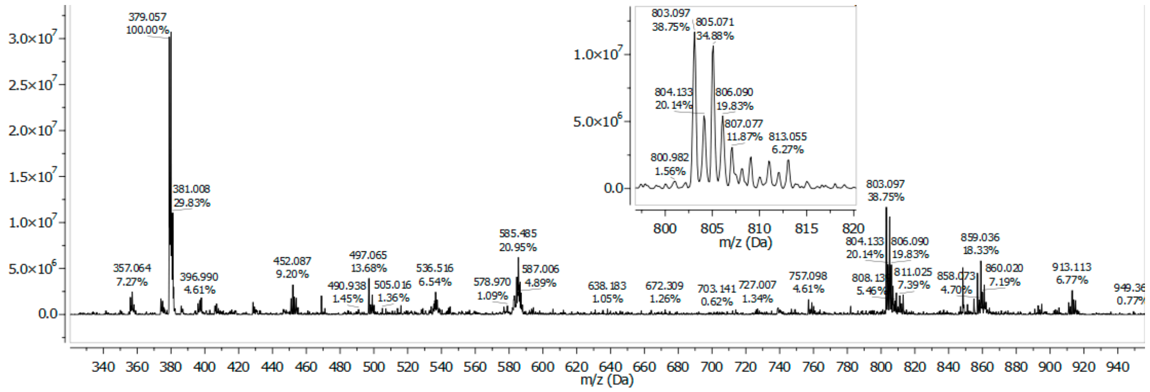

Figure S27: *ESI-MS(+) for [Cu_2(L-RCOOH)(H_2O)$_2$](ClO_4)$_3$.*

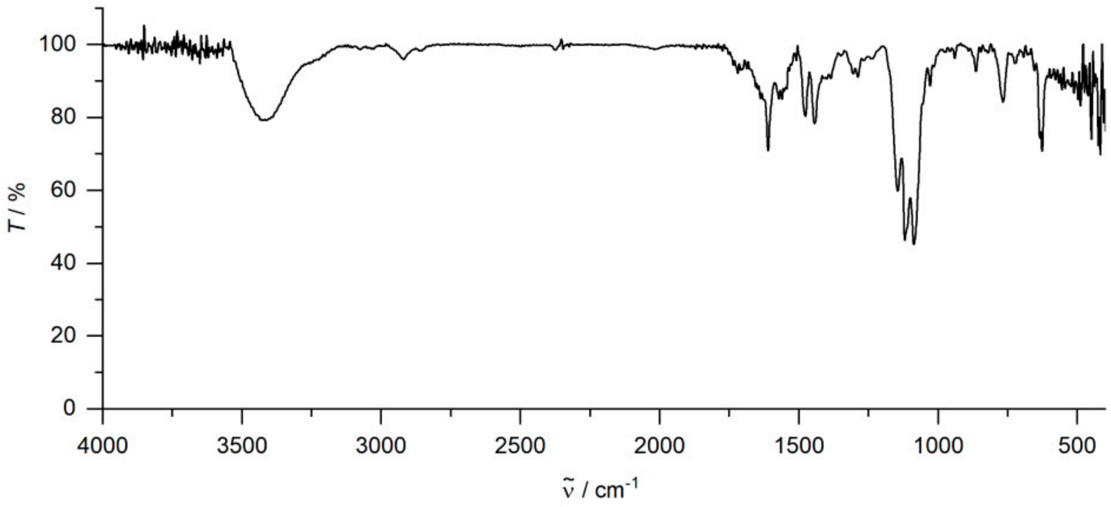

Figure S28: *FT-IR (KBr) spectrum of [Cu_2(L-RCOOH)(H_2O)$_2$](ClO_4)$_3$. The bands at 1143 – 1087 cm^{-1} and the sharp band at 626 cm^{-1} are ascribed to the bonds between Cu and the ligand.*

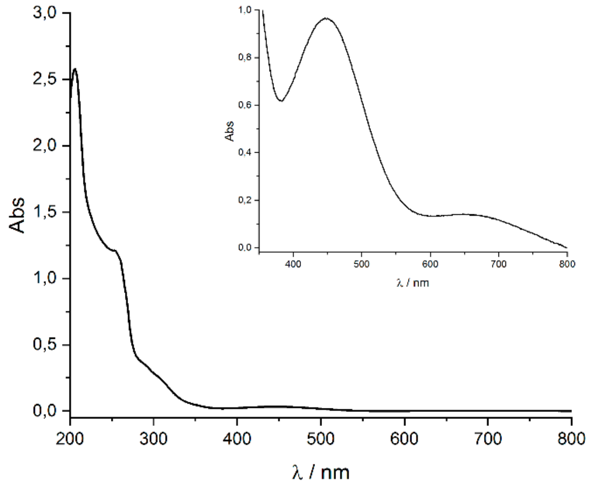

Figure S29: *UV-vis spectrum of the ligand [Cu_2(L-RCOOH)(H_2O)$_2$](ClO_4)$_3$ in H_2O, 0.1 mM.*
Inset: the spectrum collected at 1.5 mM concentration showing the bands at 450 nm and 670 nm (d→d).

Synthesis of HL-RNH-Succ

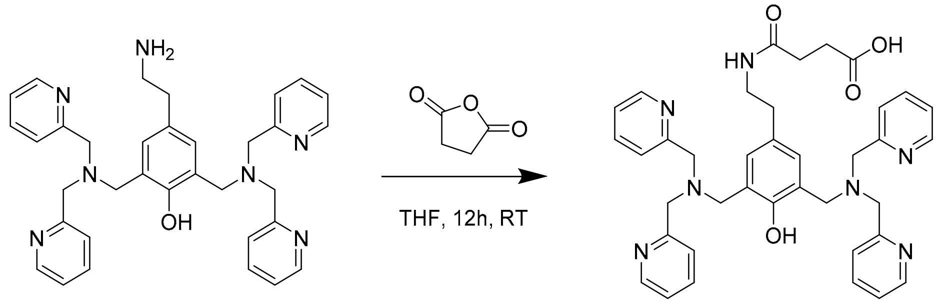

HL-RNH2 (35 mg, 0.062 mmol) was dissolved in 1.5 mL of anhydrous THF. Succinic anhydride (6 mg, 0.062 mmol) was added and the temperature was raised to 60 °C until the precipitate dissolved. The solution was stirred overnight then the solvent was removed, giving a brown oil. The oil was then dissolved in ACN and crystallized at the interface with Et_2O at -20 °C for 36 h, obtaining a light brown sticky solid (13 mg, 0.019 mmol, yield 31%).

^{1}H-NMR (300 MHz, $CDCl_3$) δ (ppm): 8.79 – 8.27 (m, 4H), 7.71 – 7.52 (m, 4H), 7.48 (d, J = 7.7 Hz, 4H), 7.23 – 7.10 (m, 4H), 7.04 (s, 2H), 4.09 (s, 8H), 3.99 (s, 4H), 3.49 (t, J = 6.0 Hz, 2H), 2.71 (dd, J = 7.5, 4.5 Hz, 2H), 2.58 (d, J = 5.0 Hz, 2H), 2.47 – 2.37 (m, 2H).

ESI-MS(+) (*m/z*) in CH_3CN/H^+: 660.25 $[HL\text{-}RNH\text{-}Succ+H]^+$ (calc. 660.33).

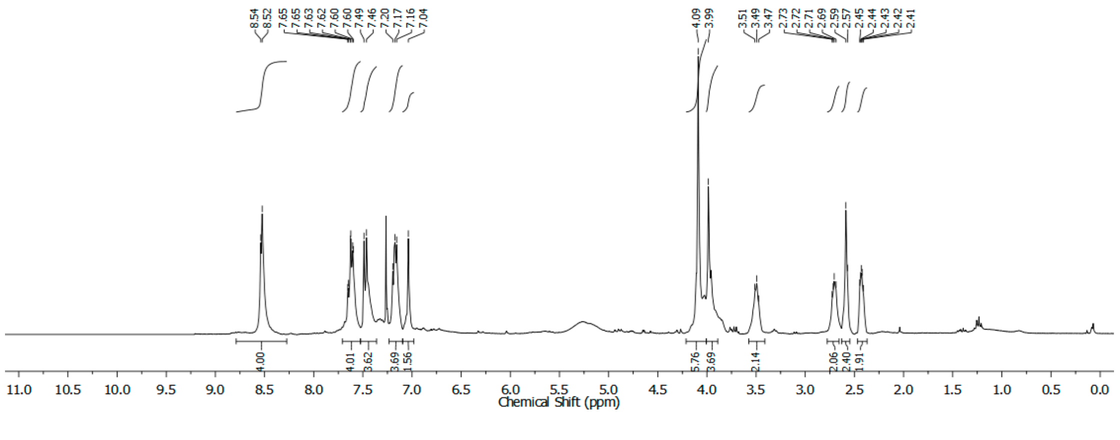

***Figure S30**: ^{1}H-NMR spectrum (300 MHz, $CDCl_3$) of HL-RNH-Succ.*

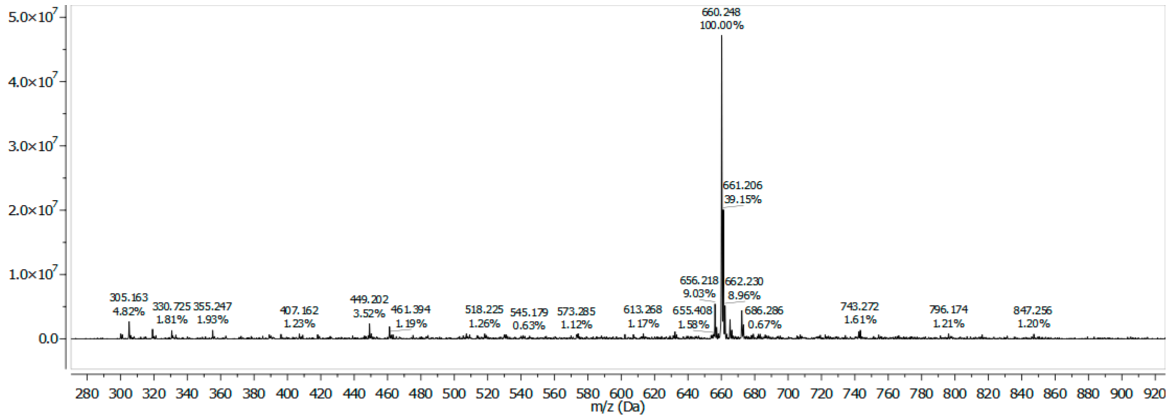

Figure S31: *ESI-MS(+) (m/z) in CH_3CN/H^+ of HL-RNH-Succ.*

Synthesis of KLVFF

The peptide KLVFF was synthesized by manual solid phase using Fmoc/HBTU strategy in 0.1 mmolar scale starting from Wang resin (loading 0.64 mmol/g) to obtain a peptide acid at the C-terminus after cleavage from the resin. A three-fold molar excess of Fmoc-amino acids have been used for each coupling step, in the presence of 6-fold molar excess of DIPEA and using HBTU/HOBt as coupling reagent. Reaction times were 45 min. Fmoc group was then removed using 20% piperidine in DMF. Peptide was simultaneously cleaved from the resin and removed of all the side-chain protecting groups of the amino acid residues to yield the desired peptide by treatment with TFA-triisopropylsilane-H_2O (95:3:2 v/v).

The peptide was purified by preparative RP-HPLC using a Shimadzu LC-8 (Shimazdu, Kyoto, Japan) system with a Juppiter C5, 300Å, 10μ, 250 x 10.00mm column. The column was perfused at a flow rate of 12 mL/min employing a binary gradient system (solvent A: 0.05% TFA in water; solvent B: 0.05% TFA in acetonitrile/water, 9:1 by vol.). The fractions containing the desired peptide were collected and freeze-dried until constant weight.

ESI-MS (+) (MeOH): m/z = 653.30 (calc. for $M+H^+$ $[C_{35}H_{53}N_7O_5]^+$ m/z = 652.8).

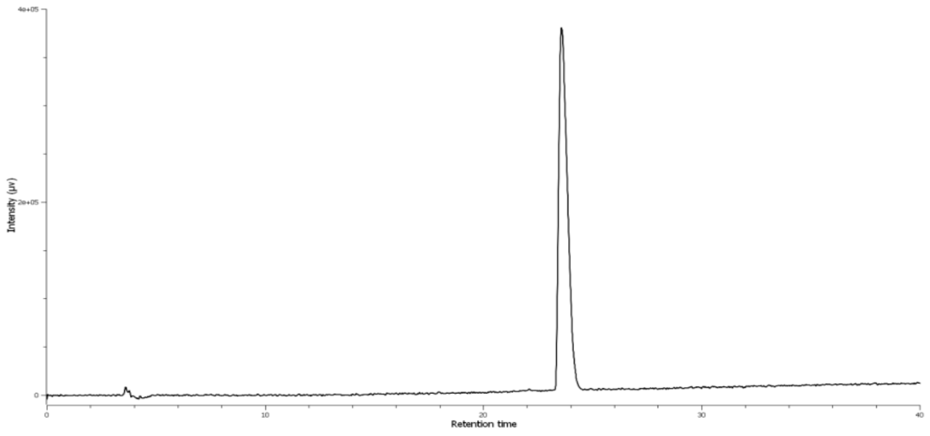

Figure S32: *HPLC of peptide KLVFF. Linear gradient from 5% to 65% of eluent B in 30 min. Eluents: A (0.05% TFA in H2O) , B (0.05% TFA in 9:1 v/v MeCN/H_2O), 1 mL/min flow and UV detector set at 216 nm.*

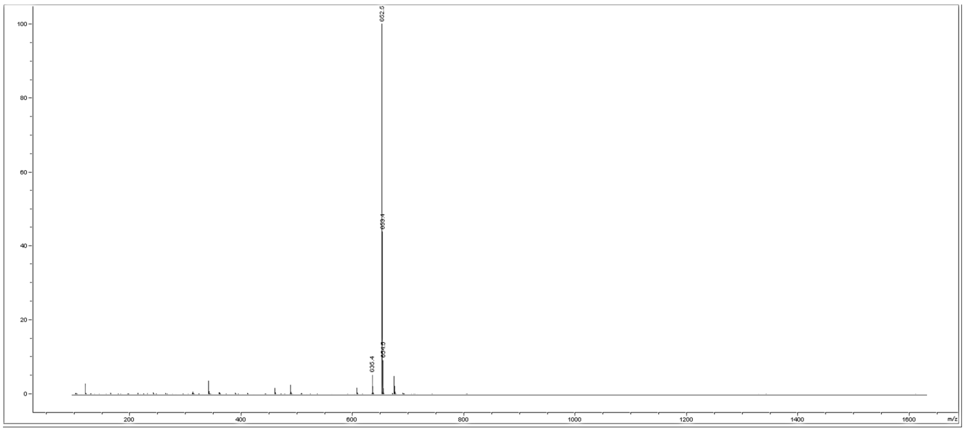

Figure S33: *ESI-MS(+) (m/z) in CH_3OH/H^+ of KLVFF.*

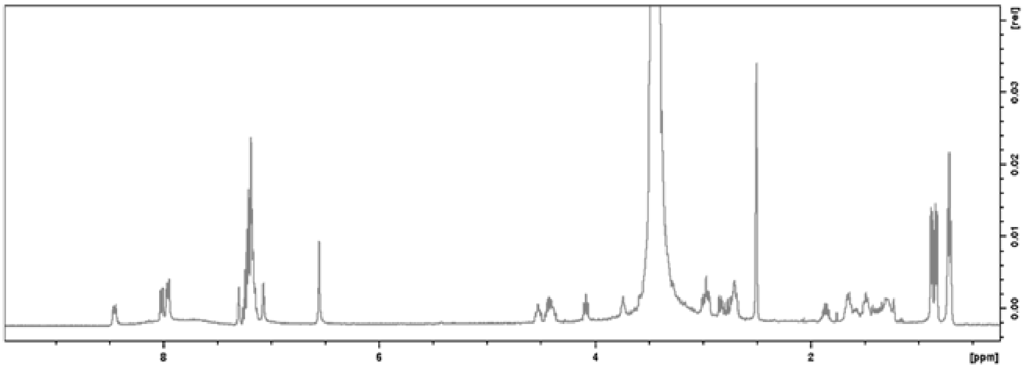

Figure S34: *^{1}H-NMR spectrum (400 MHz, DMSO-$_{d6}$) of KLVFF.*

Synthesis of HL-RNH-Succ-KLVFF

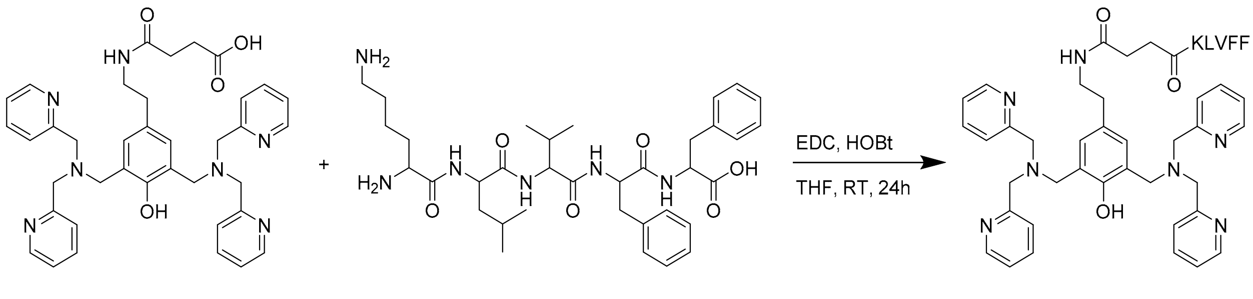

KLVFF

HL-RNH-Succ

HL-RNH-Succ-KLVFF

Under anhydrous conditions, HL-RNH-Succ (10 mg, 0.015 mmol) was dissolved in 2 mL of anhydrous DCM. HOBt (7 mg, 0.051 mmol) was then added. EDC/HCl (6 mg, 0.031 mmol) was dissolved in 2 mL of anhydrous ACN and neutralized with 15 µL of NEt_3. This second solution was added dropwise to the previous one, at 0 °C and left stirring for 45 minutes. KLVFF (10 mg, 0.015 mmol) was then dissolved in 1.5 mL of anhydrous DMF and added to the previous solution. After 24h under stirring, the solvent was removed, obtaining a brown oil.

ESI-MS(+) (*m/z*) in MeOH/H^+: 1293.69 $[HL\text{-}RNH\text{-}Succ\text{-}KLVFF+H]^+$ (calc. 1293.72).

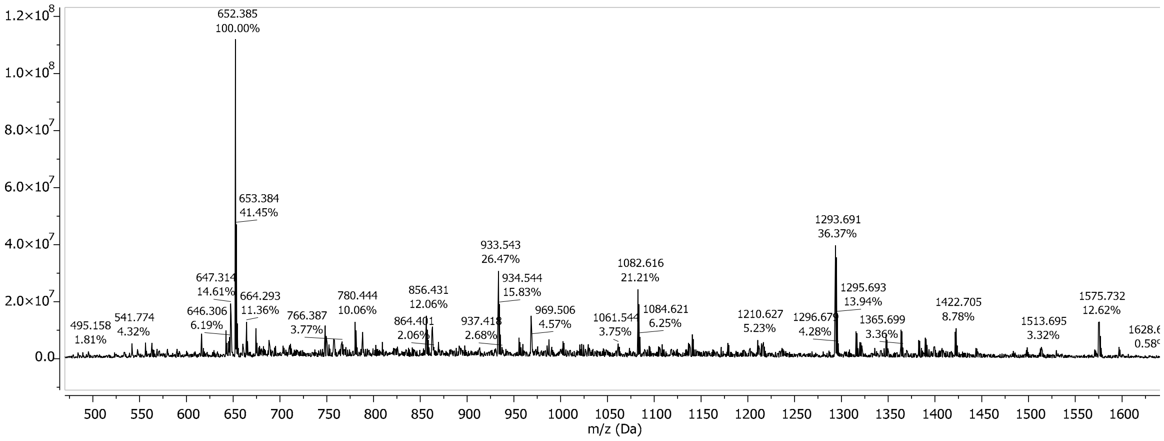

Figure S35*: ESI-MS(+) spectrum of HL-RNH-Succ-KLVFF. The peaks are at 1293.69* m/z *(calc. 1293.72* m/z*) for the product, at 652.39* m/z *for the KLVFF (calc. 652.85* m/z *).*

Spectroscopic measurements

UV-vis spectra of ligands and complexes were recorded in a quartz cuvette, with 1 cm of optical path. For the ligands, a 0.1 mM solution in MeOH/H_2O (5% v/v) was used. For the complexes, 0.1 mM to 1.5 mM solutions (to highlight d-d bands) were prepared in deionized water.

3. Study of the complexation through UV-vis spectroscopy

For the determination of the binding constant between metals and ligands, a solution of the latter was titrated with 0.2 eq of metal for each addition.

The titrating solutions were 10 mM and 100 µM for $Cu(ClO_4)_2$ versus 0.1 mM and 1 µM for the ligands in 5% v/v MeOH/BBS (50 mM, pH 7.4), respectively. For the other metals (Mn^{2+}, Fe^{2+} and Zn^{2+}) the solutions were 10 mM for the relative perchlorate salt and 0.1 mM for the ligand (HL-CH_3), always in 5% v/v MeOH/BBS (50 mM, pH 7.4). The measurements were collected in a quartz cuvette, with 1 cm of optical path, filled with 2.5 mL of the ligand solution and aliquots of 5 µL of the metal solution were added (0.2 eq of M^{2+} for each). The absorbance variation was monitored from 200 nm to 800 nm with a Varian Cary 50 instrument, thermostated at 25 °C.

The binding constants were then determined by nonlinear regression analysis of the plot "*ΔAbs/b vs Cu(II) concentration" (Equation* 7), according to K. Connors, *The Measurement of Molecular Complex Stability*, Madison, Wisconsin, Wiley, 1987[5].

$$\frac{\Delta A}{b} = \frac{(K_1 \Delta A_1[Cu] + \beta_{1,2}\Delta A_2[Cu]^2)}{1+K_1[Cu] + \beta_{1,2}[Cu]^2} \qquad (7)$$

ΔA: absorbance at a certain wavelength.

B: optical path (1 cm).

K_1: constant for the formation of the complex 1:1.

$\beta_{1,2}$: global formation constant (K_{bind}) for the complex 1:2.

ΔA_1 and ΔA_2 differences in absorbance due to the 1:1 and 1:2 complex, estimated graphically at 1 and 2 eq. of copper, respectively.

[5] Connors, K. A.; *The Measurement of Molecular Complex Stability*. Wiley-interscience publication, **1987**, Madison, Wisconsin (U.S.A).

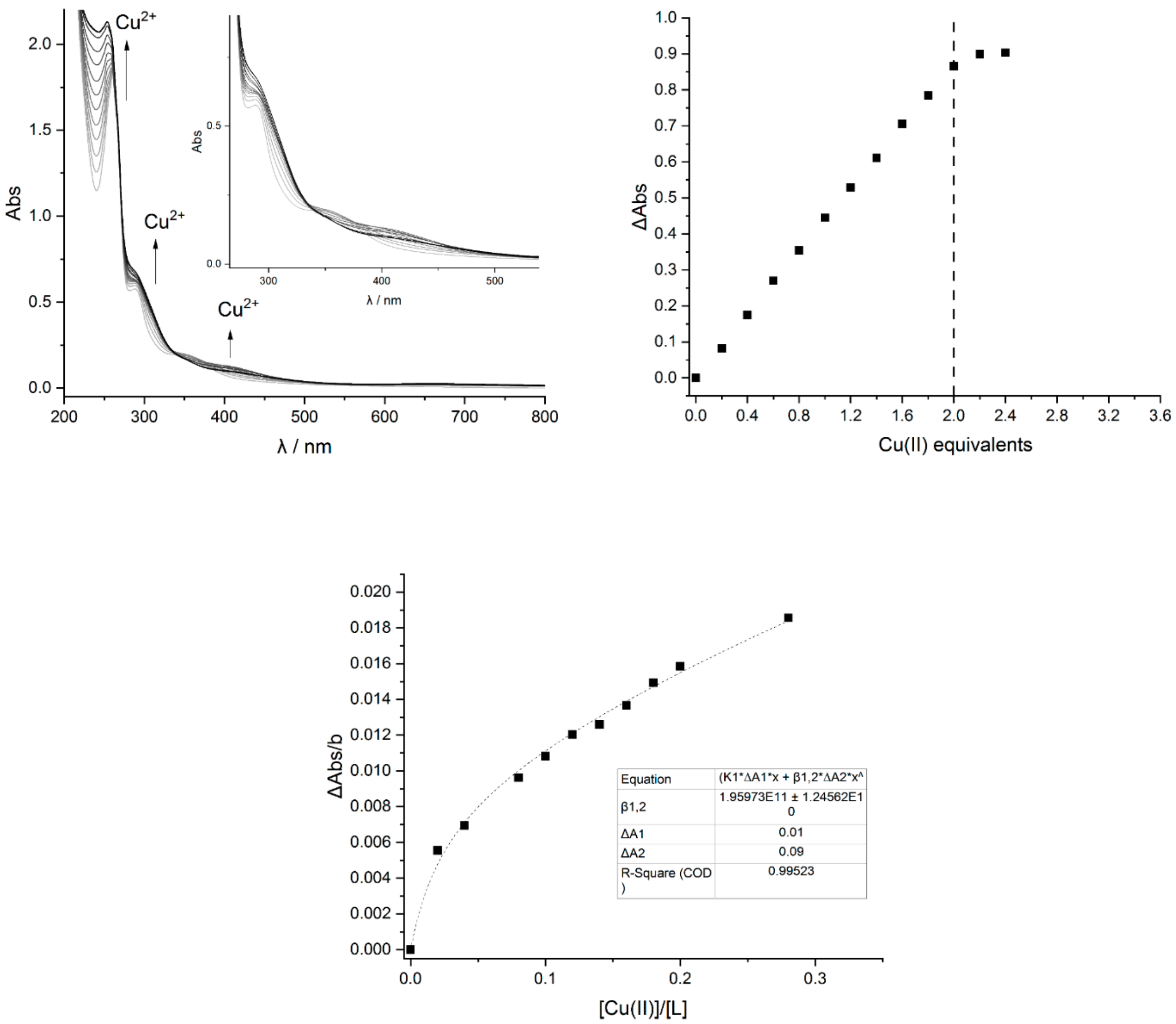

Figure S36: *Top left: UV-vis spectra of HL-CH$_3$ titration with Cu(ClO$_4$)$_2$. Ligand concentration 0.1 mM, each addition corresponds to 0.2 eq of Cu^{2+}. In this case an isosbestic point at 340 is observable. Top right: plot ΔAbs at 240 nm vs Cu(II) equivalents for the titration of HL-CH$_3$. Bottom: Nonlinear regression analysis for the plot ΔAbs/b at 240 nm vs Cu(II) concentration for the titration of HL-CH$_3$ (1 μM).*

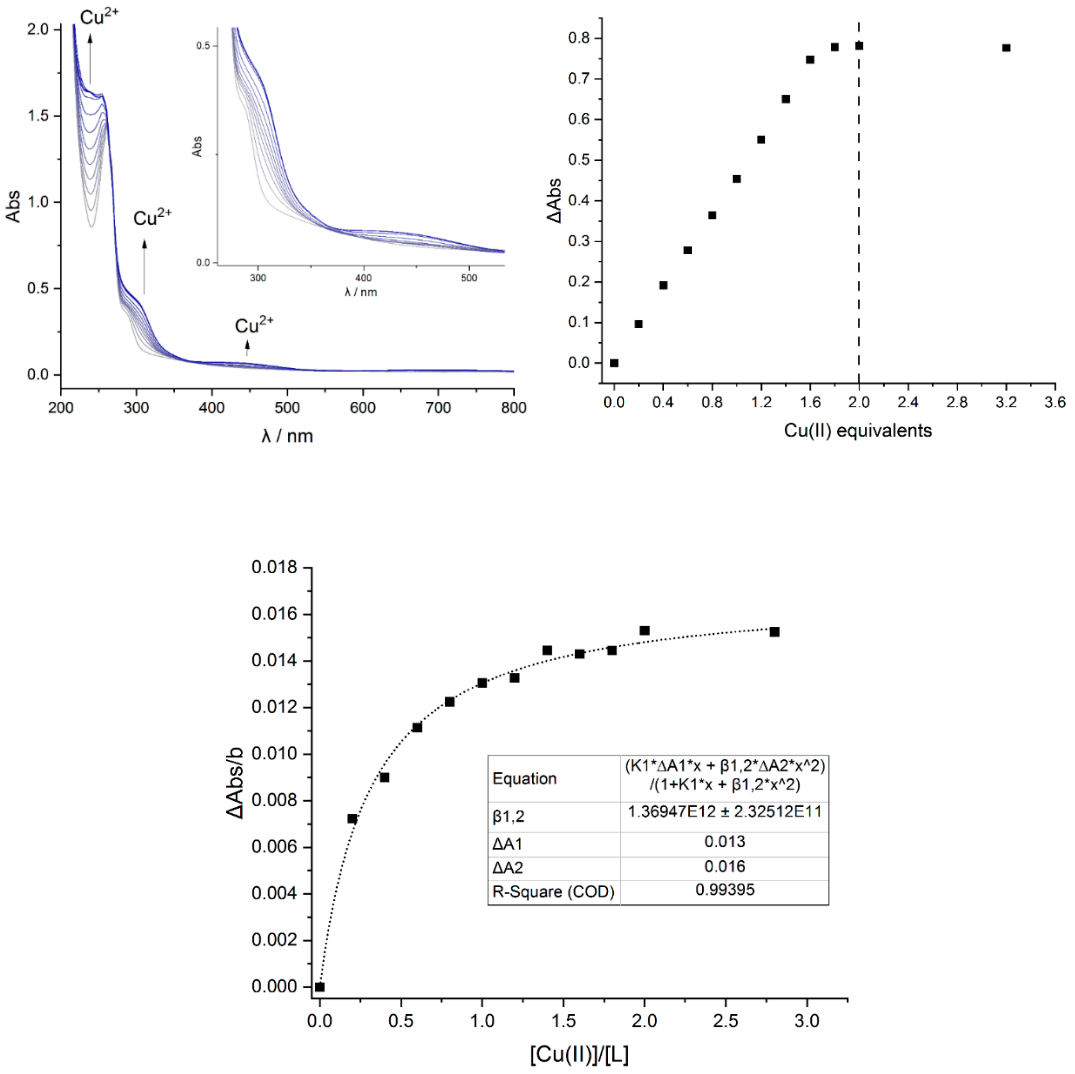

Figure S37: *Top left: UV-vis spectra of HL-RNH$_2$ titration with Cu(ClO$_4$)$_2$. Ligand concentration 0.1 mM, each addition corresponds to 0.2 eq of Cu^{2+}. Top right: Plot ΔAbs at 240 nm vs Cu(II) equivalents for the titration of HL-RNH$_2$; Bottom: Nonlinear regression analysis for the plot ΔAbs/b at 240 nm vs Cu(II) concentration for the titration of HL-RNH$_2$ (1 μM).*

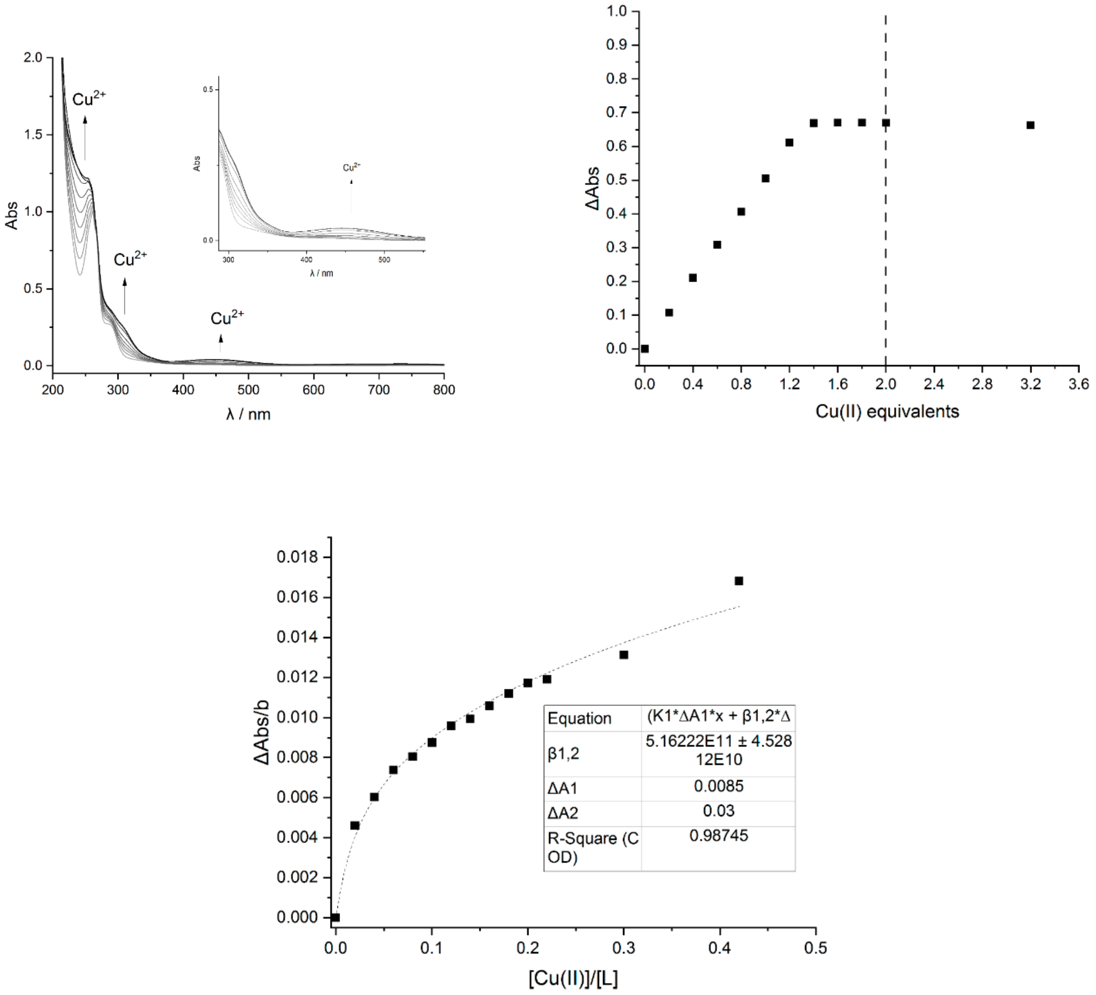

Figure S38*: Top left: UV-vis spectra of HL-RCOOH titration with $Cu(ClO_4)_2$. Ligand concentration 0.1 mM, each addition corresponds to 0.2 eq of Cu^{2+}. Top right: Plot ΔAbs at 260 nm vs Cu(II) equivalents for the titration of HL-RCOOH. Bottom: Nonlinear regression analysis for the plot ΔAbs/b at 260 nm vs Cu(II) concentration for the titration of HL-RCOOH (1 µM).*

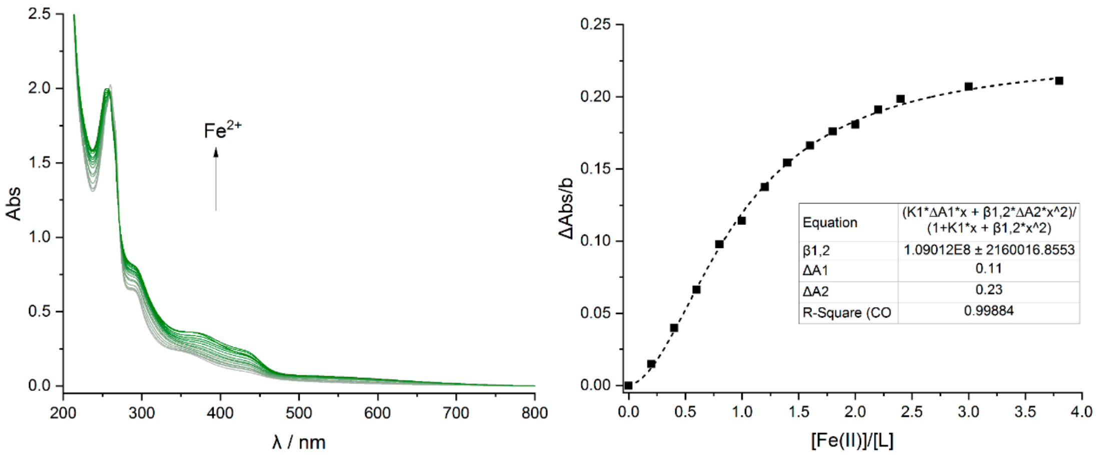

Figure S39: *UV-vis spectra of HL-CH_3 titration with $Fe(ClO_4)_2$. Ligand concentration 0.1 mM, each addition corresponds to 0.2 eq of Fe^{2+}; Nonlinear regression analysis for the plot ΔAbs/b at 310 nm vs Fe(II) concentration for the titration of HL-CH_3.*

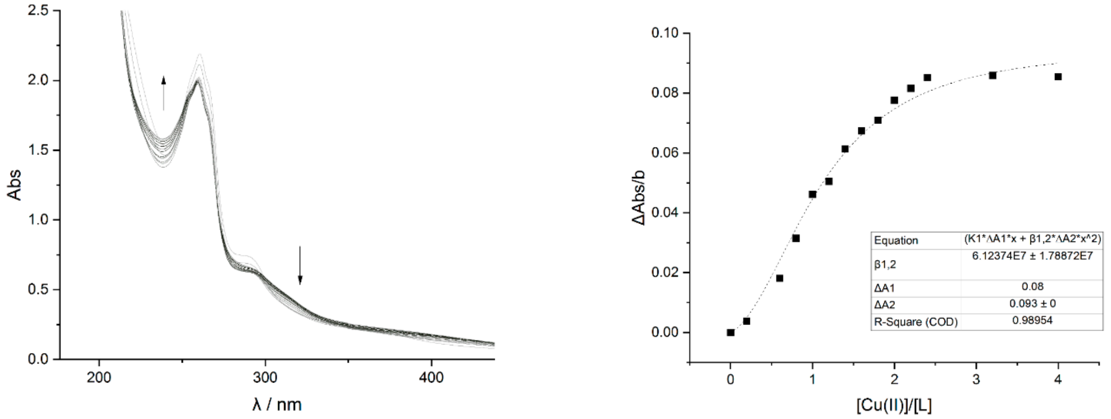

Figure S40*: UV-vis spectra of HL-CH_3 titration with $Mn(ClO_4)_2$. Ligand concentration 0.1 mM, each addition corresponds to 0.2 eq of Mn^{2+}. Nonlinear regression analysis for the plot ΔAbs/b at 310 nm vs Mn(II) concentration for the titration of HL-CH_3.*

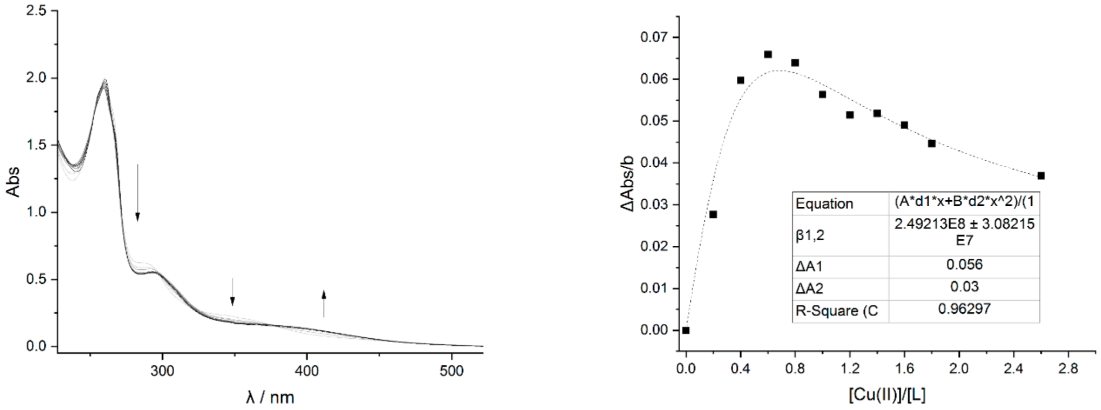

Figure S41: *UV-vis spectra of HL-CH$_3$ titration with Zn(ClO$_4$)$_2$. Ligand concentration 0.1 mM, each addition corresponds to 0.2 eq of Zn^{2+}. Nonlinear regression analysis for the plot ΔAbs/b at 310 nm vs Zn(II) concentration for the titration of HL-CH$_3$.*

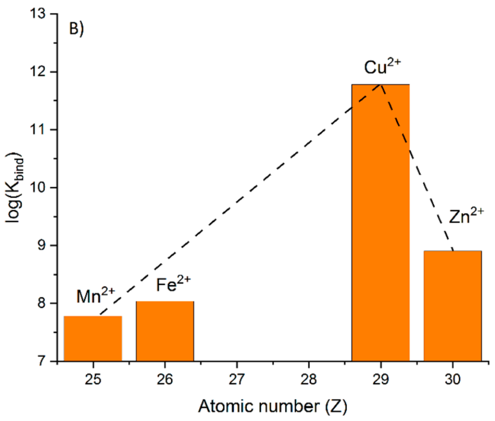

Figure S42: *The Irving-Williams Series for the K_{bind} of HL-CH$_3$ with different metals.*

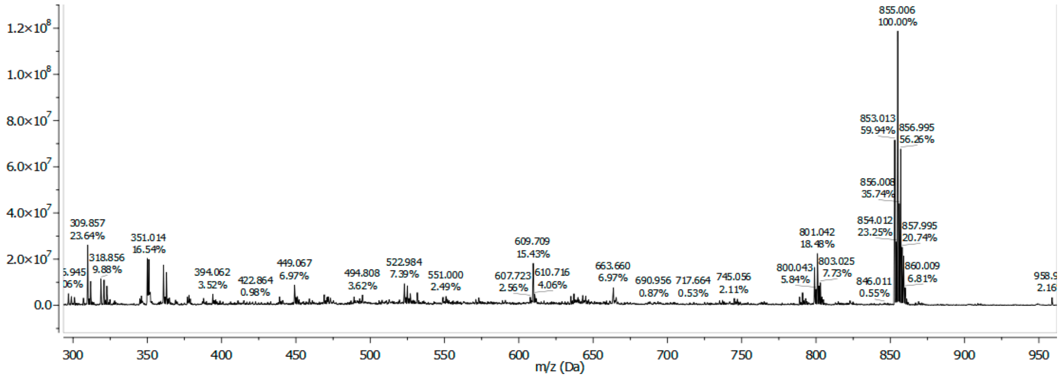

Figure S43: *ESI-MS(+) spectrum in MeOH (+1% HCOOH) of an equimolar solution of Cu(ClO$_4$)$_2$, Fe(ClO$_4$)$_2$, Mn(ClO$_4$)$_2$ and Zn(ClO$_4$)$_2$ and half molar for HL-CH$_3$.*

4. Cyclic voltammetry

Cyclic voltammetry was carried out using a classical three-electrodes system: a glassy carbon electrode as working electrode (3 mm diameter, geometric surface area 7 mm^2), a platinum wire as counter electrode and a Ag/AgCl reference electrode (3 M KCl). The voltammograms were collected in a phosphate buffer (PBS) 10 mM with NaCl 150 mM in deionized water, as supporting electrolyte. For each complex, a solution 0.25 mM in PBS was prepared (10 mL), with the help of sonication (15 min approx.). Before and after each acquisition the working electrode was cleaned with alumina paste via mechanical polishing, then sonicated. Nitrogen was used as purging gas to remove oxygen inside the cell. It bubbled for at least 10 minutes before the first measurement and then left above the solution. Also, a measurement of solution's behavior was taken before the addition of the analyte. The voltammograms were recorded at different scan rates: 50, 100, 200, 400 and 800 mV/s. For $K_3Fe(CN)_6$ the voltammogram was recorded at concentration 10.2 mM in PBS and scan rate 200 mV/s.

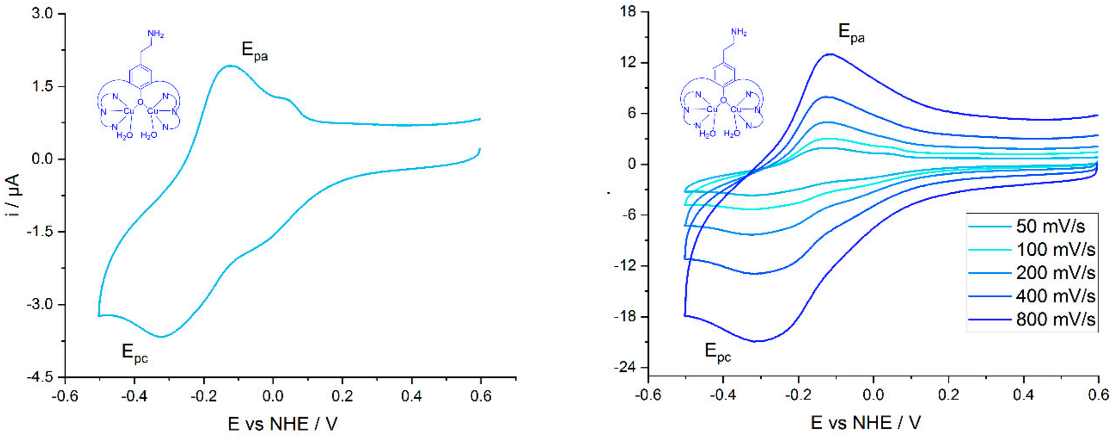

(a)

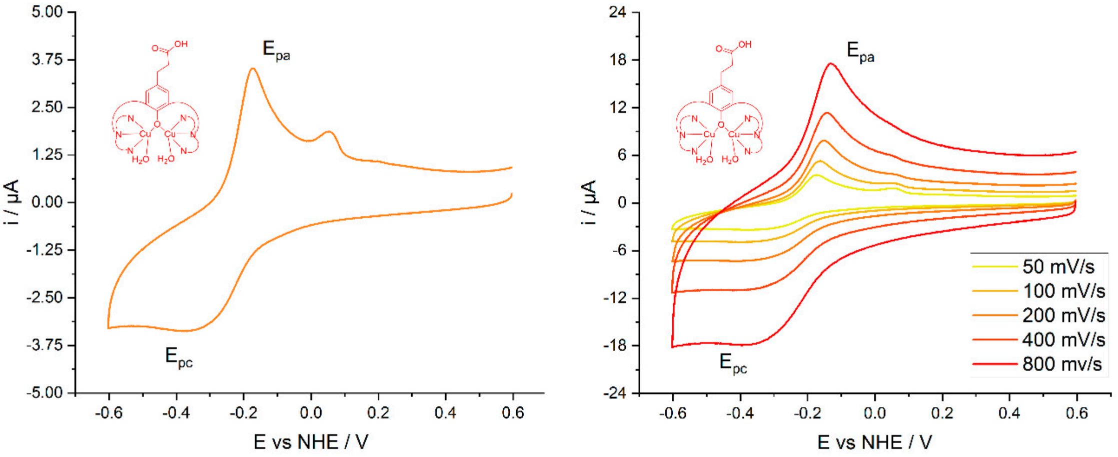

(b)

Figure S44: *Cyclic voltammograms of the complexes 0.25 mM in PBS (10 mM, 150 mM NaCl, pH 7.8). (a)* $[Cu_2(L\text{-}RNH_2)(H_2O)_2](ClO_4)_3$*, (b)* $[Cu_2(L\text{-}RCOOH)(H_2O)_2](ClO_4)_3$*. Scan rate 50 mV/s (left) and different scan rates (right). For the substituted complex, a shoulder is present around 0V and is tentatively attributed to the reduction of* $Cu_2^{II,II/II,I}$.

5. Measurement of the catalytic activity

Superoxide radical anion dismutation (SOD)

For the determination of the SOD-like activity the procedure described by *McCord & Fridovich*[6] was followed, with some minor modifications. It consists in an indirect competitive method using cytochrome c. This enzyme oxidizes the superoxide radical anion, produced in situ by xanthine/Xanthine oxidase system, to oxygen gas reducing itself to ferricytochrome c which absorbs at 550 nm. This wavelength was monitored via UV-vis spectroscopy. When the complex was added, it competed with cytochrome c to oxidize $O_2^{\bullet-}$, leading to a lower absorbance. Catalase is also added to remove the hydrogen peroxide produced by the complex in the reaction of dismutation.

The assay was performed directly in a cuvette (3 mL) with 1 cm of optical path containing:

- Cytochrome c (10 µM) solution in PBS (50 mM with 150 mM NaCl, pH 7.8), containing 40 µM of Xanthine;
- Catalase solution (15 mg/L) in deionized water;
- Copper complex solution (100 µM) in deionized water;
- PBS (50 mM with 150 mM NaCl, pH 7.8), to maintain constant the total volume;
- Xanthine oxidase solution (0.795 U/mL).

Table S1: Summary of the four solutions prepared in the SOD assay.

Reagent addition order	1st measure	2nd measure	3rd measure	4th measure
Cytochrome c solution	3 mL	3 mL	3 mL	3 mL
Catalase solution	10 µL	10 µL	10 µL	10 µL
PBS	30 µL	25 µL	20 µL	10 µL
Copper complex solution	0 µL	5 µL	10 µL	20 µL
Xanthine oxidase	20 µL	20 µL	20 µL	20 µL

6 McCord J; Fridovich I.; McCord, J. M.; Fridovich, I.; *J. Biol. Chem.*, **1969**, 244, 6049-6055.

The experiments were conducted at 25 °C, with xanthine oxidase added at the final stage, as the production of the superoxide radical anion is instantaneous. Since the cuvettes lacked a mixing system, the reactants were mixed manually by shaking each cuvette overhead three times before taking measurements As the kinetics are very fast, only the first linear part ($t < 9$ s) of the graph "*Abs at 550 nm vs time*" was considered for the calculation of cytochrome c inhibition. Here, *m* is the slope these lines. The percentage of inhibition for different SOD mimic concentrations is calculated in *Equation 1*.

$$\%\text{inhibition} = \left(1 - \frac{m_{\text{inhibited}}}{m_{\text{uninhibited}}}\right) \cdot 100 \qquad (1)$$

IC_{50}, the concentration of complex needed to inhibit 50% of cytochrome c, can be calculated with *Equation 2*, where *a* and *b* are the intercept and the slope of the graph "*%inhibition vs [Cu] concentration*".

$$IC_{50} = \frac{(50-a)}{b} \qquad (2)$$

Finally, the kinetic rate constant for the dismutation of $O_2^{\bullet-}$ by the complex can be calculated with *Equation 3*. The value of the kinetic constant for $O_2^{\bullet-}$ scavenge by cytochrome c is known from literature and is equal to $2.6 \cdot 10^6$ M^{-1} s^{-1} [6].

$$k_{\text{SOD complex}}(O_2^{\bullet-}) = \frac{k_{\text{CytC}}\,(O_2^{\bullet-})}{IC_{50}} \qquad (3)$$

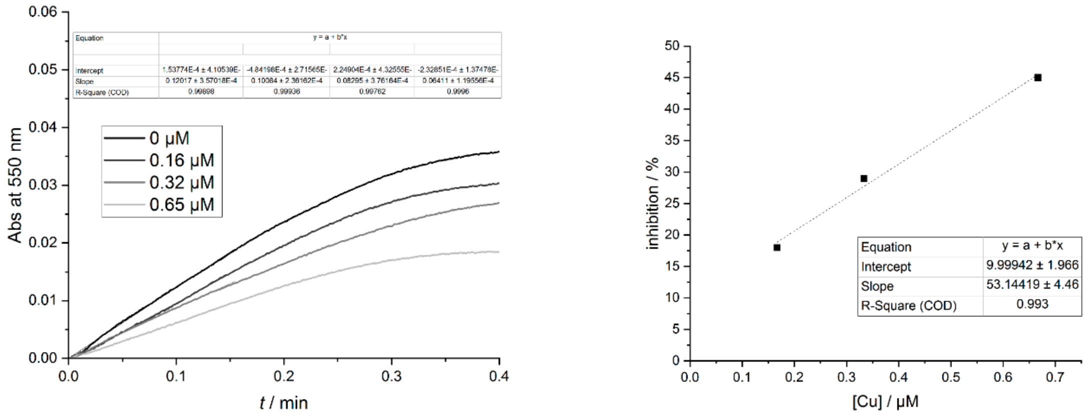

***Figure S45**: SOD kinetics of $[Cu_2(L\text{-}CH_3)(H_2O)_2](ClO_4)_3$ using McCord & Fridovich enzymatic assay. UV-vis absorbance at 550 nm at different concentration of complex (0, 0.16, 0.32, 0.65 µM) monitored in function of time. Graph and linear regression of the values of %inhibition vs copper complex concentration for $[Cu_2(L\text{-}CH_3)(H_2O)_2](ClO_4)_3$.*

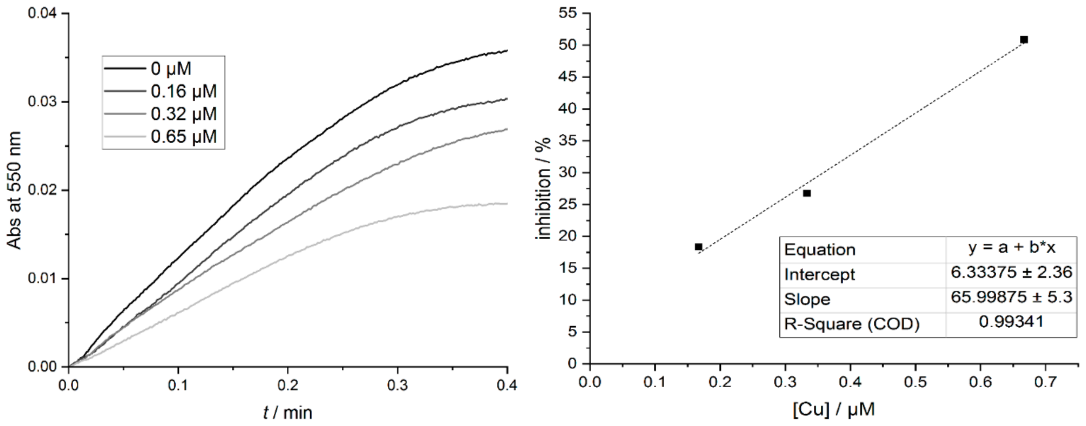

Figure S46*: SOD kinetics of [Cu_2(L-RNH_2)(H_2O)$_2$](ClO_4)$_3$ using McCord & Fridovich enzymatic assay. UV-vis absorbance at 550 nm at different concentration of complex (0, 0.16, 0.32, 0.65 µM) monitored in function of time. Graph and linear regression of the values of %inhibition vs copper complex concentration for [Cu_2(L-RNH_2)(H_2O)$_2$](ClO_4)$_3$.*

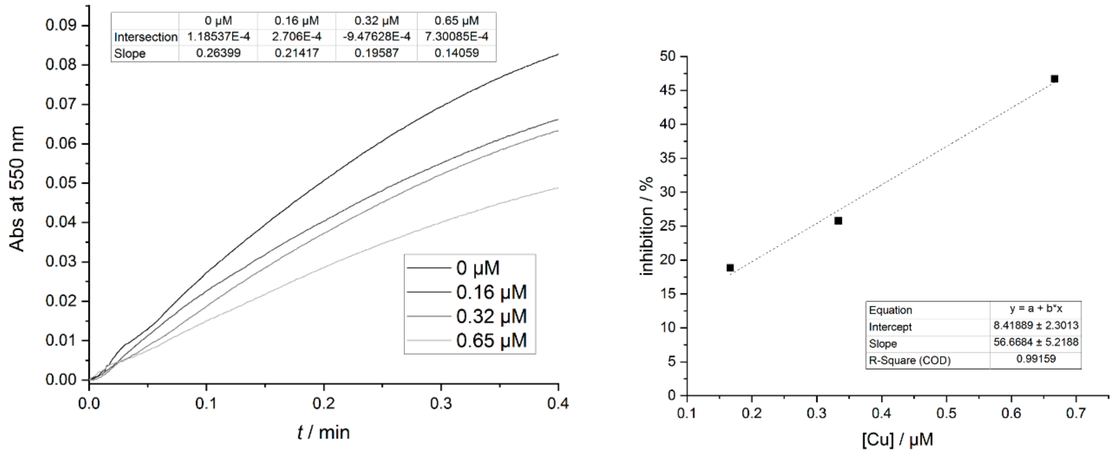

Figure S47*: SOD kinetics of [Cu_2(L-RCOOH)(H_2O)$_2$](ClO_4)$_3$ using McCord & Fridovich enzymatic assay. UV-vis absorbance at 550 nm at different concentration of complex (0, 0.16, 0.32, 0.65 µM) monitored in function of time. Graph and linear regression of the values of %inhibition vs copper complex concentration for [Cu_2(L-RCOOH)(H_2O)$_2$](ClO_4)$_3$.*

Table S2. Summary of SOD activity for native Cu/Zn-SOD and different metal complexes.

Complexes	IC_{50} (µM)	log(k_{SOD})	Ref.
**Cu/Zn-SOD$_{(human)}$**	0.0013^b	9.30	[7]
**$CuSO_4$**	72.2^a	-	[8]
**M40403**	-	7.21^c	[9]
**[Mn(3)(OTf)$_2$]**	0.75^b	6.90	[10]
**$Mn_2L'_2P_2^{6+}$**	0.22^a	7.05	[11]
**[Cu_2(L-CH_3)(H_2O)$_2$](ClO_4)$_3$**	0.75^a	6.54	this work^d
**[Cu_2(L-RNH_2)(H_2O)$_2$](ClO_4)$_3$**	0.66^a	6.59	this work^d
**[Cu_2(L-RCOOH)(H_2O)$_2$](ClO_4)$_3$**	0.72^a	6.56	this work^d
**[Cu(PBMPA)]$^+$**	1.04^a	7.09	[12]
**$Cu_2L^3_2$**	0.072^a	7.55	[13]
**[CuPy^{iPr}N$_2$^{Me}N]$^{2+}$**	0.26	7.13	[14]

IC_{50} and log(k_{SOD}) were determined with different methods: a) cyt c assay, b) NBT assay, c) stopped-flow kinetic assay. L^3: 2-({[di(2-pyridyl)-methyl]amino}methyl)-4-nitrophenol. L′: 2-{[[di(2pyridyl)methyl](methyl)amino]-methyl}phenol). 3: 2,6-bis([(2-pyridylmethyl)thio]methyl)pyridine. ^{d}IC_{50} and log(k_{SOD}) values for the three synthesized complexes were measured in PBS (50 mM, 150 mM NaCl) at pH 7.8 and 25 °C.

[7] Ramadan, A. E.-M. M. M.; *J. Coord. Chem.*, **2012**, 65, 1417–1433.
[8] Iranzo, O.; *Bioorg. Chem.*, **2011**, 39, 73–87.
[9] Shank, M.; Barynin, V.; Dismukes, G. C.; *Biochemistry*, **1994**, 33, 15433–15436.
[10] Grau, M.; Rigodanza, F.; White, A. J. P. P.; Sorarù, A.; Carraro, M.; Bonchio, M.; Britovsek, G. J. P. P.; *Chem. Commun.*, **2014**, 50, 4607–4609.
[11] Squarcina, A.; Sorarù, A.; Rigodanza, F.; Carraro, M.; Brancatelli, G.; Carofiglio, T.; Geremia, S.; Larosa, V.; Morosinotto, T.; Bonchio, M.; *ACS Catal.*, **2017**, 7, 1971–1976.
[12] Pap, J. S.; Kripli, B.; Bors, I.; Bogáth, D.; Giorgi, M.; Kaizer, J.; Speier, G.; *J. Inorg. Biochem.*, **2012**, 117, 60–70.
[13] Squarcina, A.; Santoro, A.; Hickey, N.; De Zorzi, R.; Carraro, M.; Geremia, S.; Bortolus, M.; Di Valentin, M.; Bonchio, M.; *ACS Catal.*, **2020**, 10, 7295–7306.
[14] Mekhail, M.A.; Smith, K.J.; Freire, D.M.; Pota, K.; Nguyen, N.; Burnett, M.E.; Green, K.N. *Inorg. Chem.* 2023, 62, 14, 5415–5425

Catalase-like activity (CAT)

For the determination of the CAT-like activity of the complex, the increase of pressure due to oxygen production from the hydrogen peroxide dismutation was monitored over the course of 22h. A large neck vial (25 mL) was filled with 10.4 mL of borate buffer (BBS 50 mM with 150 mM of NaCl, pH 7.81), 15 or 20.5 μL of H_2O_2 (50%, 17.5 M) and connected to a pressure transducer. When the signal was stable enough, 1.6 mL of a complex solution (1.5 mM in deionized water) was added through a septum under mixing and the measurement started ([H_2O_2] = 22.5 or 30 mM respectively). The temperature was kept constant at 25 °C with a thermostat for the whole time of the experiment. The head space volume was 12.98 mL approximately and "*Ideal gas law*" was used to obtain the number of moles of oxygen produced in the reaction, according to *Equation 4*.

$$n_{O_2} = \frac{\Delta P \cdot V}{R \cdot T} \qquad (4)$$

The initial rates R_0 of oxygen evolution were calculated by linear regression at maximum slope of the curve "*μmol O_2 vs time*", at conversion of H_2O_2 < 10%.

The rate constants k_{CAT} were calculated according to [15], with linear regression of pseudo-first-order rate constants (*Equation 5*).

$$R_0 = k[\text{complex}][H_2O_2] \qquad (5)$$

This reaction order is obtainable when the concentration of the complex is kept constant and [H_2O_2]>>[complex]. From the measurement of the kinetics at different hydrogen peroxide concentrations, different R_0 are obtained. The observable rate constant is determined with *Equation 6*.

$$k_{obs} = R_0/[\text{complex}] = k_{CAT}[H_2O_2] \qquad (6)$$

Finally, in the plot "*k_{obs} vs [H_2O_2] concentration*", the slope of the curve is k_{CAT}.

[15] Squarcina, A.; Santoro, A.; Hickey, N.; De Zorzi, R.; Carraro, M.; Geremia, S.; Bortolus, M.; Di Valentin, M.; Bonchio, M.; *ACS Catal.*, **2020**, 10, 7295–7306

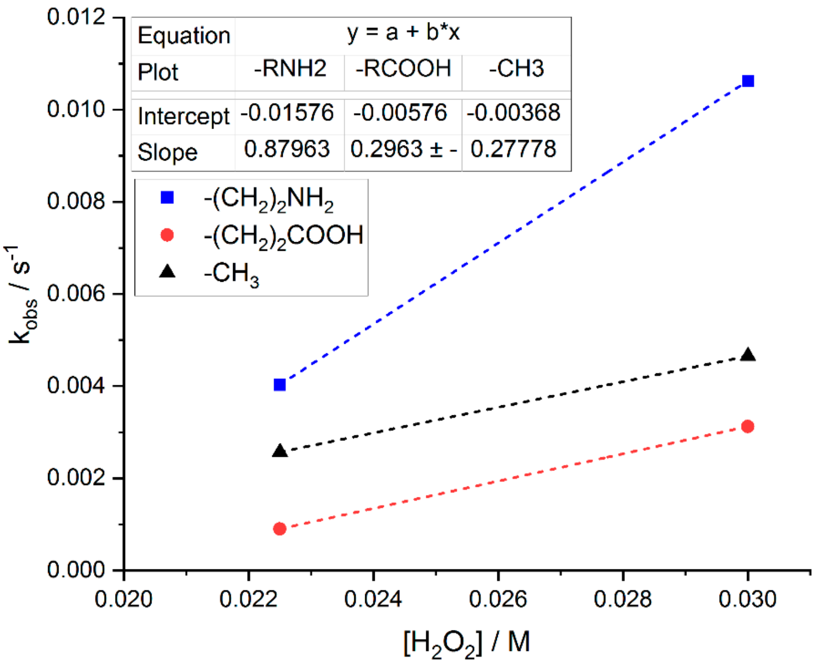

Figure S48: *Plot k_{obs} vs [H_2O_2] for determination of k_{CAT}.*

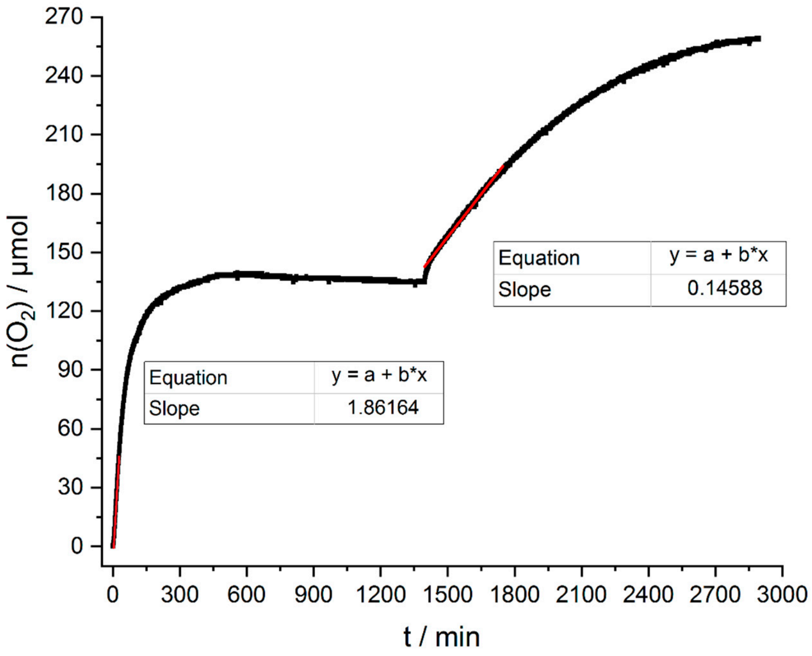

Figure S49: *O_2 evolution from hydrogen peroxide vs time including a second addition of H_2O_2 after the first 22 hours. [Cu_2(L-RNH_2)(H_2O)$_2$](ClO_4)$_3$ concentration=200 µM, [H_2O_2]=30 mM in BBS, pH 7.8, T=25 °C, head-space 12.98 mL. Note that slopes (R_0) are in µmol min^{-1}. Dividing by the volume and by 60 gives it in µM O_2 s^{-1}.*

Table S3. Literature comparison for CAT activity in water under physiological conditions. a) pH 11. L^2: 2-({[di(2-pyridyl)methyl]amino}-methyl)phenol). L′: L′: 2-{[[di(2-pyridyl)methyl](methyl) amino]-methyl}phenol).

Complexes	R_0 (μM O_2 s^{-1})	TOF (s^{-1})	TON	k_{CAT} (M^{-1} s^{-1})	Ref.
**Mn_2-CAT$_{(T.thermophilus)}$**	-	-	-	$3.1\cdot10^6$	[16]
**$Mn_2L'_2P2^{6+}$**	36	0.73	-	1890	[11]
**$[Cu_2(L\text{-}CH_3)(H_2O)_2](ClO_4)_3$** [a]	0.51	0.0026	35	0.28	this work
**$[Cu_2(L\text{-}RNH_2)(H_2O)_2](ClO_4)_3$** [a]	0.81	0.0040	38	0.88	this work
**$[Cu_2(L\text{-}RCOOH)(H_2O)_2](ClO_4)_3$** [a]	0.18	0.0009	43	0.30	this work
**$[Cu(PBMPA)]^+$**	14.4[a]	-	125[a]	0.030[a]	[12]
**$[Cu(apz\text{-}pn)]^{2+}$**	-	-	-	1.10	[17]
**$Cu_2L^2_2$**	4.4	-	60	0.65	[13]
**(CATm2)Cu_2** [b]	-	-	7	3.9	[18]
**CuL_2** [c]	-	-	-	8.25	[19]

[a] Conditions: [complex]=200 μM, $[H_2O_2]$=22.5 mM in BBS pH 7.8 and T=25 °C.

[b] the ligand CATm2 is a peptide sequence: Ac(PHYKH)(PHYKH)-NH_2.

[c] L=2-{[(3-chloro-2-hydroxy-propyl)-pyridin-2-ylmethyl-amino]-methyl}-phenol

[16] Lück, H.; Catalase. *Methods of Enzymatic Analysis,* **1965**, 885–894.
[17] Pires dos Santos, M. L.; Faljoni-Alário, A.; Mangrich, A. S.; Costa Ferreira, A. M. da; *J. Inorg. Biochem.*, **1998**, 71, 71–78.
[18] Ben Hadj Hammouda, Y., Coulibaly, K., Bathily, A., Teoh Sook Han, M., Policar, C., Delsuc, N. *Molecules*. **2022**; 27(17):5476.
[19] Guerreiro, J.F., Gomes, M.A.G.B., Pagliari, F., Jansen, J., Marafioti, M.G., Nistico, C., Hanley, R., Costa, R.O., Ferreira, S.S., Mendes, F., Fernandes, C., Horn, A., Tirinato, L., Seco, J. *RSC Adv.*, **2020**, 10, 12699-12710

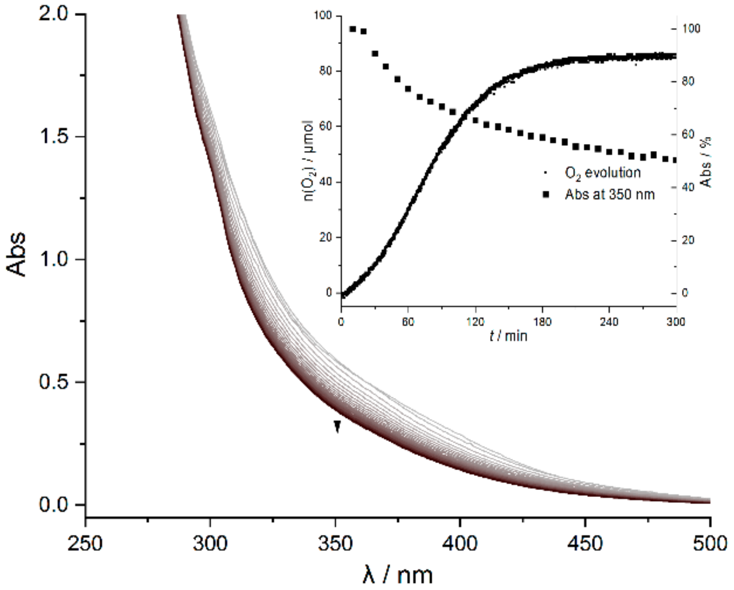

Figure S50: *UV-vis spectra of [Cu_2(L-CH_3)(H_2O)$_2$](ClO_4)$_3$ in a CAT assay measured every 10 minutes and centered at 350 nm. [Complex]=200 µM, [H_2O_2]=30 mM in BBS, pH 7.8, T=25 °C. The arrow indicates the absorbance trend over time. Inset: oxygen evolution (line) and absorbance at 350 nm (dots) versus time.*

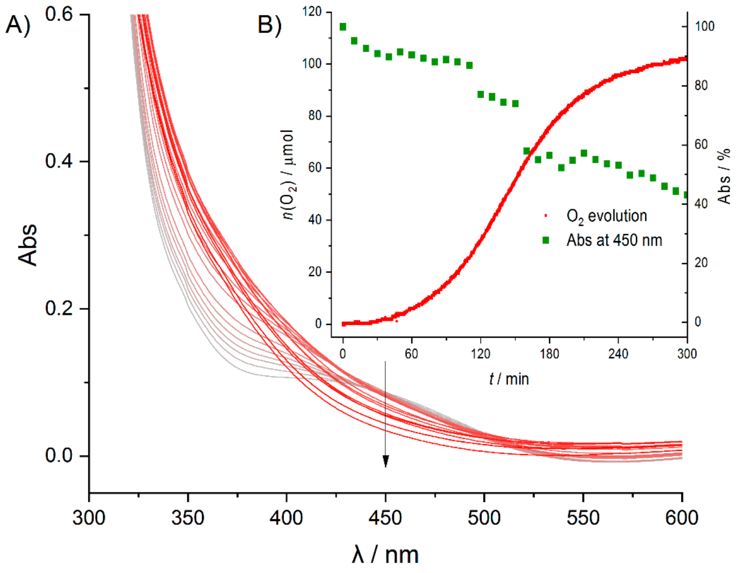

Figure S51. *A) UV-vis spectra of [Cu_2(L-RCOOH)(H_2O)$_2$](ClO_4)$_3$ in a CAT assay measured every 10 minutes and centered at 450 nm. [Complex]=200 µM, [H_2O_2]=30 mM in BBS, pH 7.8, T=25 °C. The arrow indicates the absorbance trend over time. B) Oxygen evolution (red line) and absorbance at 450 nm (green dots) versus time.*

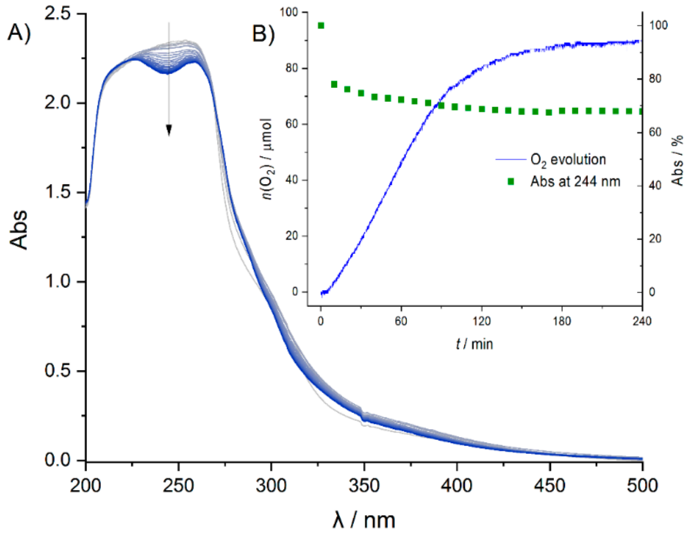

Figure S52: *A) UV-vis spectra of [Cu_2(L-RNH_2)(H_2O)$_2$](ClO_4)$_3$ in a CAT assay measured every 10 minutes and centered at 450 nm. [Complex]=200 µM, [H_2O_2]=30 mM in BBS, pH 7.8, T=25 °C. The arrow indicates the absorbance trend over time. B) Oxygen evolution (blue line) and absorbance at 244 nm (green dots) versus time.*

Peroxidase-like activity

For the peroxidase-like activity the variation of absorbance over time was monitored via UV-vis spectroscopy. The substrate OPD (*o*-phenylenediamine) was oxidized to DAP (2,3-diaminephenazine) by H_2O_2 in the presence of copper ions, leading to an increase of absorbance at 418 nm. For evaluating this kinetic, a solution 0.32 mM of OPD in PBS (50 mM, 150 mM NaCl, pH 7.8) was prepared. The copper complex (or salt) and hydrogen peroxide were added to this solution at the concentration of 3.2 µM and 24 mM respectively. The kinetic was recorded in a quartz cuvette with an optical path of 1 cm for 18h (acquiring a spectrum every 20 minutes) at 25°C. The graph "*Abs at 418 nm vs time*" was then built. Each assay was performed twice.

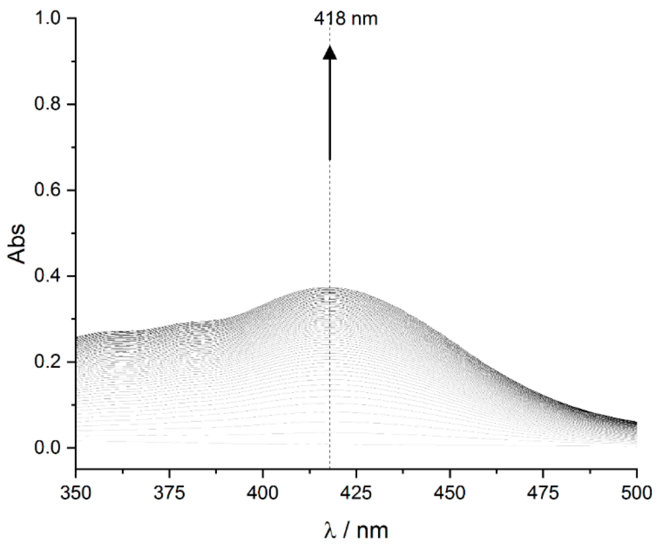

Figure S53: *Variation of absorbance at 418 nm vs time (18h, a spectrum every 20 minutes) for the complex [Cu_2(L-CH_3)(H_2O)$_2$](ClO_4)$_3$ in the peroxidase-like assay.*

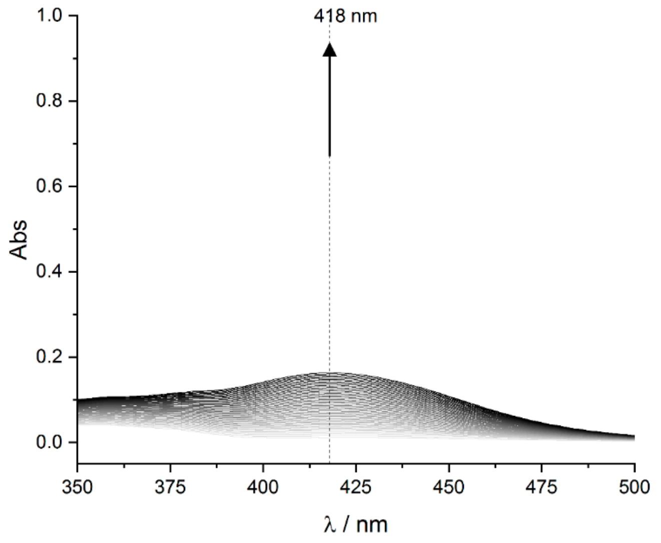

Figure S54: *Variation of absorbance at 418 nm vs time (18h, a spectrum every 20 minutes) for the complex [Cu_2(L-RCOOH)(H_2O)$_2$](ClO_4)$_3$ in the peroxidase-like assay.*

Aggregation test: ThT method

Amyloid peptides Aβ1-40 were used for this analysis. 1 mg of the amyloid peptide was dissolved in 1 mL of 1,1,1,3,3,3-Hexafluoro-2-propanol and stirred for about 12 hours at 4°C. Solvent was evaporated with a soft stream of N_2 and re-dissolved in 10mM phosphate buffer (NaCl 100mM, pH=7.4), to obtain a concentration of 77 μM (3 mL, MW = 4329.82 g/mol).

A ThT fresh solution 0.04 mM was prepared in 10 mM PBS buffer (NaCl 100mM, pH= 7.4), from a mother solution 5mM in water. The concentration was checked by Uv-vis spectroscopy, from the molar extinction coefficient (ε) of the band at 412 nm, equal to 36000 $M^{-1}cm^{-1}$.

The solutions 0.2 mM of copper, ligands and preformed complexes were prepared in methanol.

- For the measurement, fluorescence spectra were recorded with a plate reader, using a Tecan Infinite instrument.

was used: in each cockpit (300 μL), the solutions were organized as follows:

1. ThT 10 μM, Aβ 10 μM;
2. ThT 10 μM, Aβ 10 μM; HL-CH_3 10 μM;
3. ThT 10 μM, Aβ 10 μM, $Cu_2(HL\text{-}CH_3)(H_2O)(ClO_4)_3$ 10 μM;

Every solution was brought up to volume with PBS buffer 10 mM (NaCl 100mM, pH = 7.4).

The fluorescence of sample solutions was monitored at 37° C over a period of about 30 hours, acquiring values every 20 minutes. The fluorescence variation of ThT was compared in the presence/ absence of amyloid/ ligands/ complexes.

Cartesian Coordinates for the CuL complex of (HL-CH_3)

C	1.93185	-2.18949	1.46338
C	2.02502	-3.03952	0.35871
C	1.11072	-2.90413	-0.69472
C	0.09893	-1.92238	-0.66148
C	-0.00223	-1.09015	0.49117
C	0.91813	-1.22774	1.52946
O	-0.98966	-0.17820	0.65894
C	3.09826	-4.08723	0.29395
C	-0.86219	-1.82360	-1.84905
C	0.81940	-0.29755	2.69811
N	-0.65696	-0.57487	-2.62995
N	-0.45862	-0.45851	3.43906
C	1.99245	2.84198	-2.01772
N	1.15968	1.79244	-2.25110
C	1.55401	0.70119	-2.99511
C	2.85287	0.69377	-3.53178
C	3.71292	1.76652	-3.29849
C	3.28356	2.84692	-2.53428
C	-4.22428	-0.46889	-3.11197
C	-2.99751	0.21066	-3.03688
N	-2.95193	1.44229	-2.44391
C	-4.05856	2.02906	-1.91503
C	-5.29020	1.37947	-1.97129
C	-5.37143	0.12428	-2.57503
C	-2.20025	4.01617	2.27531
C	-2.41377	2.70161	1.84883
N	-1.88245	1.67033	2.56010
C	-1.12640	1.87587	3.68097
C	-0.90641	3.17791	4.14243
C	-1.44802	4.25110	3.43072
C	-1.84846	-4.95292	2.29392
C	-2.65204	-4.37192	1.30819
C	-2.76590	-2.98202	1.25041
N	-2.08702	-2.21601	2.14597
C	-1.28325	-2.75373	3.10870
C	-1.16214	-4.14448	3.20574
C	-0.55816	0.66666	4.40463
C	-0.52905	-1.82058	4.04367
C	-1.73419	-0.37711	-3.63793
C	0.69469	-0.54186	-3.27263
Cu	-2.05615	-0.26884	2.26261
O	-3.65607	-0.09891	1.17636
O	-3.24522	-0.35978	3.79320
H	2.64193	-2.28054	2.27701
H	1.19770	-3.56434	-1.54954
H	3.74379	-4.06900	1.19868
H	3.73974	-3.91376	-0.59513
H	2.63389	-5.09289	0.21977
H	-1.90200	-1.89419	-1.46156
H	-0.72416	-2.69354	-2.53159
H	0.91596	0.73704	2.29664
H	1.68081	-0.44694	3.39027
H	-0.72681	0.18716	-1.95608
H	1.64990	3.67985	-1.42182
H	0.22433	1.90086	-1.81741

H	3.20150	-0.14567	-4.12091
H	4.71465	1.75771	-3.70596
H	3.94276	3.68270	-2.34174
H	-4.29690	-1.43948	-3.58575
H	-2.05206	1.96789	-2.39545
H	-3.98345	3.00583	-1.45350
H	-6.17214	1.84904	-1.55480
H	-6.32526	-0.38427	-2.63020
H	-2.62023	4.84574	1.72159
H	-3.00340	2.51441	0.96409
H	-0.32035	3.35778	5.03521
H	-1.28332	5.26446	3.77403
H	-1.75254	-6.02946	2.34614
H	-3.18007	-4.99221	0.59564
H	-3.38230	-2.52280	0.49068
H	-0.53068	-4.59378	3.96142
H	-1.22750	0.44753	5.26006
H	0.43140	0.89619	4.86367
H	-1.04026	-1.83858	5.02767
H	0.49101	-2.21835	4.24824
H	-1.41420	0.36441	-4.40338
H	-1.93853	-1.33222	-4.17498
H	1.30961	-1.40205	-2.93310
H	0.61731	-0.68992	-4.37427
H	-3.52980	0.25772	0.26555
H	-4.24707	0.56207	1.61585
H	-2.91265	-0.81120	4.60252
H	-3.54430	0.54177	4.07191

Cartesian Coordinates for the Cu_2L complex of ($HL-CH_3$)

Structure 2a

C	2.01651	-2.20152	1.51563
C	2.14303	-2.86479	0.28567
C	1.25410	-2.55925	-0.76608
C	0.21286	-1.61803	-0.58768
C	0.06853	-1.05663	0.69467
C	0.97484	-1.29123	1.71085
O	-0.97822	-0.28627	0.97055
C	3.21068	-3.90512	0.09748
C	-0.86330	-1.39457	-1.66489
C	0.83723	-0.47771	2.96609
N	-0.68463	-0.41070	-2.80866
N	-0.48178	-0.60184	3.65410
C	1.79846	2.83444	-2.35681
N	1.02970	1.76551	-2.69847
C	1.55927	0.59159	-3.14689
C	2.94529	0.48858	-3.31649
C	3.75749	1.57776	-2.98290
C	3.18519	2.75427	-2.49039
C	-4.34656	-0.46645	-3.25836
C	-3.05468	0.06909	-3.21657
N	-2.85319	1.31201	-2.69829
C	-3.87448	2.07117	-2.21302
C	-5.17975	1.57212	-2.24830
C	-5.41292	0.29661	-2.77279
C	-2.17937	3.88823	2.44041
C	-2.42969	2.57014	2.05541

N	-1.90150	1.54788	2.78175
C	-1.12260	1.75401	3.88229
C	-0.86232	3.06418	4.30288
C	-1.39416	4.13317	3.57148
C	-1.87459	-5.06559	2.41613
C	-2.66319	-4.47568	1.42593
C	-2.78323	-3.08590	1.38342
N	-2.12822	-2.32228	2.30283
C	-1.33776	-2.87452	3.27183
C	-1.20979	-4.26599	3.34919
C	-0.56820	0.53643	4.61405
C	-0.60413	-1.96256	4.24341
C	-1.83938	-0.68696	-3.71773
C	0.63100	-0.56484	-3.49325
Cu	-2.08027	-0.36604	2.46100
Cu	-0.90775	1.63924	-2.66092
O	-3.60407	-0.10856	1.31871
O	-1.11833	3.55357	-2.53981
O	-3.25853	-0.52470	3.99200
O	-0.89044	1.80100	-4.58273
O	-0.89863	1.64778	-0.80047
H	2.71634	-2.39861	2.31909
H	1.35475	-3.09427	-1.70136
H	3.81881	-4.03873	1.01890
H	3.88614	-3.61802	-0.73330
H	2.73590	-4.88237	-0.13710
H	-1.83487	-1.21013	-1.16426
H	-1.00725	-2.41968	-2.08101
H	0.98908	0.57604	2.63509
H	1.67108	-0.70060	3.67118
H	1.34400	3.74390	-1.98913
H	3.38983	-0.42257	-3.69530
H	4.83062	1.51016	-3.10359
H	3.80858	3.59847	-2.22494
H	-4.52504	-1.45473	-3.66209
H	-3.68529	3.03985	-1.77426
H	-6.00072	2.15967	-1.85739
H	-6.41881	-0.10243	-2.79693
H	-2.58967	4.71102	1.86829
H	-3.03472	2.37082	1.18171
H	-0.25091	3.25554	5.17489
H	-1.19609	5.15218	3.87787
H	-1.77278	-6.14209	2.45502
H	-3.17421	-5.08948	0.69534
H	-3.38702	-2.63544	0.61114
H	-0.58923	-4.72280	4.10927
H	-1.23065	0.33660	5.47653
H	0.42028	0.76197	5.07753
H	-1.15600	-1.97517	5.20388
H	0.39413	-2.38486	4.49801
H	-1.64984	-0.31917	-4.74136
H	-2.04436	-1.76744	-3.89152
H	1.10278	-1.53797	-3.26560
H	0.54149	-0.55595	-4.60098
H	-3.38556	-0.37790	0.39270
H	-1.82718	3.82652	-3.17375
H	-2.91568	-0.80794	4.86754
H	-1.77168	2.16200	-4.85555
H	-1.70329	1.14451	-0.54314

H	-3.69979	0.34966	4.14064
H	-0.25801	2.53171	-4.79923
H	-1.47564	3.79651	-1.64830
H	-0.07307	1.18443	-0.52923
H	-4.32051	-0.72350	1.61480

Cartesian Coordinates for the Cu_2L complex of ($HL-CH_3$)

Structure 2b

C	2.00760	-2.03113	1.41291
C	2.07969	-3.01093	0.42472
C	1.19350	-2.94540	-0.64874
C	0.24410	-1.90470	-0.72645
C	0.14918	-0.92743	0.25625
C	1.03438	-1.01533	1.33363
O	-0.83894	0.02291	0.20627
C	3.05481	-4.14711	0.53057
C	-0.76225	-1.90872	-1.79454
C	0.89059	-0.06006	2.43891
N	-0.71753	-0.69933	-2.64221
N	-0.37773	-0.32317	3.15582
C	1.65561	2.49585	-1.53915
N	1.00095	1.34801	-1.89842
C	1.50318	0.48646	-2.82128
C	2.78430	0.74135	-3.35619
C	3.46925	1.91224	-2.99294
C	2.89442	2.80067	-2.09046
C	-4.46599	-0.29206	-3.21091
C	-3.12334	-0.08213	-2.83429
N	-2.83669	0.90320	-1.93730
C	-3.80684	1.76560	-1.50361
C	-5.12992	1.59824	-1.86158
C	-5.46910	0.54534	-2.69766
C	-2.24068	4.16919	2.38819
C	-2.46730	2.88779	1.87356
N	-1.85317	1.80338	2.41744
C	-1.08021	1.94687	3.54114
C	-0.86581	3.20316	4.10943
C	-1.43041	4.32349	3.51175
C	-1.79472	-4.87538	2.09733
C	-2.58801	-4.32219	1.09104
C	-2.65564	-2.93085	0.95845
N	-1.93397	-2.12931	1.79626
C	-1.14932	-2.65045	2.78373
C	-1.07584	-4.03979	2.95428
C	-0.49807	0.72389	4.18697
C	-0.40524	-1.71062	3.71653
C	-1.95626	-0.83240	-3.48231
C	0.62284	-0.65852	-3.33603
Cu	-1.97720	-0.15283	1.97142
Cu	-0.89220	1.07612	-1.65448
O	-3.56558	-0.08983	0.95646
O	-0.99749	2.70421	-0.72076
O	-3.10932	-0.34516	3.49661
O	-0.98086	2.06400	-3.33111
H	2.66978	-2.08694	2.26993
H	1.22199	-3.72371	-1.40357
H	3.67885	-4.06539	1.44604

H	3.72056	-4.15741	-0.35612
H	2.49885	-5.10882	0.57937
H	-1.74982	-2.00394	-1.30571
H	-0.62957	-2.82039	-2.42569
H	0.92977	0.97142	2.02266
H	1.74407	-0.16285	3.14880
H	1.20605	3.24990	-0.91000
H	3.22855	0.08823	-4.09377
H	4.42421	2.16368	-3.44174
H	3.37947	3.74306	-1.85426
H	-4.73777	-1.07403	-3.90410
H	-3.60046	2.61617	-0.91022
H	-5.87086	2.30200	-1.49794
H	-6.50576	0.41907	-2.97381
H	-2.69982	5.03494	1.93031
H	-3.12284	2.77072	1.05048
H	-0.26199	3.30635	5.00220
H	-1.25479	5.30436	3.93289
H	-1.74883	-5.95008	2.22315
H	-3.16541	-4.96691	0.43892
H	-3.31163	-2.50966	0.21094
H	-0.47260	-4.46807	3.74442
H	-1.16081	0.44876	5.03014
H	0.47737	0.93436	4.68035
H	-0.91557	-1.77144	4.70073
H	0.62689	-2.08824	3.88837
H	-1.81672	-0.44822	-4.50897
H	-2.25760	-1.89614	-3.65094
H	1.21496	-1.59025	-3.17826
H	0.50197	-0.55394	-4.42974
H	-3.32337	-0.10784	0.01846
H	-0.77110	2.46767	0.20518
H	-2.88715	-0.62741	4.41264
H	-0.91709	1.49307	-4.12689
H	-1.80903	2.59313	-3.43915
H	-1.73308	3.35904	-0.72751
H	-4.17224	0.66562	1.14486
H	-4.08706	-0.28054	3.35794
